# Spectacle Lenses With Aspherical Lenslets for Myopia Control vs Single-Vision Spectacle Lenses: A Randomized Clinical Trial

**DOI:** 10.1001/jamaophthalmol.2022.0401

**Published:** 2022-03-31

**Authors:** Jinhua Bao, Yingying Huang, Xue Li, Adeline Yang, Fengchao Zhou, Junqian Wu, Chu Wang, Yuhao Li, Ee Woon Lim, Daniel P. Spiegel, Björn Drobe, Hao Chen

**Affiliations:** 1 Eye Hospital and School of Ophthalmology and Optometry, Wenzhou Medical University, Wenzhou, Zhejiang, China; 2 National Clinical Research Center for Ocular Diseases, Wenzhou, Zhejiang, China; 3 Wenzhou Medical University–Essilor International Research Center (WEIRC), Wenzhou Medical University, Wenzhou, Zhejiang, China; 4 R&D AMERA, Essilor International, Singapore, Singapore

## Abstract

**Importance:**

Reducing myopia progression can reduce the risk of associated ocular pathologies.

**Objective:**

To evaluate whether spectacle lenses with higher lenslet asphericity have a higher myopia control efficacy throughout 2 years.

**Design, Setting, and Participants:**

This double-masked randomized clinical trial was conducted between July 2018 and October 2020 at the Eye Hospital of Wenzhou Medical University in Wenzhou, China. Children aged 8 to 13 years with a cycloplegic spherical equivalent refraction (SER) of −0.75 D to −4.75 D and astigmatism with less than −1.50 D were recruited. A data and safety monitoring committee reviewed findings from a planned interim analysis in 2019.

**Interventions:**

Participants were randomly assigned in a 1:1:1 ratio to receive spectacle lenses with highly aspherical lenslets (HAL), spectacle lenses with slightly aspherical lenslets (SAL), or single-vision spectacle lenses (SVL).

**Main Outcome and Measures:**

Two-year changes in SER and axial length and their differences between groups.

**Results:**

Of 157 participants who completed each visit (mean [SD] age, 10.4 [1.2] years), 54 were analyzed in the HAL group, 53 in the SAL group, and 50 in the SVL group. Mean (SE) 2-year myopia progression in the SVL group was 1.46 (0.09) D. Compared with SVL, the mean (SE) change in SER was less for HAL (by 0.80 [0.11] D) and SAL (by 0.42 [0.11] D; *P* ≤ .001). The mean (SE) increase in axial length was 0.69 (0.04) mm for SVL. Compared with SVL, increase in axial length was slowed by a mean (SE) of 0.35 (0.05) mm for HAL and 0.18 (0.05) mm for SAL (*P* ≤ .001). Compared with SVL, for children who wore HAL at least 12 hours every day, the mean (SE) change in SER was slowed by 0.99 (0.12) D, and increase in axial length slowed by 0.41 (0.05) mm.

**Conclusions and Relevance:**

In this study, HAL and SAL reduced the rate of myopia progression and axial elongation throughout 2 years, with higher efficacy for HAL. Longer wearing hours resulted in better myopia control efficacy for HAL.

**Trial Registration:**

Chinese Clinical Trial Registry Identifier: ChiCTR1800017683

## Key Points

**Question:**

What is the efficacy of spectacle lenses with highly aspherical lenslets and slightly aspherical lenslets compared with conventional single-vision spectacle lenses in controlling myopia progression throughout 2 years?

**Findings:**

In this randomized clinical trial of 157 children, the amount of myopia progression and axial length increase was significantly less in both the highly aspherical lenslets group and the slightly aspherical lenslets group vs the single-vision spectacle lenses group at 24 months.

**Meaning:**

The findings of this study suggest that a higher asphericity of lenslets may be associated with more effective myopia control.

## Teaser

This randomized clinical trial aimed to evaluate whether spectacle lenses with aspherical lenslets slow myopia progression over 2 years and whether the level of lenslet asphericity affects myopia control efficacy in a dose-dependent manner.

## Introduction

The projected 2050 prevalence rates of myopia and high myopia are alarming.^1^ Myopia control interventions have been used for many years to reduce the severity of myopia and decrease the risk of associated ocular pathologies.^2^ Increasing evidence suggests that specifically designed optical interventions such as spectacle lenses, soft contact lenses, and orthokeratology slow myopia progression in children.^3,4,5^ The common features of these myopia optical interventions are to provide central correction for distance vision and correct peripheral retinal defocus or induce peripheral myopic retinal defocus simultaneously. Peripheral visual signals have been found to dominate central refractive development,^6^ and the effect of peripheral myopic retinal defocus was found to provide myopia control signals.^7,8,9^ Furthermore, several studies have shown a positive dose-response relationship between the efficacy of optical interventions and parameters such as addition power in multifocal soft contact lenses^10^ and spectacle lenses^11,12^ and in the wearing time of multifocal soft contact lenses.^13^ This study evaluates novel spectacle lenses with aspherical lenslets and explores the effect of lenslet asphericity on myopia control efficacy. The results from the first-year interim analysis showed that spectacle lenses with highly aspherical lenslets (HAL) and spectacle lenses with slightly aspherical lenslets (SAL) were effective in slowing myopia progression. Moreover, a dose-dependent response was observed, as HAL had significantly better myopia control effect than SAL.^14^

We aimed to evaluate whether spectacle lenses with aspherical lenslets still slow myopia progression over 2 years and whether the level of lenslet asphericity would still affect myopia control efficacy in a dose-dependent manner.

## Methods

### Study Design

This 2-year, double-masked, 3-group, parallel randomized, single-center clinical trial comparing the effect of spectacle lenses with aspherical lenslets with single-vision spectacle lenses (SVL) in slowing myopia progression among children was conducted in the Eye Hospital of Wenzhou Medical University in Wenzhou, China, from July 18, 2018, to October 7, 2020. The study design has been described previously.^14^ The trial protocol, standard operating procedures, and statistical analysis plan are available in Supplement 1, Supplement 2, and Supplement 3, respectively. Children were randomly assigned to wear HAL, SAL, or SVL based on their baseline right eye cycloplegic spherical equivalent refraction (SER), age, and sex. Assignment with online software (Randola^15^) was performed by a specified person who had no interaction with the participants and stored on the website that required login credentials. The same person would pack the lenses according to the randomization in an unlabeled envelope with participant identification as the only identification for dispensing. Masking was maintained for refraction and axial length measurements for dispensing personnel and examiners, participants, and parents or guardians. However, it was not entirely possible to mask HAL and SAL lenses from SVL because lenslets were visible under certain lighting conditions. However, complete masking was possible between HAL and SAL. Written informed consent was obtained from the parent or guardian, and written assent was obtained from the study participant. All study participants were given a pair of myopia-control spectacles on completion of the study. A data and safety monitoring committee reviewed the trial data for participant safety. The ethics committee of Eye Hospital of Wenzhou Medical University approved the clinical trial on July 17, 2018, and the trial was registered with the Chinese Clinical Trial Registry. All procedures adhered to the tenets of the Declaration of Helsinki.^16^

### Participants

The first participant was enrolled on August 11, 2018. Chinese children aged 8 to 13 years who had myopia, SER from −0.75 diopters (D) to −4.75 D, astigmatism of 1.50 D cylinder or less, anisometropia of 1.00 D or less, and best-corrected visual acuity of 0.05 logMAR or better in each eye were randomized at a 1:1:1 ratio to wear HAL, SAL, or SVL. No participants had a history of myopia control or any ocular or systemic issues that could affect vision outcomes.

### Study Procedures

A detailed description of this study device can be found elsewhere.^14^ The primary outcomes were changes in SER and axial length. The parameter measurement procedures followed those for the 1-year outcomes of HAL and SAL.^14^ SER and axial length were measured every 6 months. SER was measured by the mode of 10 measurements using a Topcon KR-800 (Topcon Corporation), which was acquired at least 30 minutes after installing 2 drops of cyclopentolate, 1%, administered 5 minutes apart. Axial length was calculated as the mean of 5 measurements obtained using a Lenstar LS 900 instrument (Haag-Streit AG).

The secondary outcomes presented in this report were lens wearing hours assessed by 6-month questionnaires with self-reported wearing times per day, every day per week (Monday to Sunday).

### Statistical Analysis

Based on previous findings,^12,17,18^ we anticipated a mean (SD) SER progression of 1.50 (0.75) D and converted axial length progression of 0.6 (0.02) mm in the control group throughout a 2-year period. We wanted to identify a 33% reduction in the amount of SER and axial length progression for treatment groups compared with the control group. Fifty participants were required in each group for an α level of .05 (2-tailed), a power of 90%, and a dropout rate of 10%. With an interim analysis at 1 year, the α level was adjusted to .029 based on the Pocock method for 2-year outcomes of SER and axial length.

All data from participants who had complete 2-year follow-up records were analyzed. The mean values for ocular parameters measured in the right eye were used because there was a high correlation between the 2 eyes in SER (*r* = 0.74, *P* < .001) and axial length (*r* = 0.87, *P* < .001). The changes in SER and axial length from baseline are presented as mean (SE). χ^2^ Test and analysis of variance with post hoc Bonferroni test were used to assess intergroup differences in categorical and continuous variables, respectively. Our analysis was performed using complete case data without imputation for missing data and dropouts. We undertook analyses using linear mixed model, adjusted for baseline age, sex, SER, axial length, age at myopia onset, and number of parents with myopia to evaluate the treatment effect. The mean daily lens wearing hours were calculated based on the mean daily lens wearing hours over the four 6-month periods. The effect of wearing time was analyzed categorically. Full-time wearers were defined as children who reported wearing their study devices for at least 12 hours every day. Part-time wearers were defined as nonfull-time wearers. SPSS statistical software version 24 (IBM) was used for data analysis. Two-sided *P* values of less than .05 were considered statistically significant.

## Results

**Figure 1.**
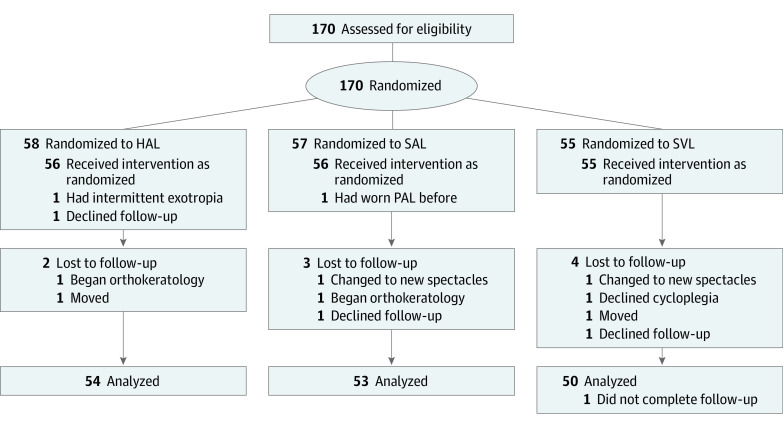
CONSORT Study Flowchart HAL indicates spectacle lenses with highly aspherical lenslets; PAL, progressive addition lenses; SAL, spectacle lenses with slightly aspherical lenslets; SVL, single-vision spectacle lenses.

One hundred seventy children with a mean (SD) age of 10.4 (1.2) years, ranging from 8 to 13 years, were recruited and randomized among the HAL (n = 58), SAL (n = 57), and SVL (n = 55) groups (Figure 1). Only 167 were dispensed with the study equipment. Three children discontinued the study: 1 presented with intermittent exotropia not apparent during screening, 1 belatedly reported history of using myopia control, and 1 dropped out before test lenses were dispensed. After 2 years, 157 participants had completed all their visits; among children who did not attend their follow-up appointments, 2 (3.6%), 3 (5.4%), and 4 (7.3%) were in the HAL, SAL, and SVL groups, respectively. The reasons for dropout were not related to study devices. The demographic and ocular characteristics of each group at baseline are shown in Table 1.

**Table 1.** Baseline Demographic and Ocular Characteristics of Participants Who Completed the 24-Month Follow-up in Each Group

Clinical value	Mean (SE)		
	HAL (n = 54)	SAL (n = 53)	SVL (n = 50)
Age, y	10.6 (0.2)	10.2 (0.2)	10.4 (0.2)
Female, No. (%)	28 (52)	36 (68)	21 (42)
Male, No. (%)	26 (48)	17 (32)	29 (58)
Right eye			
Refractive error (SER), D	−2.70 (0.14)	−2.28 (0.13)	−2.44 (0.12)
Axial length, mm	24.76 (0.09)	24.44 (0.10)	24.77 (0.09)
Age at myopia onset, y	9.3 (0.2)	9.4 (0.2)	9.3 (0.2)
Parents with myopia, No. (%)			
0	18 (33.3)	12 (22.6)	12 (24.0)
1	20 (37.0)	22 (41.5)	18 (36.0)
2	16 (29.6)	19 (35.8)	20 (40.0)

Abbreviations: D, diopters; HAL, spectacle lenses with highly aspherical lenslets; SAL, spectacle lenses with slightly aspherical lenslets; SER, spherical equivalent refraction; SVL, single-vision spectacle lenses.

### Primary Outcome

#### Changes in Spherical Equivalent Over 2 Years

**Figure 2.**
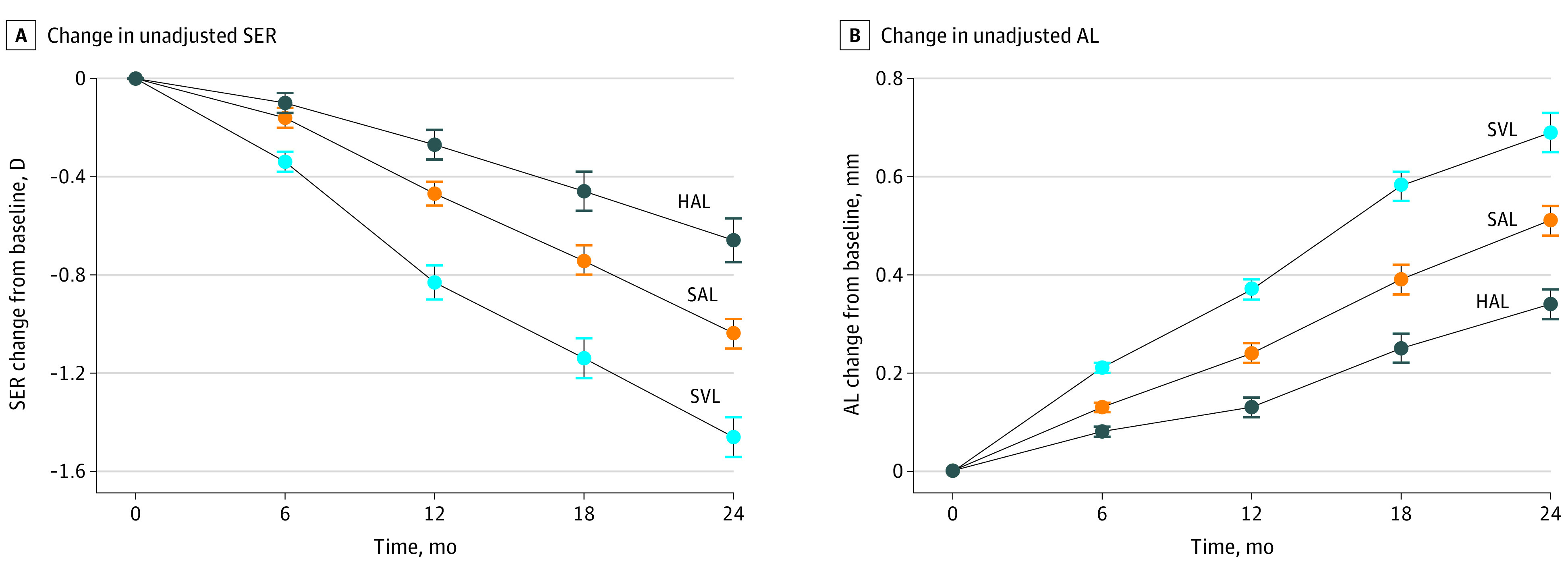
Change in Unadjusted Spherical Equivalent Refraction (SER) and Axial Length (AL) From Baseline to 2 Years Error bars represent standard errors of the mean. HAL indicates spectacle lenses with highly aspherical lenslets; SAL, spectacle lenses with slightly aspherical lenslets; SVL, single-vision spectacle lenses.

The 2-year means (SEs) for myopia progression were −0.66 (0.09) D, −1.04 (0.06) D, and −1.46 (0.09) D in the HAL, SAL, and SVL groups, respectively (Figure 2). Significant differences were found among treatment groups (*F*_2,154_ = 25.80; *P* < .001). The HAL and SAL groups had less SER progression by a mean (SE) of 0.80 (0.11) D (95% CI, 0.53-1.07; *P* < .001) and 0.42 (0.11) D (95% CI, 0.15-0.70; *P* = .001), respectively, than the SVL group. In addition, the HAL group had less SER progression than the SAL group, with a mean (SE) difference of 0.38 (0.11) D (95% CI, 0.11-0.64; *P* = .002).

In the linear mixed model analysis, baseline age (95% CI, 0.004-0.128; *P* = .04) was directly, significantly associated with SER progression. The model-adjusted mean (SE) changes in SER were −0.68 (0.07) D, −1.04 (0.08) D, and −1.45 (0.08) D for HAL, SAL, and SVL, respectively, with a significant effect of lens design (*F*_6,154_ = 7.88; *P* < .001) on SER. Compared with SVL, the adjusted differences in mean (SE) SER were 0.77 (0.11) D (95% CI, 0.51-1.04; *P* < .001) and 0.42 (0.11) D (95% CI, 0.15-0.68; *P* = .001) in the HAL and SAL groups, respectively, and 0.36 (0.11) D (95% CI, 0.10-0.62; *P* = .003) between the HAL and SAL groups.

#### Changes in Axial Length Over 2 Years

The mean (SE) increases in axial length over 2 years were 0.34 (0.03) mm, 0.51 (0.04) mm, and 0.69 (0.04) mm in the HAL, SAL, and SVL groups, respectively (Figure 2). As with SER, there was a significant difference (*F*_2,154_ = 24.98; *P* < .001) in axial length increase among treatment groups. Compared with the SVL group, the HAL and SAL groups had reduced mean (SE) axial length elongation by 0.35 (0.05) mm (95% CI, 0.23-0.47; *P* < .001) and 0.18 (0.05) mm (95% CI, 0.06-0.30; *P* = .001), respectively. Furthermore, HAL had less axial length elongation than SAL by a mean (SE) of 0.17 (0.05) mm (95% CI, 0.05-0.29; *P* = .002).

In the linear mixed model analysis, baseline age (95% CI, −0.04 to −0.01; *P* = .002) and age at myopia onset (95% CI, 0.003-0.032; *P* = .02) were significantly associated with axial length elongation. After adjustment, the means (SEs) for change in axial length were 0.35 (0.03) mm, 0.50 (0.03) mm, and 0.68 (0.03) mm for HAL, SAL, and SVL, respectively, with 0.34 (0.05) mm (95% CI, 0.23-0.45; *P* < .001) and 0.18 (0.05) mm (95% CI, 0.07-0.30; *P* < .001) reductions in axial length elongation in the HAL and SAL groups compared with SVL. The HAL group had less axial length increase than SAL by a mean (SE) of 0.16 (0.05) mm (95% CI, 0.05-0.27; *P* = .002). As with SER, there was a significant effect of lens design (*F*_6,154_ = 8.41; *P* < .001) in axial length increase among treatment groups throughout 2 years.

### Secondary Outcomes

During the second year, HAL still slowed myopia progression compared with SVL (mean [SE] for HAL, SER: −0.39 [0.05] D; *P* < .001; axial length, 0.21 [0.02] mm; *P* < .001). However, there were no differences in SER and axial length change observed between the SAL and SVL groups during the second year (mean [SE] for SER, SAL: −0.57 [0.05] D; SVL: −0.64 [0.05 D]; *P* = .90; axial length, SAL: 0.27 [0.02] mm; SVL: 0.32 [0.02] mm; *P* = .12). Thus, compared with SVL, SAL slowed myopia progression of children mainly during the first year.

The mean daily wearing time throughout 2 years was similar among the groups, with mean (SE) durations of 13.4 (0.29) hours, 13.4 (0.24) hours, and 13.9 (0.24) hours for HAL, SAL, and SVL, respectively (*F*_2,154_ = 1.08; *P* = .34). The mean wearing hours increased throughout the study from 13.1 hours in the first year to 14.0 hours in the second year (95% CI, 0.47-1.17; *P* < .001), with 61% (n = 96) of children who were full-time wearers in the first year to 89% (n = 139) who were full-time wearers in the second year (*P* < .001). Therefore, we analyzed the myopia control efficacy based on the first-year full and part-time categories. The SER progression in the SAL and SVL groups was similar for full-time wearers and part-time wearers but was lower for full-time wearers in the HAL group (mean [SE] difference, 0.45 [0.16] D; *P* = .01; Table 2). Axial length elongation for full-time wearers was similar to that among part-time wearers in the SAL and SVL groups but was lower for full-time wearers in the HAL group (mean [SE] difference, 0.15 [0.07] mm; *P* = .03; Table 2). For full-time HAL wearers, the mean (SE) 2-year treatment effect compared with SVL group was 0.99 (0.12) D for SER and 0.41 (0.05) mm for axial length, whereas for HAL part-time wearers, it was 0.54 (0.15) D for SER and 0.26 (0.07) mm for axial length.

**Table 2.** Comparisons of Myopia Progression and Axial Elongation Between Full- and Part-time Wearers

Clinical value	Mean (SE)		
	HAL^a^	SAL^a^	SVL^a^
**Change in SER, D**			
Full-time wearers	−0.48 (0.10)	−0.95 (0.08)	−1.44 (0.10)
Part-time wearers	−0.93 (0.13)	−1.15 (0.10)	−1.50 (0.16)
*P* value	.01	.11	.77
**Change in AL, mm**			
Full-time wearers	0.28 (0.04)	0.46 (0.04)	0.69 (0.04)
Part-time wearers	0.43 (0.06)	0.57 (0.04)	0.70 (0.07)
*P* value	.03	.10	.92

Abbreviations: AL, axial length; HAL, spectacle lenses with highly aspherical lenslets; SAL, spectacle lenses with slightly aspherical lenslets; SER, spherical equivalent refraction; SVL, single-vision spectacle lenses.

^a^ Full-time wearers wore lenses at least 12 hours per day, and part-time wearers wore lenses less than 12 hours per day. In the HAL group, there were 32 full-time wearers and 22 part-time wearers. In the SAL group, there were 30 full-time wearers and 23 part-time wearers. In the SVL group, there were 34 full-time wearers and 16 part-time wearers.

### Adverse Events

None of the adverse events reported were severe or resulted in study discontinuation. Two events were mild and unrelated to the study device and resolved with no reported loss of best-corrected visual acuity. One SVL wearer had punctate corneal epithelial defects, and the other SAL wearer was hit by a ball, causing the spectacle frame to break and the participant’s face to be bruised. There was no significant difference in adverse events between treatment groups (*P* = .59).

## Discussion

In this 2-year randomized clinical trial, HAL slowed myopia progression by 0.80 D (55%) and increase in axial length by 0.35 mm (51%) compared with SVL. Compared with SAL, HAL slowed myopia progression by 0.38 D (37%) and axial length by 0.17 mm (33%). This outcome is well in line with the first-year interim analysis,^14^ and it confirms a positive dose-response relationship between myopia control efficacy and lenslet asphericity. The dose-dependent effect of optical interventions in minimizing lens-induced myopia in animal studies was attributed mainly to lens design features such as the amount and area of lens addition,^19,20^ peripheral defocus, and lens asphericity.^21,22^ This dose-dependent relationship was also found in human clinical studies for ophthalmic lenses with higher addition^10,12^ and contact lenses with higher asphericity.^23^ Compared with SVL, during the second year, HAL remained effective in slowing myopia progression. This was not the case for SAL that slowed myopia progression of children mainly during the first year. This is similar to the previous myopia control study using progressive addition lenses.^24^ It may be speculated that there is also a dose-response relationship between the duration of myopia control efficacy and lenslet asphericity. Therefore, it will be interesting to evaluate whether myopia control efficacy of HAL extends beyond 2 years.

Bifocal, prismatic bifocal spectacle lenses and defocus incorporated multiple segments (DIMS) spectacle lenses have also shown clinically significant myopia control results in Chinese children over the same duration as our study.^25,26^ Throughout 2 years, the myopia control efficacy for HAL (0.80 D) and prismatic bifocal lenses (0.85 D) were comparable, while SAL (0.42 D), bifocal lenses (0.59 D), and DIMS (0.44 D) had lower myopia control efficacy. In terms of reducing axial elongation, HAL (0.35 mm) and DIMS (0.34 mm) had higher efficacy than SAL (0.18 mm) and bifocal and prismatic bifocal lenses (0.21 mm). Differences in myopia control efficacy could be linked to differences in lens designs, namely, the concentric ring configuration with aspherical lenslets (this study), honeycomb configuration with spherical lenslets (DIMS),^27^ or increase in power over the lower part of the lens (bifocal and prismatic bifocal lenses). Another reason might be related to the control groups. Myopia progression rates among SVL wearers were −1.46 D in the present study and −1.55 D in the study by Cheng et al,^25^ which are consistent with previous findings in Chinese children with myopia^28,29^ while in the DIMS study, children wearing SVL progressed significantly less (−0.85 D).

The effect of wearing time on myopia control efficacy of HAL also indicated a similar dose-response relationship, increasing myopia control efficacy to 0.99 D (67%) and 0.41 mm (60%) for full-time wearers (at least 12 hours per day). A better myopia control effect with increased wearing time was also found in a study using defocus incorporated soft contact lenses.^13^ However, no correlation between lens wearing time and myopia control efficacy was found in the study using DIMS spectacle lenses.^26^ In the DIMS study, participants wore their study devices constantly for more than 15 hours per day.^26^ In contrast, 39% of participants in this study wore their spectacles for less than 12 hours every day in the first year and 11% in the second year. Throughout 2 years, myopia progression and axial elongation were significantly lower among full-time wearers than among part-time wearers in the HAL group but not in the SVL or SAL group. As such, full-time wearing of myopia control intervention should be recommended for better outcome.

### Strengths and Limitations

The first notable strength of this study is its low dropout rate of 6.5%. Second, the recruitment duration was short (approximately 2 months), with very punctual follow-ups (except the 18-month visit). This minimized the possible influence of confounding factors in the study, such as seasonal effects on myopia progression.^30^

Nevertheless, there were some limitations to this study. First, this study was conducted with a strictly Chinese cohort in Wenzhou, China. According to the myopia screening of school-aged children (7 to 18 years) from more than 1000 elementary and high schools of Wenzhou in 2020, the overall myopia prevalence and high myopia prevalence were 59.35% and 4.99%, respectively,^31^ which was comparable with the findings of 2 recent studies in China.^32,33^ The current study sample could represent school-aged children in China but does not necessarily represent children with myopia globally. Second, the precision of the questionnaire may not reflect actual wearing hours. An objective measure of wearing time would be preferred. Third, the 18-month visit was delayed by a mean of 3 weeks. As a result of COVID-19 causing an unprecedented global pandemic, a lockdown was imposed for 3 weeks in February 2020. Children did not physically attend school for 4 months from February to the end of May 2020.^34^ Other than the delay during the 18-month visit, COVID-19 did not have any major impact on the conduct of the study. The 24-month visit was conducted according to the investigation plan. Lastly, outdoor time was not measured and it may affect myopia progression in the study.

## Conclusions

In children with myopia, wearing HAL significantly reduced the rate of myopia progression and eye growth over 2 years compared with SAL and SVL. This study demonstrated a dose-dependent effect, with higher lenslet asphericity having greater myopia control efficacy. The full-time wearing of HAL increased myopia control efficacy to 0.99 D (67%) for SER and 0.41 mm (60%) for axial length.

## Notes

Supplement 1. Trial Protocol

Supplement 2. Standard Operating Procedures

Supplement 3. Statistical Analysis Plan

Supplement 4. Data Sharing Statement
