# Supplementary material for: Spectacle Lenses With Aspherical Lenslets for Myopia Control vs Single-Vision Spectacle Lenses: A Randomized Clinical Trial
Source: JAMA Ophthalmol. 2022 Mar 31;140(5):472–8. doi: 10.1001/jamaophthalmol.2022.0401 (PMC8972151; doi:10.1001/jamaophthalmol.2022.0401)
Supplement: Supplement 1. — Trial Protocol [file jamaophthalmol-e220401-s001.pdf]

COMPARATIVE STUDY

Myopia Control with Aspherical Lenslets

ChiCTR1800017683

STUDY PROTOCOL
Version 02.0

Date: April 5, 2019

A double-masked comparative study of HAL, SAL versus SVL

**Study design**	Monocenter, double-masked, randomized, controlled group study
**Test Lens**	HAL, SAL
**Reference Lens**	SVL
**Duration of study**	At least 2.5 years
**Study center**	Eye Hospital, Wenzhou Medical University<br>WEIRC<br>270 Xueyuan Road, Wenzhou, Zhejiang, China 325027
**Principal investigator & Protocol Writer**	BAO Jinhua<br>Eye Hospital, Wenzhou Medical University<br>WEIRC
**Funding**	International S&T Cooperation Program of China (2014DFA30940) Collaborative Research Project with Essilor International (Wenzhou Medical University grant numbers 95013006, 95016010 and 95020005).
**Study Manager**	JIN Wanqing (2018)<br>Eye Hospital, Wenzhou Medical University<br><br>LIM Ee Woon<br>Centre for Innovation & Technologies AMERA<br>Essilor R&D Centre Singapore

**Study Coordinator**	HUANG Yingying<br>Eye Hospital, Wenzhou Medical University, WEIRC	
**Study Examiner**	LI Xue	Eye Hospital, Wenzhou Medical University
	LI Shufeng	Eye Hospital, Wenzhou Medical University
	HUO Jiawen	Eye Hospital, Wenzhou Medical University
	PAN Yiguo	Eye Hospital, Wenzhou Medical University
	DING Chenglu	Eye Hospital, Wenzhou Medical University
	WANG Chu	Eye Hospital, Wenzhou Medical University
	LI Yuhao	Eye Hospital, Wenzhou Medical University
	ZHOU Fengchao	Eye Hospital, Wenzhou Medical University
	WU Junqian	Eye Hospital, Wenzhou Medical University

CONFIDENTIAL – © WEIRC 2018 – All rights reserved

Do not disclose, copy or distribute

Summary of Changes from Previous Version approved by IRB:

Affected Section(s)	Summary of Revisions Made	Rationale
2.3	Risk/Benefit Assessment	Inclusion for risk assessment
2.3.1	Known Potential Risks	Inclusion for risk assessment
2.3.2	Known Potential Benefits	Inclusion for risk assessment
2.3.3	Assessment of Potential Risks and Benefits	Inclusion for risk assessment
3	OBJECTIVES AND ENDPOINTS	Inclusion for clear endpoint
5.3	Screen Failures	Inclusion for clear description
5.4	Strategies for Recruitment and Retention	Inclusion for clear description
7	STUDY INTERVENTION DISCONTINUATION AND PARTICIPANT DISCONTINUATION/WITHDRAWAL	Inclusion for clear description
7.1	Discontinuation of Study Intervention	Inclusion for clear description
7.2	Participant Discontinuation/Withdrawal from the Study	Inclusion for clear description
7.3	Lost to Follow-Up	Inclusion for clear description
8.1	Efficacy Assessments	Inclusion for clear description
8.2	Safety and Other Assessments	Inclusion for clear description
8.3.4	Time Period and Frequency for Event Assessment and Follow-Up	Inclusion for clear timeline for reporting and responsibilities
8.3.5	Adverse Event Reporting	Inclusion for clear timeline for reporting and responsibilities
8.3.6	Serious Adverse Event Reporting	Inclusion for clear timeline for reporting and responsibilities
8.3.7	Reporting Events to Participants	Inclusion for clear timeline for reporting and responsibilities
8.4	Unanticipated Problems	Inclusion for clear timeline for reporting and responsibilities
8.4.1	Definition of Unanticipated Problems (UP)	Inclusion for clear timeline for reporting and responsibilities
8.4.2	Unanticipated Problem Reporting	Inclusion for clear timeline for reporting and responsibilities
9	STATISTICAL CONSIDERATIONS	Inclusion for clear description
9.1	Statistical Hypotheses	Inclusion for clear description
9.3	Populations for Analyses	Inclusion for clear description
9.4	Statistical Analyses	Inclusion for clear description
9.4.1	General Approach	Inclusion for clear description

CONFIDENTIAL – © WEIRC 2018 – All rights reserved

Do not disclose, copy or distribute

9.4.2	Analysis of the Primary Efficacy Endpoint(s)	Inclusion for clear description
9.4.3	Analysis of the Secondary Endpoint(s)	Inclusion for clear description
9.4.4	Safety Analyses	Inclusion for clear description
9.4.5	Baseline Descriptive Statistics	Inclusion for clear description
9.4.6	Planned interim analysis	Inclusion for clear description
9.4.7	Sub-Group Analyses	Inclusion for clear description
9.4.8	Tabulation of Individual participant Data	Inclusion for clear description
9.4.9	Exploratory Analyses	Inclusion for clear description
10.1.7	Clinical Monitoring	Inclusion for clear description
Appendix	Inclusion of SAP	Inclusion for clear description

CONFIDENTIAL – © WEIRC 2018 – All rights reserved

Do not disclose, copy or distribute

Table of Contents

1. TABLE OF CONTENTS
2. STATEMENT OF COMPLIANCE 1
1 PROTOCOL SUMMARY 1
1.1 Synopsis 1
1.2 Schema 2
1.3 Schedule of Activities (SoA) 2
2 INTRODUCTION 3
2.1 Study Rationale 3
2.2 Background 4
2.2.1 Prior Literature and Studies 4
2.2.1.1 Evaluation of optical strategies: 5
2.2.1.1.1 Under correction versus full correction spectacles 5
2.2.1.1.2 Multifocal Spectacles versus single vision spectacles 5
2.2.1.1.3 Myopic Defocus optical treatment 5
2.3 Risk/Benefit Assessment 6
2.3.1 Known Potential Risks 6
2.3.2 Known Potential Benefits 6
2.3.3 Assessment of Potential Risks and Benefits 7
3 OBJECTIVES AND ENDPOINTS 7
4 STUDY DESIGN 9
4.1 Overall Design 9
4.2 Scientific Rationale for Study Design 10
4.3 End of Study Definition 11
5 STUDY POPULATION 11
5.1 Inclusion Criteria 11
5.2 Exclusion Criteria 11
5.3 Screen Failures 12
5.4 Strategies for Recruitment and Retention 12
6 STUDY INTERVENTION 13
6.1 Study Intervention(s) Administration 13
6.1.1 Study Intervention Description 13
6.2 Preparation/Handling/Storage/Accountability 14
6.2.1 Acquisition and accountability 14
6.2.2 Formulation, Appearance, Packaging, and Labeling 15
6.2.3 Product Storage and Stability 15
6.2.4 Preparation 15
6.3 Measures to Minimize Bias: Randomization and Blinding 15
6.4 Study Intervention Compliance 16
6.5 Concomitant Therapy 16
7 STUDY INTERVENTION DISCONTINUATION AND PARTICIPANT DISCONTINUATION/WITHDRAWAL 16

CONFIDENTIAL – © WEIRC 2018 – All rights reserved

Do not disclose, copy or distribute

7.1 Discontinuation of Study Intervention 17
7.2 Participant Discontinuation/Withdrawal from the Study 17
7.3 Lost to Follow-Up 18
8 STUDY ASSESSMENTS AND PROCEDURES 18
8.1 Efficacy Assessments 18
8.2 Safety and Other Assessments 23
8.3 Adverse Events and Serious Adverse Events 23
8.3.1 Definition of Adverse Events (AE) 23
8.3.2 Definition of Serious Adverse Events (SAE) 23
8.3.3 Classification of an Adverse Event 24
8.3.3.1 Severity of Event 24
8.3.3.2 Relationship to Study INTERVENTION 24
8.3.3.3 Expectedness 25
8.3.4 Time Period and Frequency for Event Assessment and Follow-Up 25
8.3.5 Adverse Event Reporting 25
8.3.6 Serious Adverse Event Reporting 26
8.3.7 Reporting Events to Participants 26
8.4 Unanticipated Problems 26
8.4.3 Definition of Unanticipated Problems (UP) 26
8.4.4 Unanticipated Problem Reporting 27
9 STATISTICAL CONSIDERATIONS 27
9.3 Statistical Hypotheses 27
9.4 Sample Size Determination 28
9.5 Populations for Analyses 28
9.6 Statistical Analyses 29
9.6.3 General Approach 29
9.6.4 Analysis of the Primary Efficacy Endpoint(s) 29
9.6.5 Analysis of the Secondary Endpoint(s) 29
9.6.6 Safety Analyses 29
9.6.7 Baseline Descriptive Statistics 29
9.6.8 Planned interim analysis 30
9.6.9 Sub-Group Analyses 30
9.6.10 Tabulation of Individual participant Data 30
9.6.11 Exploratory Analyses 31
10 SUPPORTING DOCUMENTATION AND OPERATIONAL CONSIDERATIONS 32
10.3 Regulatory, Ethical, and Study Oversight Considerations 32
10.3.3 Informed Consent Process 32
10.3.3.1 Consent/assent and Other Informational Documents Provided to participants 33
1. 33
10.3.4 Subject confidentiality 33
10.3.4.1 Consent Procedures and Documentation 33
10.3.5 Study Discontinuation and Closure 34
10.3.6 Confidentiality and Privacy 34

10.3.7 Key Roles and Study Governance 35
○ 35
○ **Study center** 35
10.3.8 Safety Oversight 37
10.3.9 Clinical Monitoring 37
10.3.10 general Monitoring 38
10.3.11 Onsite Monitoring 38
10.3.12 Quality Assurance and Quality Control 39
10.3.13 Data Handling and Record Keeping 39
10.3.13.1 Data Collection and Management Responsibilities 39
10.3.13.2 Study Records Retention 40
10.3.14 Protocol Deviations 41
10.3.15 Publication and Data Sharing Policy 41
10.3.16 Conflict of Interest Policy 41
10.4 Appendices 42
10.5 Abbreviations 43

CONFIDENTIAL – © WEIRC 2018 – All rights reserved

Do not disclose, copy or distribute

2. STATEMENT OF COMPLIANCE

The trial will be conducted in accordance with the International Conference on Harmonisation Good Clinical Practice (ICH GCP). The Principal Investigator will assure that no deviation from, or changes to the protocol will take place without prior agreement from the sponsor, funding agency and documented approval from the Institutional Review Board (IRB), except where necessary to eliminate an immediate hazard(s) to the trial participants. All personnel involved in the conduct of this study have completed Human Subjects Protection and ICH GCP Training.

The protocol, informed consent form(s), recruitment materials, and all participant materials will be submitted to the IRB for review and approval. Approval of both the protocol and the consent form must be obtained before any participant is enrolled. Any amendment to the protocol will require review and approval by the IRB before the changes are implemented to the study. All changes to the consent form will be IRB approved; a determination will be made regarding whether a new consent needs to be obtained from participants who provided consent, using a previously approved consent form.

1 PROTOCOL SUMMARY

1.1 SYNOPSIS

**Title:**	Myopia Control with Aspherical Lenslets
**Study Description:**	*A monocenter, double-masked, randomized controlled group study to evaluate test lenses in comparison to single-vision spectacle lenses in slowing the progression of myopia.*<br><br>*A detailed schematic describing all visits and a schedule of assessments can be found in* ***Schema and Schedule of Activities, Sections 1.2 and 1.3,*** *respectively.*
**Objectives:**	Primary Objective: To evaluate the myopia control efficacy of the HAL and SAL lenses in slowing down the progression of myopia compared to single-vision spectacle lenses.
	Secondary Objectives: To compare the objective and subjective visual performance and subjective appreciation of the 2 test lenses compared to single-vision spectacle lenses.
**Endpoints:**	*Primary endpoint:*<br>• *33% improvement in spherical equivalent of cycloplegic autorefraction in at least one eye after 2 years compared to control arm OR 0.50D difference in myopia progression in at least one eye after 2 years compared to control arm using spherical equivalent of cycloplegic autorefraction or change in axial length converted to diopters using conversion of 0.10mm = 0.28D in at least one eye in one test arm compared to control arm.*<br>*Secondary endpoint:*

© WEIRC 2018 – All rights reserved

	• *80% or equivalent compliance and adaptation in the test arm compared to the control arm.*
**Study Population:**	*150 healthy myopic subjects aged 8 – 13 years old in China*
**Phase:**	N/A
**Description of Sites/Facilities Enrolling Participants:**	*This study will be done in WEIRC where the recruitment and examinations are done in the eye hospital in Wenzhou.*
**Description of Study Intervention:**	*The 2 test lenses are investigational devices made of polycarbonate. They are based on the optical defocus treatment theory where a volume of myopic defocus in front of the retina is used as a preventive signal to slow down eye elongation.*
**Study Duration:**	*At least 3 years*
**Participant Duration:**	*At least 2.5 years*

1.2 SCHEMA

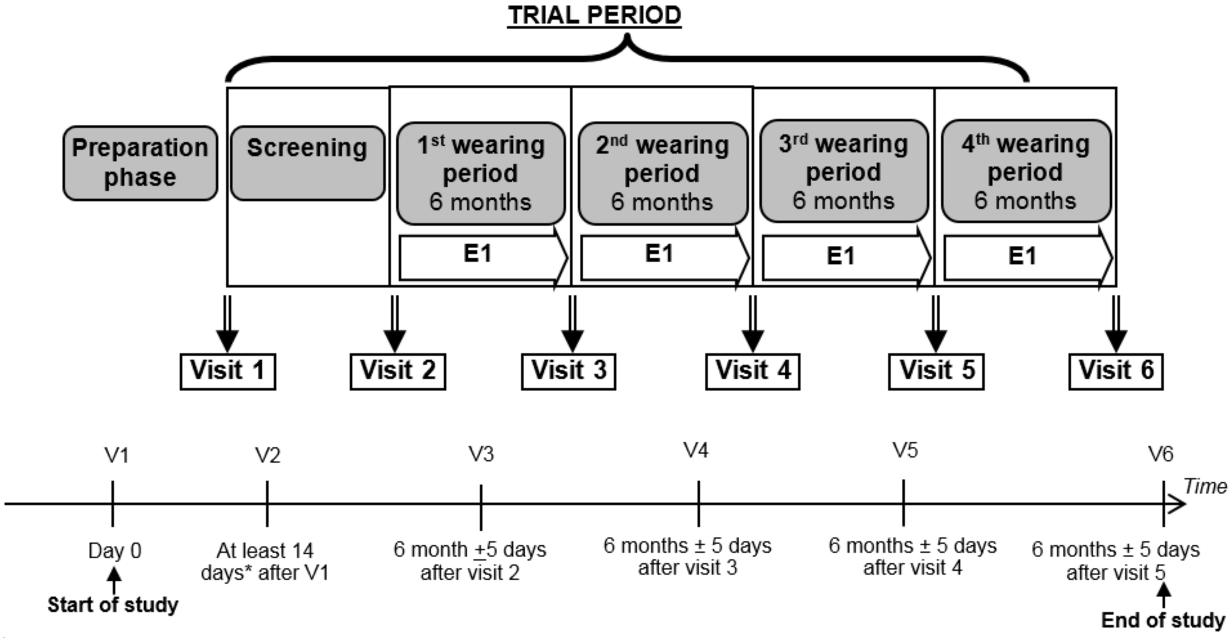

1.3 SCHEDULE OF ACTIVITIES (SOA)

Visit schedule	V1 (2hr)	V2	V3	V4	V5	V6

© WEIRC 2018 – All rights reserved

		(45min)	(1:25 hr)	(1:45 hr)	(1:25 hr)	(1:45 hr)
Informed Consent [5min]	X					
Eye examination (non cycloplegic) [30min]	X		X	X	X	X
Checking of inclusion / exclusion criteria	X					
Central axial length (Lenstar) [5min]	X		X	X	X	X
Peripheral axial length (Lenstar) [10min]	X					X
Cycloplegic subjective refraction [60min]	X		X	X	X	X
Cycloplegic Central autorefraction (Topcon) [10min]	X		X	X	X	X
Cycloplegic Peripheral autorefraction (Grand Seiko) [30min]	X					X
Choroidal Thickness (OCT) [10min]	X			X		X
Study frame choice [10min]	X		X	X	X	
Study frame adjustment [10min]	X		X	X	X	
Monocular pupillary distances and fitting measurements [5min]	X		X	X	X	
1st Equipment (E1) delivery		X				
Photopic & Mesopic Visual acuity measurements (Near & Distance 10%, 100% contrast) [15min]		X	X	X	X	X
Stereoacuity @40cm [5min]		X		X		X
Accommodation @33cm[T] (Grand Seiko) [10min]		X		X		X
Monocular and Binocular Amplitude of accommodation [5min]	X	X	X	X	X	X
Visual comfort questionnaire [10min]	X		X	X	X	X

The occurrence of delivery visits in between visits 2 to 6 are meant for subjects who need to change lenses or spectacle frames. Reasons for change include prescription change of 0.50D or more or had corrected visual acuity worse by 1 logMAR line compared to previous visit, or damages, scratches to lenses or broken or damaged frames or frame has become too small or the parents demand a change.

[T] *To use trial lenses.*

2 INTRODUCTION

2.1 STUDY RATIONALE

Myopia has been a significant public health issue that affects 41.5% of the population age 15-54 years old in the United States and the prevalence of myopia is much higher of up to 96.5% in Asia (Holden et

© WEIRC 2018 – All rights reserved

al., 2016 and Rudnicka et al., 2016). Children were not spared from the prevalence of myopia. Study in Australia has found that the prevalence of myopia in children was found to be 42.7% in East Asian schoolchildren and 8.3% for European Caucasian children at the age of 12 years old. Higher rates of myopia were found in other countries with higher prevalence rates in East Asian and Southeast Asian countries at 38.1% in Guangzhou, 36.7% in Hong Kong and 36.7% in Singapore. The progression of myopia from the onset of childhood often results in higher prevalence of myopia, severity and pathology with age (Fan et al., 2004, Ip et al., 2007, O'Donoghue et al., 2010, Quek et al., 2004, Wong et al., 2014).

With the increase in prevalence of myopia over the years, there will be significant impact on public health economically as well as morbidity as myopia has been known to increase the risk of retinal detachment, myopic retinopathy and glaucoma that can lead to severe visual impairment (Rudnicka et al., 2016, Saw, 2003, Wong et al., 2014). It was estimated that the annual direct cost of optical correction of myopia for Singaporean adults is at US$755 million. When this data was extrapolated to all cities in Asia, the estimated direct cost would be US$328 billion. This was just the estimate for Asian countries without the cost of healthcare for pathology related to myopia (Wong and Saw, 2016 and Zheng et al., 2013).

Currently, myopia is managed mainly by inducing hyperopic defocus by either using contact lenses like orthokeratology and multifocal soft contact lenses, and spectacles lenses like progressive lenses, bifocals and peripheral defocus lenses. Other methods like atropine are also used to control myopia. The above mentioned methods have various efficacy in controlling myopia with low dose atropine being the most effective at 59%, followed by orthokeratology with about 43% of myopia control and multifocal soft contact lenses at 49% of myopia control. These methods are invasive and may not be suitable for all the young children. Moreover, the rebound effect for atropine was not desirable upon cessation of drug use. For the spectacle correction method, bifocals have the best outcome of about 45% myopia control, however, there were aesthetical limitations.

It has been shown that a persistent myopic defocus constantly applied on the whole retina has a positive effect on myopia progression, i.e., decrease in myopia progression (Anstice and Phillips, 2011). Based on the above background and outcome of several studies on optical treatment on myopia progression we developed 2 test spectacle lenses as described in Section 6.1.1.

2.2 BACKGROUND

2.2.1 PRIOR LITERATURE AND STUDIES

There were several studies to develop effective optical treatment strategies for myopia and it has been reviewed that prismatic bifocals had the best outcome compared to other non-invasive optical options (Smith, 2013 and Chung 2010). With studies suggesting that myopic defocus applied on the whole retina may slow down the progression of myopia, optical designs have shifted towards imposing optical myopic defocus over a large part of the retina as opposed to a small part on the fovea (Chung, 2010 and

© WEIRC 2018 – All rights reserved

Ehsaei et al., 2011). The test lenses were calculated to generate a volume of myopic defocus in front of the retina at any eccentricity, serving as a myopia control signal.

With such impact on financial and morbidity, there were several studies that looked at interventions to reduce the progression of myopia. The optical treatments for slowing myopia progression are mainly bifocals, progressive additional lenses, contact lenses and orthokeratology. Of the above-mentioned intervention, peripheral defocus modifying contact lenses and prismatic bifocal spectacle lenses showed moderate effects and progressive addition spectacle lenses, bifocal spectacles lenses and peripheral defocus modifying spectacle lenses showed weak effects in myopia progression. Orthokeratology was the only intervention that improved axial length significantly compared to the other methods (Smith, 2013).

2.2.1.1 EVALUATION OF OPTICAL STRATEGIES:

2.2.1.1.1 UNDER CORRECTION VERSUS FULL CORRECTION SPECTACLES

Though animal studies imply that under correcting myopic eyes should slow myopia progression, this is not the case in Adler and Millodot, 2006 and Chung et al., 2002 studies). Both studies showed either that the under corrected group progressed significantly faster than the fully corrected group with associated axial length elongations rates or that there was a strong tendency towards faster myopia progression. It was suggested that the outcome of the animal experiments and human experiments differ due to the large amounts of relative hyperopia in the near periphery of myopic children and adults but not in animals [19]. As a result, only a small part of the retina experiences myopic defocus during distance fixation, hence it is not an effective treatment strategy.

2.2.1.1.2 MULTIFOCAL SPECTACLES VERSUS SINGLE VISION SPECTACLES

A review of several studies using multifocal spectacles reported the study by Cheng et al, 2010 to have the best outcome for myopia progression using prismatic bifocal lenses. It was thought that the near addition reduced accommodative lag in children. However, it was found that the effect was minimal and accommodative lag is not directly related to the rate of myopia progression (Berntsen et al., 2010, Cheng, 2010, Cheng et al., 2010). However, it is more plausible that the near addition produces myopic defocus in the peripheral retina when children are not doing near work. In agreement with this idea, it was found that PALs produced significant amounts of peripheral myopia in the superior retina and they reduced peripheral hyperopia along the horizontal meridian. In addition, the degree of peripheral myopic defocus measured in the superior retina was associated with slower myopia progression (Berntsen et al., 2013 and Bernstsen et al., 2010). In this case, executive bifocals have a greater treatment effect on myopia progression because the near segments cover a larger part of the visual field than the near add of D-segment bifocals and PALs (Cheng, 2010).

2.2.1.1.3 MYOPIC DEFOCUS OPTICAL TREATMENT

© WEIRC 2018 – All rights reserved

With studies to suggest that peripheral refractive errors may promote the development or progression of central myopia, optical designs emphasized on imposing optical effects over a large area of the retina as opposed to a small part on the fovea (Cheng, 2010 and Ehsaei et al., 2011). It is still unknown if peripheral hyperopia is causal in the genesis of axial myopia, as it has been observed prior to onset of central myopia in both children and adults (Mutti et al., 2000 and Hoogerheide et al., 1971), or peripheral hyperopia is a consequence of axial myopia (Guthrie, 2011, Liu and Wildsoet, 2012, Smith et al., 2009). Nevertheless, results from the above studies suggested that treatment regimens that take into account peripheral image quality are more likely to be successful in slowing myopia progression, at least for contact lenses. Further studies also confirmed that overall contribution of the fovea is small compared to the peripheral retina. It was hypothesized that the relative weight of signals from a given local area of the retina to overall central refractive development probably decreases with eccentricity with a counter balance by areal summation effects that increases with eccentricity. Knowledge of relative weighting functions and regional difference is still to be found for the development of optimal peripheral treatment (Wallman and Winawer, 2004). Nevertheless, it is an effective signal to control central refractive development without compromising central vision.

2.3 RISK/BENEFIT ASSESSMENT

2.3.1 KNOWN POTENTIAL RISKS

Study Examination:

- Hypersensitivity to cyclopentolate or any component of the preparation is rare but possible. The drug is contraindicated in patients with confirmed or suspected angle-closure in whom an acute attack may be precipitated. Associated visual disturbance due to photophobia and lack of accommodation will affect the ability to move about safely.

- Risks related to topical anesthesia include scratched cornea due to numbness and visual disturbances that can affect the ability of the child to move around safely. Hypersensitivity to topical anesthesia is also rare but possible, resulting in corneal melting.

Investigational Device:

- With about 50% of peripheral image in front of the retina, peripheral vision could be poorer compared to conventional SVL. There is a possibility that peripheral vision development gets affected by the test lenses after 2 years of study.

2.3.2 KNOWN POTENTIAL BENEFITS

It has been shown that a persistent myopic defocus constantly applied on the whole retina has a positive effect on myopia progression, i.e., decrease in myopia progression (Anstice and Phillips, 2011). Based on the above background and outcome of several studies on optical treatment on myopia progression we have developed 2 test spectacle lenses as described in Section 6.1.1.

© WEIRC 2018 – All rights reserved

2.3.3 ASSESSMENT OF POTENTIAL RISKS AND BENEFITS

The risk of study examination using topical anesthesia and cyclopentolate for the purpose of cycloplegia is equivalent to the risk of having any eye examination in a regular ophthalmic clinic. As in a conventional clinical setting, the risk will be communicated to the parent and child. In addition, prior tests to ensure that the anterior chamber angle is safe for cycloplegia are performed. Precautions like having sunglasses available for use after the procedure to ensure that dust does not enter the eye easily as well as to protect the eye from glare after cycloplegia are taken. Advice will be given to minimize contact with the eye to prevent the risk of rubbing and scratching of cornea. Should vision degrade within 45 minutes of cycloplegia, subjects should return to the hospital for further investigation and treatment.

Based on past and existing studies using contact lenses like multifocal contact lenses and spectacle lenses like progressive lenses and peripheral defocus lenses, there were no known risk of peripheral vision development even though all the above-mentioned interventions alter peripheral vision resulting in large amount of blur compared to the test lenses used in this study (Anstice and Phillip et al., 2011, Rui-Pomeda et al., 2018, Sankaridurg et al., 2010). Blur adaptation was found to be present in central and peripheral retina and there was no difference between emmetropes and myopes (Mankowska et al., 2012). Moreover, the above mentioned interventions had been used commonly for the purpose of myopia management with no known side effects on peripheral vision development.

To monitor for adverse changes in peripheral vision development, objective measures like peripheral autorefraction, peripheral axial length and choroidal thickness were performed yearly and compared with the control arm. In addition, visual acuity and contrast sensitivity with Freiburg Visual Acuity & Contrast Test (FrACT) while viewing through the peripheral zone of the lens did not show clinically significant differences between test lenses and SVL. As such, it is unlikely to have major effects on peripheral vision development.

With minimal risk in study examination and the effect of the investigational device on peripheral vision development, the benefits of myopia control that is potentially important in preventing vision impairment due to the increased risk in myopic pathology is much greater. As such the benefit of the investigational device in this study outweighs the minimal risk posed by the study.

3 OBJECTIVES AND ENDPOINTS

The study aims to evaluate the efficacy of test lenses to reduce the progression of myopia by either reducing the myopia progression rate per year and/or reducing the elongation of eyeball through myopic defocus compared with SVL group. 2 embodiments of the test lenses (HAL and SAL) will be tested and compared with single-vision spectacle lenses (SVLs) as a control. 150 healthy children aged from 8 to 13 years old will be recruited in a double masked randomized clinical trial over a wearing period of at least 2 years. Cycloplegic autorefraction and axial length will be the primary measure for

© WEIRC 2018 – All rights reserved

myopia progression. Other measures like peripheral autorefraction will be done to evaluate the effect of myopic defocus on peripheral retina. Finally, visual acuity and contrast sensitivity will also be compared between test lenses and SVLs to quantify the quality of vision using myopia control lenses like test.

OBJECTIVES	ENDPOINTS	JUSTIFICATION FOR ENDPOINTS
Primary		
The objective of the study is to evaluate the efficacy in controlling myopia progression assessed by measuring cycloplegic autorefraction and axial length of different versions of FIN lens designs compared to SVLs. This aim will be achieved by conducting a randomized clinical trial which will compare myopic progression in children wearing FIN lenses vs. children wearing SVLs. The comparison will allow the quantification of the effect of FIN lenses on myopia progression during the 2 year follow-up.	0.50D difference in myopia progression in at least one eye after two years compared to control arm using spherical equivalent of cycloplegic autorefraction (SER) AND change in axial length (AL) converted to diopters using conversion of 0.10mm = 0.28D in at least one eye and one test arm compared to control arm. Or 33% myopia control for AL and SER in two years.	Walline et al., 2018 agreed that 30-50% less progression in the test arm compared with control or reduction in progression of more than 0.75D difference over 3 years is a viable effectiveness threshold.
Secondary		
To evaluate the visual performance of FIN lenses compared to SVLs such as high and low contrast visual acuity under photopic and mesopic conditions. Moreover, this study aims to assess visual comfort using questionnaires with FIN lenses compared to SVLs.	80% or equivalent compliance and adaptation in at least one test arm compared to control arm.	Efficacy, safety and adherence of test arm achieving primary endpoint was based on previous studies on spectacle solution for myopia control with about 50% of test arm having an effective slower rate of progression in myopia and this test device is expected to perform better. 80% compliance and adaptation to a treatment is considered better than the average of 68% in most clinical trials (Czobor & Skolnick, 2011).
Exploratory		
To explore if at least one test arm could have an effect on peripheral cycloplegic autorefraction and axial length in one eye, and/or have an	• To have less negative peripheral spherical equivalent of cycloplegic autorefraction at nasal 30°	Radhakrishnan et al. 2013 found 0.24D change in peripheral refraction at nasal 30° over the course of years for test and control group

© WEIRC 2018 – All rights reserved

effect on choroidal thickness in one eye when compared to control arm.	in at least one eye in one test arm.<br>• And/or choroidal thickness change should be the same or less changes compared to control arm having more significant thinning in at least one eye in one test arm.	with myopia progression. Having less than 0.24D change in peripheral refraction will show positive effect of test arm on peripheral retina. Similarly, statistical significantly less choroidal thinning compared to control group is a positive effect of test arm.

4 STUDY DESIGN

4.1 OVERALL DESIGN

This is a Phase 3 monocenter, randomized, double masked controlled-group study to evaluate if FIN design lenses can slow down the progression of myopia through superiority testing. 150 healthy children age between 8 years to 13 years old with myopia of -0.75D to -4.75D (spherical equivalent) and meeting the specific inclusion and exclusion criteria will be recruited. Consent will be taken from guardians and children who are eligible for the study and they will be randomized to wear either of the 2 test lens design (test arm), single-vision spectacle lenses (control arm). All children will be followed up 6 monthly for 2 years to monitor for changes in cycloplegic autorefraction and axial length. At the end of the study, the above measures will be compared between test and control arm for objective and subjective performance. Figure 1 shows the timeline of the study.

Each subject will be followed for a duration of at least 2 years and 3 weeks.

The ophthalmic lenses will be worn by subjects and allocated according to a randomization ratio of 1:1:1 in each arm (150/3 in each arm – please see definition of arm page 4).

The study will be conducted at the Eye Hospital (WEIRC), Wenzhou Medical University, Wenzhou city, Zhejiang province, China in accordance with the regulations and hospital IRB policies that follow the Tenets of the Declarations of Helsinki.

Figure 1. Study diagram

© WEIRC 2018 – All rights reserved

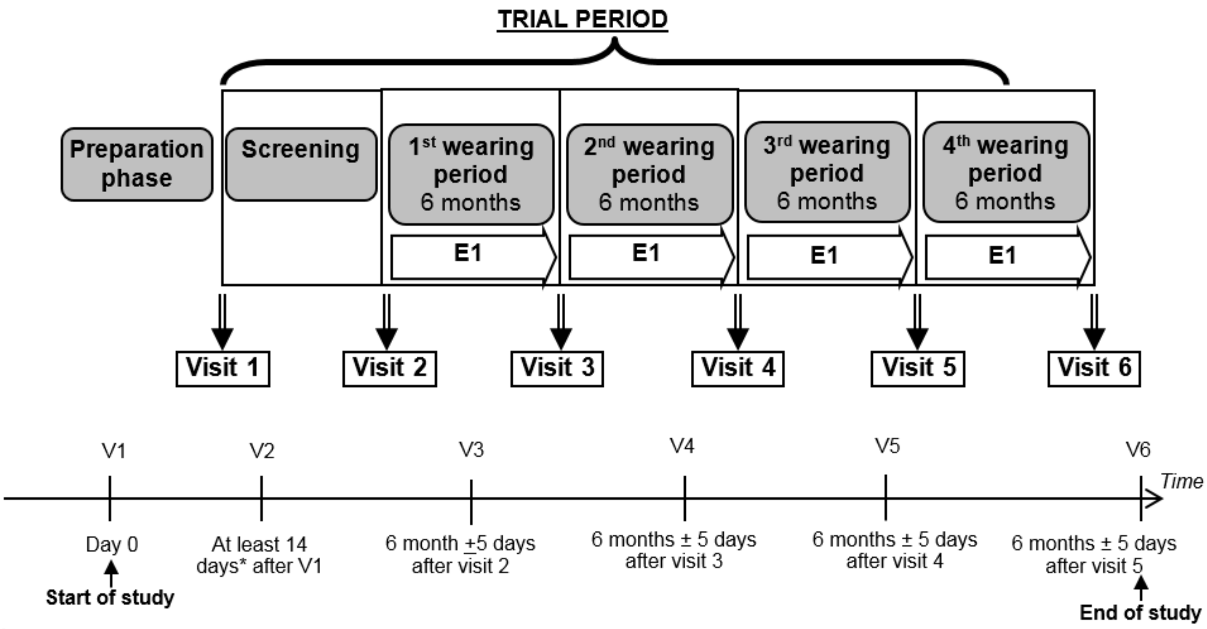

Superiority:

- Hypotheses: SAL or HAL > SVL, P-value 0.05
- Superiority bound(s):
  - 95% confidence interval for upper and lower bounds at 5% level of significance for:
    - Cycloplegic spherical equivalent
    - Axial length
- Population: Intention-to-Treat

- *Detail of Statistical Analysis Plan in Appendix 1.*

4.2 SCIENTIFIC RATIONALE FOR STUDY DESIGN

This is a study where the assessment and comparison of lenses for myopia progression control is mainly based on objective measures like cycloplegic autorefraction and axial length. The double-masked randomized design remains the preferred approach for this type of study to reduce placebo effect and examiner bias.

This study will allow the evaluation and comparison of myopia control lenses performance in clinical and real-life conditions.

The performance assessment is based on clinical examinations and questionnaire.

© WEIRC 2018 – All rights reserved

The trial period duration of Test and Reference equipment, 2 years and 3 weeks, is based on the requested time for assessment of lens performance in several activities met by subjects and the duration to observe the progression of myopia.

4.3 END OF STUDY DEFINITION

A subject is considered to have completed the study if he or she has completed all phases of the study including the last visit or the last scheduled procedure shown in the Schedule of Activities (SoA), Section 1.3.

The end of the study is defined as completion of the last visit or procedure shown in the SoA in the trial globally.

5 STUDY POPULATION

5.1 INCLUSION CRITERIA

Subjects will be included in the study if they satisfy the following criteria:

General inclusion criterion:

- Volunteer subject and guardian, fluent Chinese spoken, willing to follow the protocol and able to read, comprehend and sign the informed consent form.

Study related inclusion criteria:

- Age: equal to or greater than 8 years and not older than 13 years.
- Spherical refractive error of -0.75 to -4.75 D in each eye (spherical equivalent), as measured by cycloplegic autorefraction.
- Astigmatism of not more than 1.50 D.
- Anisometropia of not more than 1.00 D.
- Best corrected visual acuity of equal or better than 0.05 LogMAR (≥ 0.9 as Snellen)
- No strabismus by cover test at near and distance.
- Have the ability to comply with the protocol to get reliable study measurements.
- Absence of ocular disease with full ophthalmic examination, such as retinal disease, cataract and ptosis. Good general health, without systemic or neurodevelopmental conditions. Without ocular or systemic medicine, which might affect myopia progression or visual acuity through known effects on retina, accommodation or significant elevation of intraocular pressure.
- No history of PALs or bifocal use and no prior use of contact lenses, or any treatment for myopia control.

5.2 EXCLUSION CRITERIA

Subjects presenting with any of the following exclusion criteria will not be included in the study:

© WEIRC 2018 – All rights reserved

General exclusion criteria:

- Vulnerability of the subject,
- Participation in another study which might have an influence on vision or interfere with study assessments,

Study related exclusion criteria:

- Age: less than 8 years old or greater than 13 years.
- Spherical refractive error: less than -0.75 D or greater than -4.75 D in each eye (spherical equivalent), as measured by cycloplegic autorefraction.
- Astigmatism of more than 1.50 D.
- Anisometropia of more than 1.00 D.
- Best corrected visual acuity of less than 0.05 LogMAR (≤ 0.8 as Snellen)
- With strabismus by cover test at near or distance.
- Without the ability to comply with the protocol to get the reliable study measurements
- Presence of any ocular disease that would influence refractive development, such as retinal disease, cataract and ptosis. Presence of systemic or neurodevelopmental conditions that may influence refractive development. Use of ocular or systemic medicine, which might affect myopia progression or visual acuity through known effects on retina, accommodation or significant elevation of intraocular pressure.
- Prior use of progressive adaptive lenses, bifocal use, use of contact lenses, or any treatment for myopia control.

5.3 SCREEN FAILURES

Screen failures are defined as subjects who consent to participate in this study but are not subsequently randomly assigned to the study intervention or entered in the study. Screen failure information may be found in the CRF under the screening form. The minimal information includes demography, screen failure details, eligibility criteria, and any serious adverse event (SAE).

Individuals who do not meet the criteria for participation in this trial (screen failure) because of cycloplegic autorefraction, may be rescreened. Rescreened participants should be assigned the same participant number as for the initial screening.

5.4 STRATEGIES FOR RECRUITMENT AND RETENTION

150 healthy children (50 per group) aged between 8 years to 13 years old with myopia of -0.75D to -4.75D (spherical equivalent) and meeting the specific inclusion and exclusion criteria will be recruited. These children will be recruited in WEIRC in China during the holiday season as they have more than 600 pediatric patients a day. The targeted population are from the general public who bring their child to the hospital for general eye examination. When a child is found to be suitable for this study, the ophthalmologist will recommend the child to the clinical trial coordinator for this study. The study coordinator will explain to the parents and child about the study and invite them to join and get consent from them.

© WEIRC 2018 – All rights reserved

Recruitment is also done through word of mouth by parents with children in the study with positive reviews about it. In addition, the recruitment poster is also circulated through social media, WeChat. General information about the study will be stated and more information can be found upon contacting the study team.

Due to the strict inclusion criteria and requirement of long-term participation with numerous follow up, an initial screening through the phone is usually done prior to the actual recruitment. To help with retention, a gift is given to the child during each visit. In addition, reminders are given before each appointment and subjects are constantly in contact with the study team so that they are free to notify the study team should they have any concern or issue. To ensure that all study equipment can be worn comfortably, a 3 days post dispensing phone call will be made to check on the child and another call 3 months later to check on equipment compliance and to address any issue that may occur during the wearing period. Finally, study subjects get free eye examination and after the end of the study, they have a pair of spectacles with single vision lenses or myopia control lenses from the sponsor depending on their choices.

All eligible children will be given informed consent together with their parents. The child assent or agreement will be obtained before the study. Should the child refuse to provide assent, this decision will not be nullified by the consent provided by legal parents/guardians. In addition, informed consent is written in their native language, Chinese. During the study, declination to participate by the child will be abided by the investigator.

6 STUDY INTERVENTION

6.1 STUDY INTERVENTION(S) ADMINISTRATION

6.1.1 STUDY INTERVENTION DESCRIPTION

Frames

Frame features must be the following:

- Rimless frames are excluded,
- Minimum frame height (B) of 30 mm,

The subjects will be invited to choose a frame which will be fitted with either Test lenses or Control lenses.

Ophthalmic Lens

Each subject included will wear 1 pair of ophthalmic lenses during the study:

- One pair of **Test lenses** HAL or SAL.

  OR
- One pair of **Reference lenses** SVL

For each subject, all the three pairs of lenses have a similar material, coating and tint. The only difference is the optical design.

© WEIRC 2018 – All rights reserved

SAL:

- Invisible lenslet ring structure (slightly aspherical lenslets)
- Alternating rings of distance correction and slightly aspherical lenslets for myopia control.
- The geometry of the lenslets of each defocus ring varies from center to the periphery of the lens.

HAL:

- Invisible lenslet ring structure (highly aspherical lenslets)
- Alternating rings of distance correction and highly aspherical lenslets for myopic control.
- The geometry of the lenslets of each defocus ring varies from center to the periphery of the lens.

The 2 test lenses are based on the optical defocus treatment theory (see a description in the "Introduction and study rationale" paragraph).
The basic concept of these FIN lenses is to bring, at the same time, on the retina:

- The distance correction which will be used by the user to perform everyday tasks,
- A volume of myopic defocus in front of the retina used as a preventive signal to slow down eye elongation.

The 3 pairs of ophthalmic lenses used for the study are described in table 2 below.

Characteristics of ophthalmic lenses

Lens	Material	Coating	Back Surface	Front Surface
SVL	Polycarbonate	Crizal Forte UV	Single Vision	Single Vision
SAL	Polycarbonate	Crizal Forte UV	Single Vision	Invisible lenslet ring structure (slightly aspherical)
HAL	Polycarbonate	Crizal Forte UV	Single Vision	Invisible lenslet ring structure (highly aspherical)

SVL is commercially available and it is a product of Essilor known as Airwear. Test lenses HAL and SAL are fabricated in Essilor and have Certificate of Conformity in compliance to ISO and CE standard to ensure safety standards of lenses are met.

6.2 PREPARATION/HANDLING/STORAGE/ACCOUNTABILITY

6.2.1 ACQUISITION AND ACCOUNTABILITY

At each visit, Investigator must ensure that the subject brings back the equipment.
In cases of equipment or lenses lost, the Investigator will interview the subject on the circumstances of loss. The Investigator will ask the subject to sign an attestation of no restitution and record the information in the Case Report Form.

The presence of lenses and/or equipment will be checked by the Study Coordinator during monitoring visit(s) and after the study.

In case of recall of lenses (decided by ESSILOR R&D) during the study, the Investigator will be immediately informed by the Study Manager (ESSILOR R&D).

© WEIRC 2018 – All rights reserved

The Investigator, in collaboration with the Study Manager (ESSILOR R&D) must urgently:

- Stop the delivery of the concerned equipment to the subjects,
- Inform the concerned subjects that they must immediately stop wearing the equipment and bring them back.

The Study Manager (ESSILOR R&D) will organize the return of the recalled lenses to ESSILOR R&D, according to ESSILOR R&D procedures.

6.2.2 FORMULATION, APPEARANCE, PACKAGING, AND LABELING

Essilor will be manufacturing the test and control lenses in accordance with ISO standard. The packaging will only include prescription, lens material and Subject ID. After edging and mounting, the spectacles will be in a usual spectacle box with Subject ID ready for dispensing.

6.2.3 PRODUCT STORAGE AND STABILITY

After reception from the person in charge of fabrication, equipment will be maintained under the Investigator's care and responsibility, depending on the study center procedures. The Investigator will be asked to keep the equipment in an appropriate secure area. When equipment is delivered to a subject, the Investigator will also provide instructions to the subject in order to keep appropriate use conditions.

Throughout the study, all unused lenses, broached or empty packaging, should be retained in a secure place by the Investigator. When the study is completed, all used and unused lenses will be returned to ESSILOR R&D for storage except the complementary pair (given to the subject as a compensation for his/her participation in the study).

6.2.4 PREPARATION

Before delivery, lenses will be verified by ESSILOR R&D using the focimeter with the prescription and pupillary distance. At the delivery of each equipment to the subject, the pupillary distance must be checked and the frame must be adjusted at the Eye Hospital.

6.3 MEASURES TO MINIMIZE BIAS: RANDOMIZATION AND BLINDING

A scheduled randomization will be generated by Study Manager (ESSILOR R&D) using online application Randola and forwarded **only** to the person in charge of fabrication in France or Singapore. The Investigators will never have access to the randomization list. Randomization is based on the spherical equivalent of cycloplegic autorefraction in the right eye, age and gender.

Each time a subject becomes eligible for the study, he/she will be randomly assigned by the Study Manager (ESSILOR R&D) to an arm in a ratio 1:1:1, then the Investigator will receive from the person in charge of fabrication accordingly.

© WEIRC 2018 – All rights reserved

Once the database has been locked and populations for analysis agreed, all codes (subject identification and randomization) will be re-checked, accounted for and provided to ESSILOR R&D.

The Investigator, subjects, and study personnel of the study center will be masked to the trial equipment worn.

The pockets containing the lenses will be the same for both Reference and Test lenses. The lens packaging design will be created in order to dispense the correct equipment to each subject according to the randomization list.

In the event of early discontinuation of the study or in case of emergency, the Investigator will ask the Study Manager (ESSILOR R&D) to break the masking, but only if it is considered mandatory for further major reason. The fact that a subject reaches an endpoint is not a reason for breaking the masking.

The Investigator should record the reason for, the date and time of breaking, his/her name and function in the study together with his/her signature. The date and reason for the unmasking must also be documented in the source document and the Case Report Form.

6.4 STUDY INTERVENTION COMPLIANCE

Compliance with intervention is monitored using questionnaires collected during Visit 3, 4, 5 and 6. In addition, phone interviews will be done 3 days, 2 weeks and 3 months post dispensing after every visit. The main measure of compliance is the number of days the investigative device was worn and the number of hours worn for each day. **Total compliance** is defined as 7 hours a day for 7 days a week, which is equivalent to 49 hours of wearing a week. Based on total hours of wearing in a week, **Actual compliance** will be actual wearing duration in a week and **Individual compliance** is defined below:

Individual compliance = (Actual compliance / Total compliance) * 100%

Study compliance will be the percentage of study participants that manage to comply at least 80% of Individual compliance, defined below:

Study compliance = (Individual compliance => 80% / Total study population) * 100%

6.5 CONCOMITANT THERAPY

For this protocol, a prescription medication is defined as a medication that can be prescribed only by a properly authorized/licensed clinician. Medications to be reported in the Case Report Form (CRF) are concomitant prescription medications, over-the-counter medications and supplements that may or may not affect myopia.

7 STUDY INTERVENTION DISCONTINUATION AND PARTICIPANT DISCONTINUATION/WITHDRAWAL

© WEIRC 2018 – All rights reserved

7.1 DISCONTINUATION OF STUDY INTERVENTION

Discontinuation from study equipment does not mean discontinuation from the study, and remaining study procedures should be completed as indicated by the study protocol. If a clinically significant finding is identified (including, but not limited to changes from baseline) after enrollment, the investigator or qualified designee will determine if any change in participant management is needed. Any new clinically relevant finding will be reported as an adverse event (AE).

The data to be collected at the time of study intervention discontinuation will include the following:

- Subjective refraction
- Central and Peripheral Cycloplegic autorefraction
- Central and Peripheral Axial length measure
- Choroidal thickness

7.2 PARTICIPANT DISCONTINUATION/WITHDRAWAL FROM THE STUDY

A subject who does not meet eligibility criteria is automatically withdrawn from the study. The reason for non-inclusion is documented in the appropriate section of visit 1 in the Case Report Form (the Early Discontinuation Form in the Case Report Form is not completed in this case).

After inclusion into the study (following screening visit), the reasons for a subject's premature withdrawal may be the following:

- The subject's decision. A subject who wishes to withdraw from the study for any reason may do so at any time but must inform the Investigator.
- Significant study intervention non-compliance
- If any clinical adverse event (AE), laboratory abnormality, or other medical condition or situation occurs such that continued participation in the study would not be in the best interest of the participant
- Disease progression which requires discontinuation of the study intervention
- If the participant meets an exclusion criterion (either newly developed or not previously recognized) that precludes further study participation

In all cases (except in case of definitely lost to follow-up subject), the Investigator must see the subject as soon as possible for a final assessment in order to:

- Obtain the reason(s) for withdrawal
- Know whether he wants to stop the study with or without consent withdrawal
- If necessary, take appropriate decision: management of an adverse event/effect or concomitant disease known after randomization.

All this information must be filled in the "Early discontinuation" Form in the Case Report Form.

The withdrawal will be forwarded to the Study Manager as soon as possible. Subjects who sign the informed consent form and are randomized but do not receive the study intervention may be replaced.

© WEIRC 2018 – All rights reserved

Subjects who sign the informed consent form, and are randomized and receive the study intervention, and subsequently withdraw, or are withdrawn or discontinued from the study, will not be replaced.

7.3 LOST TO FOLLOW-UP

A subject will be considered lost to follow-up if he or she fails to return for 2 consecutive scheduled visits and is unable to be contacted by the study site staff.

The following actions must be taken if a subject fails to return to the clinic for a required study visit:

- The site will attempt to contact the participant and reschedule the missed visit within 2 weeks of actual visit and counsel the participant on the importance of maintaining the assigned visit schedule and ascertain if the participant wishes to and/or should continue in the study.
- Before a participant is deemed lost to follow-up, the investigator or designee will make every effort to regain contact with the participant (where possible, 3 telephone calls and, if necessary, a certified letter to the participant's last known mailing address or local equivalent methods). These contact attempts should be documented in the participant's medical record or study file.
- Should the participant continue to be unreachable, he or she will be considered to have withdrawn from the study with a primary reason of lost to follow-up.

8 STUDY ASSESSMENTS AND PROCEDURES

8.1 EFFICACY ASSESSMENTS

Variables	Endpoints
Primary	
Central axial length (Lenstar)	0.27mm difference from control, P < 0.05, 2-tailed
Cycloplegic Central autorefraction (Topcon)	0.50D difference from control, P < 0.05, 2-tailed
Secondary	
Visual comfort questionnaire	80% compliance
Photopic & Mesopic Visual acuity measurements (Near & Distance 10%, 100% contrast)	No difference from control, P > 0.05, 2-tailed
Average wearing time	No difference from control, P > 0.05, 2-tailed
Exploratory	
Choroidal Thickness (OCT)	No change or better than control

© WEIRC 2018 – All rights reserved

Cycloplegic Peripheral autorefraction (Grand Seiko)	less negative than control
Peripheral axial length (Lenstar)	less negative than control

Screening Assessments
History taking – Demographics (age, gender, myopia onset, parental myopia), wearing habits, general health and medication, general eye examination to rule out ocular disorder (ocular pathology, binocular vision issue), subjective and autorefraction to estimate possibility of inclusion.

Visit 1: Screening & Baseline

- Study explanation (reading of information sheet of the Informed Consent with the subject)
- Agreement for subject participation in the study (signing of the Informed Consent Form)
- Eye examination:
    - Subject's demography: gender, date of birth
    - Parents' demography: myopia status, occupation, education level
    - Case history: general health, ocular health and history, medication
    - Current glasses description: date of prescription, wearing time per day and week, prescription, visual acuities, fitting measurements, lenses description
    - Preliminary examinations: complaint screening
    - Binocular vision measurements: suppression screening, dissociated phoria, strabismus screening
    - New prescription: auto-refractometer measurement, refraction, visual acuities
        - Study frame choice
        - Study frame adjustment
        - Monocular pupillary distances and fitting measurements
- Checking of inclusion / exclusion criteria
- Investigator decision to include the subject into the study
- Baseline examination
    - Visual comfort questionnaire
    - Monocular amplitude of accommodation
    - Choroidal thickness
    - Axial length measure (central and peripheral)
    - Anterior chamber depth, vitreous chamber depth, corneal thickness & lens thickness
    - Cycloplegic autorefraction (central and peripheral)

After the screening visit, the Study Manager (WEIRC) will check all the eligibility criteria and will be in charge of the definitive inclusion of the subject into the study. The randomization (see definition page 4) will be done by the Study Manager (ESSILOR R&D) after definitive inclusion.

The results of the eye examination and other information must be present in source documents at the study center.

© WEIRC 2018 – All rights reserved

Any significant findings discovered, after the signing of the Informed Consent Form, must be included and documented in the corresponding section of the Case Report Form (Adverse Event form).

Efficacy Assessments
Primary endpoint: Cycloplegic autorefraction, axial length measure
Secondary endpoint: Phone interview post dispensing, questionnaire every follow-up (patient reported outcome)
Exploratory endpoint: Peripheral cycloplegic autorefraction & axial length measure, choroidal thickness

- Visit 2: Equipment (E1) delivery
At least 2 weeks ± 3 days after visit 1

- Documentation of Adverse Events*
- Equipment (E1) delivery
    - Check frame adjustment
    - Check lens fitting
- Test with Equipment (E1)
    - Photopic & Mesopic visual acuity measurements 10%, 100%, distance and near
    - Stereoacuity
    - Accommodation at 33cm[T]
    - Monocular amplitude of accommodation

Between visit 2 and visit 3, recommendation for the subject is to wear E1 for more than 6 hours per day, every day of the week.

** The occurrence of Adverse Events (AEs) since the last visit will be determined by the subject's spontaneous reporting, the Investigator's non-leading questioning and his/her evaluation. All AEs will be reported in the Case Report Form (See section 8.3 Adverse Events).*

- Visit 3: First follow-up (6 months)
At least 6 months ± 5 days *after visit 2*

- Documentation of adverse events*
- Test with Equipment (E1):
    - Visual comfort questionnaire
    - Photopic & Mesopic Visual acuity measurements 10%, 100%, distance and near
- Subjective Refraction
- Axial length measure
- Anterior chamber depth, vitreous chamber depth, corneal thickness & lens thickness
- Cycloplegic autorefraction
- Choroidal Thickness

For all visits, recommendation for the subject is to wear E1 more than 6 hours per day, every day of the week. May need to arrange a visit 2 weeks later should there be a need to change the test lenses to a new prescription.

© WEIRC 2018 – All rights reserved

- Visit 3[B]: Equipment delivery[B] after Visit 3
At least 2 weeks ± 3 days after visit 3

- Documentation of Adverse Events*
- Equipment (E1) delivery:
  - Check frame adjustment
  - Check lens fitting
- Test with Equipment (E1)
  - Photopic & Mesopic visual acuity measurements 10%, 100%, distance and near
  - Stereoacuity
  - Monocular amplitude of accommodation

Between visit 3 and visit 4, recommendation for the subject is to wear E1 more than 6 hours per day, every day of the week.

- Visit 4: Second follow-up (12 months)
At least 6 months ± 5 days *after visit 3*

- Documentation of adverse events*
- Test with Equipment (E1)
  - Visual comfort questionnaire
  - Photopic & Mesopic Visual acuity measurements 10%, 100%, distance and near
  - Stereoacuity
  - Accommodation at 33cm[T]
  - Monocular amplitude of accommodation
- Subjective Refraction
- Choroidal thickness
- Axial length measure
- Anterior chamber depth, vitreous chamber depth, corneal thickness & lens thickness
- Cycloplegic autorefraction

- Visit 4[B]: Equipment delivery[B] after Visit 4
At least 2 weeks ± 3 days after visit 4

- Documentation of Adverse Events*
- Equipment (E1) delivery:
  - Check frame adjustment
  - Check lens fitting
- Test with Equipment (E1):
  - Photopic & Mesopic visual acuity measurements 10%, 100%, distance and near
  - Stereoacuity
  - Monocular amplitude of accommodation

Between visit 4 and visit 5, recommendation for the subject is to wear E1 more than 6 hours per day, every day of the week.

© WEIRC 2018 – All rights reserved

- Visit 5: Third follow-up (18 months)
At least 6 months ± 5 days *after visit 4*

- Documentation of adverse events*
- Test with Equipment (E1)
    - Visual comfort questionnaire
    - Photopic & Mesopic Visual acuity measurements 10%, 100%, distance and near
- Subjective Refraction
- Axial length measure
- Anterior chamber depth, vitreous chamber depth, corneal thickness & lens thickness
- Cycloplegic autorefraction

Visit 5[B]: Equipment delivery[B] after Visit 5
At least 2 weeks ± 3 days after visit 5

- Documentation of Adverse Events*
- Equipment (E1) delivery
    - Check frame adjustment
    - Check lens fitting
- Test with Equipment (E1)
    - Photopic & Mesopic visual acuity measurements 10%, 100%, distance and near
    - Stereoacuity
    - Monocular amplitude of accommodation

Between visit 5 and visit 6, recommendation for the subject is to wear E1 more than 6 hours per day, every day of the week.

- Visit 6: Equipment evaluation / Final follow-up
At least 6 months ± 5 days *after visit 4*

- Documentation of adverse events*
- Test with Equipment (E1)
    - Visual comfort questionnaire
    - Photopic & Mesopic Visual acuity measurements 10%, 100%, distance and near
    - Stereoacuity
    - Accommodation at 33cm[T]
    - Monocular amplitude of accommodation

- Subjective Refraction
- Choroidal thickness
- Axial length measure (central and peripheral)
- Anterior chamber depth, vitreous chamber depth, corneal thickness & lens thickness
- Cycloplegic autorefraction (central and peripheral)

© WEIRC 2018 – All rights reserved

** The occurrence of Adverse Events (AEs) since the last visit will be determined by the subject's spontaneous reporting, the Investigator's non-leading questioning and his/her evaluation. All AEs will be reported in the Case Report Form (See section 8.3 Adverse Events).*

8.2 SAFETY AND OTHER ASSESSMENTS

During all visits, ocular health of subjects will be monitored and any adverse outcome will be recorded and treated accordingly.

8.3 ADVERSE EVENTS AND SERIOUS ADVERSE EVENTS

Definition for **Adverse Events (AEs) and Serious Adverse Events (SAEs)** are presented in this chapter. It is of utmost importance that all people participating in the study understand these definitions and procedures. It is the Investigator's responsibility to ensure that this knowledge is acquired and reported to the Study Manager (ESSILOR R&D).

8.3.1 DEFINITION OF ADVERSE EVENTS (AE)

An **Adverse Event** is any untoward medical occurrence, unintended disease or injury or any untoward clinical signs (including an abnormal laboratory finding) in subjects, users or other persons whether or not related to the investigational medical device.

NOTE 1: This includes events related to Test and Reference lenses.

NOTE 2: This includes events related to the procedures involved (any procedure in the investigation plan).

NOTE 3: For users or other persons this is restricted to events related to the investigational medical device.

8.3.2 DEFINITION OF SERIOUS ADVERSE EVENTS (SAE)

A **Serious Adverse Event** is Adverse Event that:

- Led to a death,
- Led to a serious deterioration in health that either:
- Resulted in a life-threatening illness or injury, or
- Resulted in a permanent impairment of a body structure or a body function, or
- Required in-patient hospitalization or prolongation of existing hospitalization, or
- Resulted in medical or surgical intervention to prevent life threatening illness or injury or permanent impairment to a body structure or a body function.
- Led to fetal distress, fetal death or a congenital abnormality or birth defect.

© WEIRC 2018 – All rights reserved

NOTE 1: This includes device deficiencies that might have led to a serious adverse event if a) suitable action had not been taken or b) intervention had not been made or c) if circumstances had been less fortunate. These are handled under the SAE reporting system.

NOTE 2: A planned hospitalization for pre-existing condition, without a serious deterioration in health, is not considered to be a Serious Adverse Event.

8.3.3 CLASSIFICATION OF AN ADVERSE EVENT

8.3.3.1 SEVERITY OF EVENT

For adverse events (AEs) not included in the protocol defined grading system, the following guidelines will be used to describe severity.

- **Mild** – Events require minimal or no treatment and do not interfere with the participant’s daily activities.
- **Moderate** – Events result in a low level of inconvenience or concern with the therapeutic measures. Moderate events may cause some interference with functioning.
- **Severe** – Events interrupt a participant’s usual daily activity and may require systemic drug therapy or other treatment. Severe events are usually potentially life-threatening or incapacitating. Of note, the term “severe” does not necessarily equate to “serious”.

8.3.3.2 RELATIONSHIP TO STUDY INTERVENTION

All adverse events (AEs) must have their relationship to study intervention assessed by the clinician who examines and evaluates the participant based on temporal relationship and his/her clinical judgment. The degree of certainty about causality will be graded using the categories below. In a clinical trial, the study product must always be suspect.

- **Definitely Related** – There is clear evidence to suggest a causal relationship, and other possible contributing factors can be ruled out. The clinical event, including an abnormal laboratory test result, occurs in a plausible time relationship to study intervention administration and cannot be explained by concurrent disease or other drugs or chemicals. The response to withdrawal of the study intervention (dechallenge) should be clinically plausible. The event must be pharmacologically or phenomenologically definitive, with use of a satisfactory rechallenge procedure if necessary.
- **Probably Related** – There is evidence to suggest a causal relationship, and the influence of other factors is unlikely. The clinical event, including an abnormal laboratory test result, occurs within a reasonable time after administration of the study intervention, is unlikely to be attributed to concurrent disease or other drugs or chemicals, and follows a clinically reasonable response on withdrawal (dechallenge). Rechallenge information is not required to fulfill this definition.
- **Potentially Related** – There is some evidence to suggest a causal relationship (e.g., the event occurred within a reasonable time after administration of the trial medication). However, other factors may have contributed to the event (e.g., the participant’s clinical condition, other concomitant events). Although an AE may rate only as “possibly related” soon after discovery, it

© WEIRC 2018 – All rights reserved

can be flagged as requiring more information and later be upgraded to "probably related" or "definitely related", as appropriate.

- **Unlikely to be related** – A clinical event, including an abnormal laboratory test result, whose temporal relationship to study intervention administration makes a causal relationship improbable (e.g., the event did not occur within a reasonable time after administration of the study intervention) and in which other drugs or chemicals or underlying disease provides plausible explanations (e.g., the participant's clinical condition, other concomitant treatments).
- **Not Related** – The AE is completely independent of study intervention administration, and/or evidence exists that the event is definitely related to another etiology. There must be an alternative, definitive etiology documented by the clinician.

8.3.3.3 EXPECTEDNESS

Principal investigator will be responsible for determining whether an adverse event (AE) is expected or unexpected. An AE will be considered unexpected if the nature, severity, or frequency of the event is not consistent with the risk information previously described for the study intervention.

8.3.4 TIME PERIOD AND FREQUENCY FOR EVENT ASSESSMENT AND FOLLOW-UP

The occurrence of an adverse event (AE) or serious adverse event (SAE) may come to the attention of study personnel during study visits and interviews of a study participant presenting for medical care, or upon review by a study monitor.

All AEs including local and systemic reactions not meeting the criteria for SAEs will be captured on the appropriate case report form (CRF). Information to be collected includes event description, time of onset, clinician's assessment of severity, relationship to study product (assessed only by those with the training and authority to make a diagnosis), and time of resolution/stabilization of the event. All AEs occurring while on study must be documented appropriately regardless of relationship. All AEs will be followed to adequate resolution.

Any medical condition that is present at the time that the participant is screened will be considered as baseline and not reported as an AE. However, if the study participant's condition deteriorates at any time during the study, it will be recorded as an AE.

Changes in the severity of an AE will be documented to allow an assessment of the duration of the event at each level of severity to be performed. AEs characterized as intermittent require documentation of onset and duration of each episode.

Principal investigator will record all reportable events with start dates occurring any time after informed consent is obtained until 7 (for non-serious AEs) or 30 days (for SAEs) after the last day of study participation. At each study visit, the investigator will inquire about the occurrence of AE/SAEs since the last visit. Events will be followed for outcome information until resolution or stabilization.

8.3.5 ADVERSE EVENT REPORTING

© WEIRC 2018 – All rights reserved

Any Adverse or intercurrent Event occurring during the study period, spontaneously reported by the subject or observed by others, will be recorded in the Adverse Event Form in the Case Report Form. The records will describe:

- Date of onset
- Equipment worn when the adverse event appeared
- Detailed description of the adverse event (nature of event, signs and symptoms)
- Intensity
- Adverse Event or Serious Adverse Event (reason if Serious Adverse Event)
- Plausible relationship to study equipment (according to Investigator's opinion)
- Progress
- Date of end

Any Adverse Event or Serious Adverse Event will be forwarded to the Study Manager (ESSILOR R&D) as soon as the Investigator will become aware.

8.3.6 SERIOUS ADVERSE EVENT REPORTING

The study investigator shall complete an Unanticipated Adverse Device Effect Form and submit to the study sponsor and to the reviewing Institutional Review Board (IRB) as soon as possible, but in no event later than 10 working days after the investigator first learns of the effect. The study sponsor is responsible for conducting an evaluation of an unanticipated adverse device effect and shall report the results of such evaluation to the Food and Drug Administration (FDA) and to all reviewing IRBs and participating investigators within 10 working days after the sponsor first receives notice of the effect. Thereafter, the sponsor shall submit such additional reports concerning the effect as FDA requests.

8.3.7 REPORTING EVENTS TO PARTICIPANTS

AEs and SAEs related to study intervention will be reported to participants through a formal letter sent either physically or using an electronic platform.

8.4 UNANTICIPATED PROBLEMS

8.4.3 DEFINITION OF UNANTICIPATED PROBLEMS (UP)

Unanticipated problems involving risks to participants or others to include, in general, any incident, experience, or outcome that meets **all** of the following criteria:

- Unexpected in terms of nature, severity, or frequency given (a) the research procedures that are described in the protocol-related documents, such as the Institutional Review Board (IRB)-approved research protocol and informed consent document; and (b) the characteristics of the participant population being studied;
- Related or possibly related to participation in the research ("possibly related" means there is a reasonable possibility that the incident, experience, or outcome may have been caused by the procedures involved in the research); and
- Suggests that the research places participants or others at a greater risk of harm (including physical, psychological, economic, or social harm) than was previously known or recognized.

© WEIRC 2018 – All rights reserved

8.4.4 UNANTICIPATED PROBLEM REPORTING

The investigator will report unanticipated problems (UPs) to the reviewing Institutional Review Board (IRB) and to the lead principal investigator (PI). The UP report will include the following information:

- Protocol identifying information: protocol title and number, PI's name, and the IRB project number;
- A detailed description of the event, incident, experience, or outcome;
- An explanation of the basis for determining that the event, incident, experience, or outcome represents an UP;
- A description of any changes to the protocol or other corrective actions that have been taken or are proposed in response to the UP.

To satisfy the requirement for prompt reporting, UPs will be reported using the following timeline:

- UPs that are serious adverse events (SAEs) will be reported to the IRB and to the study sponsor within 10 days of the investigator becoming aware of the event.
- Any other UP will be reported to the IRB and to the study sponsor within 30 days of the investigator becoming aware of the problem.
- All UPs should be reported to appropriate institutional officials (as required by an institution's written reporting procedures), the supporting agency head (or designee), and the Office for Human Research Protections (OHRP) within 30 days of the IRB's receipt of the report of the problem from the investigator.

9 STATISTICAL CONSIDERATIONS

9.3 STATISTICAL HYPOTHESES

The aim is to conduct an RCT that has clinically significant impact of 30% improvement in spherical equivalent of cycloplegic autorefraction in at least one eye after 2 years compared to control arm, AND at least 0.50D difference in myopia progression in at least one eye after 2 years compared to control arm using spherical equivalent of cycloplegic autorefraction, AND change in axial length of 0.3mm using conversion of 0.10mm = 0.28D in at least one eye in one test arm compared to control arm.

- Primary Efficacy Endpoint(s): HAL or SAL more superior than SVL
    - 33% improvement in spherical equivalent of cycloplegic autorefraction in at least one eye after 2 years compared to control arm, AND 0.50D difference in myopia progression in at least one eye after 2 years compared to control arm using spherical equivalent of cycloplegic autorefraction, AND change in axial length of 0.3mm using conversion of 0.10mm = 0.28D in at least one eye in one test arm compared to control arm.
- Secondary Efficacy Endpoint(s): HAL or SAL equivalence to SVL

© WEIRC 2018 – All rights reserved

    - 80% or equivalent compliance and adaptation in test arm compared to control arm.

- Exploratory Endpoint(s): HAL or SAL is equivalent to SVL
    - No difference in peripheral cycloplegic autorefraction
    - No difference in peripheral axial length
    - No difference in choroidal thickness

9.4 SAMPLE SIZE DETERMINATION

With an expected 33% reduction in mean myopia progression in FIN lenses compared with SVLs, together with a study by Yang et al. that found a mean myopia progression of 1.50 D over 2 years in Chinese children wearing SVLs. A 33% treatment effect over 2 years is therefore about 0.50D.

An overall standard deviation (s.d.) of 0.6D for the magnitude of myopia progression in two years was derived from the study in myopia progression of PALs in Chinese children by Yang et al [32]. We estimated a higher error in our study and increased the standard deviation to 0.75 with the following determinant.

1. At least a 90% statistical power.
2. Type 1 error probability (α) of 0.05, based on a two-tailed t-test.
3. 1:1:1 sample ratio.
4. Adjustment for multiplicity of 2 endpoints.
5. Allowing a maximum drop-out rate of 10%

k = n_2/n_1 = 1

n_1 = (sigma_1^2 + sigma_2^2/K) *(z_1-alpha/2 + z_1-beta) ^2/Delta^2

n_1 = (0.75^2 + 0.75^2/1) *(1.96 + 1.28) ^2/0.5^2

n_1 = 47

n_2 = K*n_1 = 47

n_1 = 47*10% ~ 50

3 treatment groups = 50 * 3 = 150

9.5 POPULATIONS FOR ANALYSES

Intention-to-Treat (ITT) Analysis Dataset (i.e., all randomized participants) will be used for primary and secondary endpoints analysis and a subset of the participants based on Per-Protocol Analysis will be used as adjusted endpoints.

© WEIRC 2018 – All rights reserved

- Per-Protocol Analysis Dataset: defines a subset of the participants in the full analysis (ITT) set who complied with the protocol sufficiently to ensure that these data would be likely to represent the effects of study intervention according to the underlying scientific model (e.g., participants who took at least 80% of study intervention for 80% of the days within the maintenance period)

9.6 STATISTICAL ANALYSES

9.6.3 GENERAL APPROACH

All efficacy variables will be listed by subject. Data will be summarized by treatment group. N, Mean, Standard Deviation, Minimum and Maximum will summarize continuous efficacy variables, whereas number and percent will summarize categorical efficacy variables.

All analyses of the continuous efficacy variables (e.g., cycloplegic autorefraction and axial length) will be performed as analysis of variance with treatment group. Treatment groups will be tested at the 2-sided 5% significance level.

All assumptions for regression models will be assessed by viewing plots of the residual values

All analyses of categorical efficacy measures will be performed using logistic regression with treatment group and adjustments for variables that were statistically different in each group.

9.6.4 ANALYSIS OF THE PRIMARY EFFICACY ENDPOINT(S)

Repeated measure ANOVA will be used to compare cycloplegic autorefraction and axial length with control group. Changes in both variables will compared to control group will be analyses using ANOVA.

9.6.5 ANALYSIS OF THE SECONDARY ENDPOINT(S)

Proportion of population without myopia progression will be compared with control group. Proportion with 80% compliance with device will also be compared with control group using Fisher exact test based on outcome of questionnaires.

9.6.6 SAFETY ANALYSES

Will be based on adverse events and serious adverse events reporting.

9.6.7 BASELINE DESCRIPTIVE STATISTICS

© WEIRC 2018 – All rights reserved

All continuous variables will be summarized using the following descriptive statistics: n (non-missing sample size), mean, standard deviation, maximum and minimum. The frequency and percentages (based on the non-missing sample size) of observed levels will be reported for all categorical measures. In general, all data will be listed, sorted by treatment and subject, and when appropriate by visit number within subject. All summary tables will be structured with a column for each treatment in the order (Control, Treatment) and will be annotated with the total population size relevant to that table/treatment, including any missing observations.

9.6.8 PLANNED INTERIM ANALYSIS

Interim analysis will be done after 1-year follow up. Blind analysis will be carried out by a third party statistician that is independent with no interest and involvement in the clinical trial. Results from the blind analysis will be shared with the Data and Safety Monitoring Committee (DSMC). DSMC will advise if the clinical trial is safe to be continued and the sponsor can decide for early disclosure.

Blind analysis is done by removing all identifiable variables like subject identification and birth dates after including the masked concepts variable by the one personnel with the masked information. The data will be scrambled before it is passed to the third party statistician for analysis.

9.6.9 SUB-GROUP ANALYSES

Subgroup analysis will only be done for variables that were not randomized equally. The main variables for randomization were cycloplegic autorefraction, age and gender which are important variables for primary endpoint. The other variable that will be included in subgroup analysis will be axial length should there be any between group difference during baseline.

Other demographic variables like age of myopia onset, parental myopia, height and weight will be analyzed for their effect on myopia progression.

9.6.10 TABULATION OF INDIVIDUAL PARTICIPANT DATA

The following diagram will be filled up at the end of the study.

© WEIRC 2018 – All rights reserved

Subject Disposition Flow Diagram

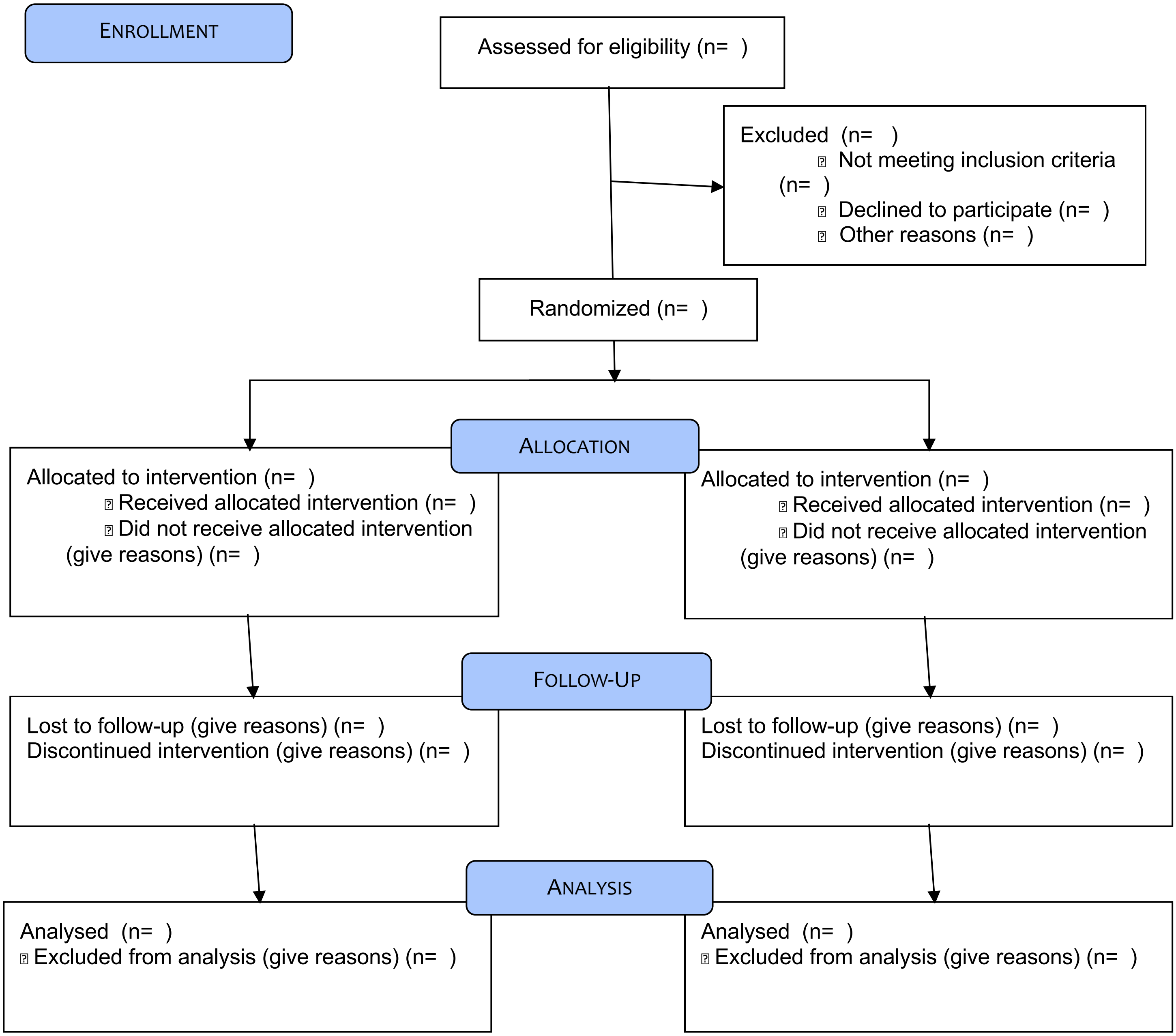

9.6.11 EXPLORATORY ANALYSES

Exploratory outcomes like peripheral autorefraction and axial length, and choroidal thickness will be compared with the control group using repeated measure ANOVA.

© WEIRC 2018 – All rights reserved

10 SUPPORTING DOCUMENTATION AND OPERATIONAL CONSIDERATIONS

10.3 REGULATORY, ETHICAL, AND STUDY OVERSIGHT CONSIDERATIONS

No text is to be entered in this section; rather it should be included under the relevant subheadings below.

The following subsections should include a description of the regulatory and ethical considerations, and context for the conduct of the trial. Of note, the guiding ethical principles being followed by this study are included in the ***Statement of Compliance*** *at the beginning of this protocol. For NIH Intramural Research Program studies only: A statement referencing compliance with NIH Human Research Protections Program policies and procedures is adequate for* ***Subsection 10.1.1, Informed Consent Process.***

10.3.3 INFORMED CONSENT PROCESS

Before starting this study, the protocol will be submitted to the Ethics Committee of the study center for evaluation. The study will not start before the Ethics Committee gives written approval as required.

Conduct of the study will strictly follow the approved protocol and protocol amendments.

This study adhered to the tenets of the Declaration of Helsinki.

This study will be conducted in full accordance with the current version of: study protocol, protocol amendments, ESSILOR R&D Standard Operating Procedures, and any applicable national and local laws and regulations. Information regarding any study centers participating in this study that cannot comply with these standards will be documented.

Agreement of the WEIRC to conduct and administer this study in accordance with the protocol will be documented in separate study agreements in the Collaborative Research Project with Essilor International (Wenzhou Medical University grant numbers 95013006, 95016010 and 95020005).

The Principal Investigator is responsible for giving information about the study to all staff members involved in the study or in any element of subject management, both before starting the practical performance of the study and during the course of the study (e.g. when new staff become involved).

The Principal Investigator, Study Examiner, Study Coordinator and Study Manager (WEIRC) are responsible for ensuring the privacy of the subjects during the study, and must ensure that a fully functional examination equipment and a personnel trained in its proper use are immediately available. The Principal Investigator, Study Examiner, Study Coordinator and Study Manager (WEIRC) must be familiar with the background and requirements of the study and with the properties of the lenses used.

The Principal Investigator has the overall responsibility for the conduct and administration of the study at WEIRC and for contacts with the study center management and the Ethics Committee.

© WEIRC 2018 – All rights reserved

10.3.3.1 CONSENT/ASSENT AND OTHER INFORMATIONAL DOCUMENTS PROVIDED TO PARTICIPANTS

Any information about the study must be given to the subjects before their decision to participate or abstain from participation.

The Informed Consent Form used by the Study Examiner for obtaining the subject's Informed Consent must be reviewed and approved by Study Managers and PI prior to submission to the appropriate Ethics Committee for approval / favorable opinion.

The Study Examiner (WEIRC) should fully inform all subjects of all pertinent aspects of the study including the written and oral information about the study in a language understandable by the subject.

Written Informed Consent will be obtained from each subject before any procedures or assessments are done and after the aims, methods, anticipated benefits, potential hazards, and insurance arrangements in force are explained. It will also be explained to the subjects that they are free to refuse entry into the study and to withdraw from the study at any time without prejudice.

The subject’s willingness to participate in the study will be documented in writing in a consent form, which will be signed and personally dated by the subject and the person who conducted the informed consent discussion. The Informed Consent Form will be made in two originals copies: one copy will be kept by the Investigator, the other copy will be given to the subject.
1.

10.3.4 SUBJECT CONFIDENTIALITY

All information from this study will be entered into a Database at the WEIRC .

10.3.4.1 CONSENT PROCEDURES AND DOCUMENTATION

Informed consent is a process that is initiated prior to the individual’s agreeing to participate in the study and continues throughout the individual’s study participation. Consent forms will be Institutional Review Board (IRB)-approved and the participant will be asked to read and review the document. The investigator will explain the research study to the participant and answer any questions that may arise. A verbal explanation will be provided in terms suited to the participant’s comprehension of the purposes, procedures, and potential risks of the study and of their rights as research participants. Participants will have the opportunity to carefully review the written consent form and ask questions prior to signing. The participants should have the opportunity to discuss the study with their family or surrogates or think about it prior to agreeing to participate. The participant will sign the informed consent document prior to any procedures being done specifically for the study. Participants must be informed that participation is voluntary and that they may withdraw from the study at any time, without prejudice. A copy of the informed consent document will be given to the participants for their records. The informed consent process will be conducted and documented in the source document (including the date), and the form signed, before the participant undergoes any study-specific procedures. The rights and welfare of the participants will be protected by emphasizing to them that the quality of their medical care will not be adversely affected if they decline to participate in this study.

© WEIRC 2018 – All rights reserved

10.3.5 STUDY DISCONTINUATION AND CLOSURE

This study may be temporarily suspended or prematurely terminated if there is sufficient reasonable cause. Written notification, documenting the reason for study suspension or termination, will be provided by the suspending or terminating party to study participants and investigator. If the study is prematurely terminated or suspended, the Principal Investigator (PI) will promptly inform study participants, the Institutional Review Board (IRB), and sponsor and will provide the reason(s) for the termination or suspension. Study participants will be contacted, as applicable, and be informed of changes to study visit schedule.

Circumstances that may warrant termination or suspension include, but are not limited to:

- Determination of unexpected, significant, or unacceptable risk to participants
- Demonstration of efficacy that would warrant stopping
- Insufficient compliance to protocol requirements
- Data that are not sufficiently complete and/or evaluable
- Determination that the primary endpoint has been met
- Determination of futility

Study may resume once concerns about safety, protocol compliance, and data quality are addressed, and satisfy the WEIRC and IRB.

10.3.6 CONFIDENTIALITY AND PRIVACY

Participant confidentiality and privacy is strictly held in trust by the participating investigators, their staff, and the sponsor(s) and their interventions. This confidentiality is extended to cover testing of biological samples and genetic tests in addition to the clinical information relating to participants. Therefore, the study protocol, documentation, data, and all other information generated will be held in strict confidence. No information concerning the study or the data will be released to any unauthorized third party without prior written approval of the sponsor.

All research activities will be conducted in as private a setting as possible.

The study monitor, other authorized representatives of the sponsor, representatives of the Institutional Review Board (IRB), regulatory agencies or pharmaceutical company supplying study product may inspect all documents and records required to be maintained by the investigator, including but not limited to, medical records (office, clinic, or hospital) and pharmacy records for the participants in this study. The clinical study site will permit access to such records.

The study participant’s contact information will be securely stored at each clinical site for internal use during the study. At the end of the study, all records will continue to be kept in a secure location for as long a period as dictated by the reviewing IRB, Institutional policies, or sponsor requirements.

Study participant research data, which is for purposes of statistical analysis and scientific reporting, will be transmitted to and stored at the WEIRC. This will not include the participant’s contact or identifying information. Rather, individual participants and their research data will be identified by a unique study identification number. The study data entry and study management systems used by clinical sites and by

© WEIRC 2018 – All rights reserved

WEIRC research staff will be secured and password protected. At the end of the study, all study databases will be de-identified and archived at the WEIRC.

All materials, information (oral or written) and unpublished documentation provided to WEIRC, including, without limitation, this protocol and protocol amendments, the subject Case Report Forms and ESSILOR R&D equipment, related documentation, may not be given or disclosed, either in part or in whole, by WEIRC or by any person under his/her authority to any third party without the prior written consent of WEIRC Scientific committee.

However, the submission of this protocol and other necessary documentation to the Ethics Committee is expressly permitted, their members having the same obligation of confidentiality.

WEIRC will consider all information, results, discoveries, records accumulated, acquired, or deduced in the course of the study, other than information to be disclosed by law, as confidential and shall not disclose any such results, discoveries, records to any third party without WEIRC Scientific committee prior written consent.

10.3.7 KEY ROLES AND STUDY GOVERNANCE

- **STUDY CENTER**

Eye Hospital, Wenzhou Medical University
WEIRC
270 Xueyuan West Road,
Wenzhou, Zhejiang.
China 325027.

<u>The Principal Investigator is:</u>

BAO Jinhua
Eye Hospital, Wenzhou Medical University
WEIRC
270 Xueyuan West Road,
Wenzhou, Zhejiang.
China 325027.
Phone: +86 577 88068166
E-mail: baojessie@163.com

If a study is conducted by a team of individuals at a study center, the Principal Investigator is the responsible leader of the team.

The Principal Investigator may delegate some of his/her duties to Study Manager and other study center staff members like the study coordinator by notifying and signing a delegation of duties form.
When tasks are delegated by the Principal Investigator, he/she is responsible for providing adequate training and supervision to Study Manager and other study centre staff members.

© WEIRC 2018 – All rights reserved

Funding
International S&T Cooperation Program of China (2014DFA30940)
Collaborative Research Project with Essilor International (Wenzhou Medical University grant numbers 95013006, 95016010 and 95020005).

Study Device Sponsor
ESSILOR INTERNATIONAL
147, rue de Paris
94220 Charenton-le-Pont – France

The Vision Scientist from Essilor is:
Adeline YANG
Centre for Innovation & Technologies AMERA
Essilor R&D Centre Singapore
215 Kallang Bahru, #02-00
Essilor Building
Singapore 339346
Phone: +65 67134607 Fax: +65 63968759
E-mail: adeline.yang@essilor.com.sg

The Study Manager is:
JIN Wanqing
Eye Hospital, Wenzhou Medical University
270 Xueyuan Road
Wenzhou, China
Post code: 325027
Phone: +86 57788068166
Email: *wcyjqw@163.com*

LIM Ee Woon
Centre for Innovation & Technologies AMERA
Essilor R&D Centre Singapore
215 Kallang Bahru, #02-00
Essilor Building
Singapore 339346
Phone: +65 67134632 Fax: +65 63968759
E-mail: limew@essilor.com.sg

The Study Coordinator is:
HUANG Yingying
Eye Hospital, Wenzhou Medical University
WEIRC
270 Xueyuan Road,
Wenzhou, Zhejiang.
China 325027.

© WEIRC 2018 – All rights reserved

Phone: +86 57788067965
E-mail: 1097983413@qq.com

The Vision Scientist from ESSILOR R&D Centre is the Sponsor representative and works for ESSILOR Research & Development (R&D). The Study Manager from ESSILOR R&D will be responsible for the randomisation and logistical flow of lens ordering, edging and verification for each order. The Study Manager from the Eye Hospital of Wenzhou Medical University will be responsible for recruitment and the issue of study subject identification code to all registered participants. The Study Coordinator from the Eye Hospital of Wenzhou Medical University will be in charge of the eye examination and data management to ensure the quality of data. The Principal Investigator is responsible for making sure that the whole study runs in conformance with the study protocol.

10.3.8 SAFETY OVERSIGHT

Safety oversight will be under the direction of a Data and Safety Monitoring Board (DSMB) composed of individuals with the appropriate expertise, including 1 ophthalmologist, Chen Yun Yun and 2 biostatisticians, Zheng Jing Wei and Zhuo Wei He. Members of the DSMB should be independent from the study conduct and free of conflict of interest, or measures should be in place to minimize perceived conflict of interest. The DSMB will meet at least semiannually to assess safety and efficacy data on each arm of the study. The DMSB will operate under the rules of an approved charter that will be written and reviewed at the organizational meeting of the DSMB. At this time, each data element that the DSMB needs to assess will be clearly defined. The DSMB will provide its report to WEIRC.

<u>Data & Safety Monitoring Board:</u>
CHEN Yunyun
ZHENG Jingwei
ZHOU Weihe

10.3.9 CLINICAL MONITORING

Clinical site monitoring is conducted to ensure that the rights and well-being of trial participants are protected, that the reported trial data are accurate, complete, and verifiable, and that the conduct of the trial is in compliance with the currently approved protocol/amendment(s), with International Conference on Harmonisation Good Clinical Practice (ICH GCP), and with applicable regulatory requirement(s).

- Monitoring for this study will be performed by DSMC and PI.
- On-site, centralized, at least bi-annual random review of CRF and observation of the conduct of study will be done. 100% of data monitoring will be done by PI to prevent missing data and ensure safety of trial.
- WEIRC Scientific Committee will be provided copies of monitoring reports within 14 days of visit.
- Details of clinical site monitoring are documented in a Clinical Monitoring Plan (CMP). The CMP describes in detail who will conduct the monitoring, at what frequency monitoring will be done, at what level of detail monitoring will be performed, and the distribution of monitoring reports.

© WEIRC 2018 – All rights reserved

- Independent audits will be conducted by the ethics committee to ensure monitoring practices are performed consistently across all participating sites and that monitors are following the CMP.

10.3.10 GENERAL MONITORING

This study will be managed and monitored by the Principal Investigator. All study data will be transferred and stored in a database at WEIRC.

It is the responsibility of the Study Examiner, Study coordinator and Study Manager (WEIRC) collecting data from participants to ensure that the study is fully compliant with the protocol, protocol amendments, WEIRC Standard Operating Procedures and that valid data are entered into the Case Report Forms. The Study Examiner (WEIRC) will be required to sign and date the Case Report Forms after each visit.

In case of poor compliance with the protocol, protocol amendments and WEIRC Standard Operating Procedures, the reason for the discrepancy will have to be documented.

To achieve this objective, the Study Manager's duties are to aid the Study Examiner, Study coordinator and Study Manager in the maintenance of complete, legible, well-organized and easily retrievable data. The Vision Scientist will review the protocol with the Study Examiner, Study Coordinator and Study Manager. In addition, the Principal Investigator will explain to the Study Examiner, Study Coordinator and Study Manager reporting responsibilities and all applicable regulations concerning the evaluation of the study lenses.

10.3.11 ONSITE MONITORING

At the beginning of the study, an initiation visit will be performed to train the staff of the study center to the protocol.

Throughout the course of the study, the Study Manager will make frequent contacts with the Study Examiner, Study Coordinator and Study Manager; these will include phone calls and on-site monitoring visits. Monitoring visits will be performed by the Study Manager and/or Principal Investigator to ensure that the study center is adhering to the protocol at every point of the study (at least one monitoring visit will be performed). The Monitoring part will be done in accordance with the Monitoring Standard Operating Procedures described below.

As part of the monitoring visit, source documents will be made available for review by the Principal Investigator. The Vision Scientist and Study Manager will also request a review of the Study Examiner, Study coordinator and Study Manager's study files to ensure completeness of documentation (Case Report Form, Informed Consent Forms etc…) in all respects of study conduct. The Study Manager (ESSILOR R&D) will also perform lens accountability checks, and will remain masked regarding study lenses identification.

During the monitoring visit, the PI will perform the following priority checks:

© WEIRC 2018 – All rights reserved

- All the Informed Consent Forms
- All the Adverse Events and Serious Adverse Events
- All the eligibility criteria
- Consistency of available data regarding the protocol (visit schedule, Case Report Forms)
- All the available equipment

Moreover, the Vision Scientist and Study Manager (ESSILOR R&D) could observe screening visits and/or Visits 2, Visits 3 etc… of study participants.

On completion of the study, the Principal Investigator will arrange for a final review of the study documents, after which the files should be secured for the appropriate time period.

If all subjects have not completed the study at the moment of the last monitoring visit, remaining verifications will be done by the Principal Investigator.

During these on-site monitoring visits, the Vision Scientist will cooperate in providing the documents for control and responses to inquiries.

10.3.12 QUALITY ASSURANCE AND QUALITY CONTROL

Quality control (QC) procedures will be implemented beginning with the data entry system and data QC checks that will be run on the database will be generated. Any missing data or data anomalies will be communicated to the site(s) for clarification/resolution.

Following written Standard Operating Procedures (SOPs), the monitors will verify that the clinical trial is conducted and data are generated and biological specimens are collected, documented (recorded), and reported in compliance with the protocol, International Conference on Harmonisation Good Clinical Practice (ICH GCP), and applicable regulatory requirements (e.g., Good Laboratory Practices (GLP), Good Manufacturing Practices (GMP)).

The investigational site will provide direct access to all trial related sites, source data/documents, and reports for the purpose of monitoring and auditing by the sponsor, and inspection by local and regulatory authorities.

For the purpose of ensuring compliance with the protocol and applicable regulatory requirements, the Investigator should permit auditing by ESSILOR R&D. The Investigator agrees to allow the auditors to have direct access to his/her study records for review, being understood that the auditors are bound by professional secrecy, and as such will not disclose any personal identity or any confidential information.

10.3.13 DATA HANDLING AND RECORD KEEPING

10.3.13.1 DATA COLLECTION AND MANAGEMENT RESPONSIBILITIES

© WEIRC 2018 – All rights reserved

Data collection is the responsibility of the clinical trial staff at the site under the supervision of the site investigator. The investigator is responsible for ensuring the accuracy, completeness, legibility, and timeliness of the data reported.

All source documents should be completed in a neat, legible manner to ensure accurate interpretation of data.

Hardcopies of the study visit worksheets will be provided for use as source document worksheets for recording data for each participant enrolled in the study. Data recorded in the electronic case report form (eCRF) derived from source documents should be consistent with the data recorded on the source documents.

Clinical data (including adverse events (AEs), concomitant medications, and expected adverse reactions data) and clinical laboratory data will be entered into a database. The data system includes password protection and internal quality checks, such as automatic range checks, to identify data that appear inconsistent, incomplete, or inaccurate. Clinical data will be entered directly from the source documents.

Data management process will be conducted and performed by WEIRC.

All Case Report Forms will be tracked and reviewed by WEIRC before being entered in the database. The Source data and raw data will be checked by ESSILOR R&D for consistency and plausibility. Data clarification will be generated (if needed) for resolution by the Investigator.

After database lock, the data will be transferred for statistical analysis (with anonymous subject identification codes).

10.3.13.2 STUDY RECORDS RETENTION

The Investigator:

- Keeps all study-related documents in appropriate file folders. Records of subjects, original Informed Consent Forms, Source documents, Case Report Forms, Materials Inventory, Ethic Committee approval and Sponsor correspondence pertaining to the study must be kept on file. This concerns all subjects included in the study or not.

- Retains a list of the subjects names, addresses (and/or number of subject file), subject identification codes, dates of entry into and completion of the trial period, to allow checking of data reported on Case Report Forms with those from source documents.

The study related documents:

- Must be retained as strictly confidential at the study center during all the course of the study

© WEIRC 2018 – All rights reserved

- Must be kept in a secure area all along the study
- Must be returned to WEIRC after completion or discontinuation of the study.
- A copy of Case Report Forms completed must be kept for a duration defined according to the type of the study in a way that allows an accurate reporting, interpretation and verification.

10.3.14 PROTOCOL DEVIATIONS

A protocol deviation is any noncompliance with the clinical trial protocol, International Conference on Harmonisation Good Clinical Practice (ICH GCP). The noncompliance may be either on the part of the participant, the investigator, or the study site staff. As a result of deviations, corrective actions are to be developed by the site and implemented promptly.

These practices are consistent with ICH GCP:

- 4.5 Compliance with Protocol, sections 4.5.1, 4.5.2, and 4.5.3
- 5.1 Quality Assurance and Quality Control, section 5.1.1
- 5.20 Noncompliance, sections 5.20.1, and 5.20.2.

It is the responsibility of the site investigator to use continuous vigilance to identify and report deviations within 7 working days of identification of the protocol deviation, or within 7 working days of the scheduled protocol-required activity. All deviations must be addressed in study source documents, reported to principal investigator. Protocol deviations must be sent to the reviewing Institutional Review Board (IRB) per their policies. The site investigator is responsible for knowing and adhering to the reviewing IRB requirements.

10.3.15 PUBLICATION AND DATA SHARING POLICY

This study will be conducted in accordance with the following publication and data sharing policies and regulations:

Eye Hospital ensures that the public has access to the published results of research. It requires scientists to submit final peer-reviewed journal manuscripts upon acceptance for publication.

This trial will be registered at chiClinicalTrials.gov, and results information from this trial will be submitted to chiClinicalTrials.gov. In addition, every attempt will be made to publish results in peer-reviewed journals. Data from this study may be requested from other researchers 2 years after the completion of the primary endpoint by contacting CHEN Hao from the Eye Hospital of Wenzhou Medical University .

10.3.16 CONFLICT OF INTEREST POLICY

The independence of this study from any actual or perceived influence, such as by the pharmaceutical industry, is critical. Therefore, any actual conflict of interest of persons who have a role in the design, conduct, analysis, publication, or any aspect of this trial will be disclosed and managed. Furthermore,

© WEIRC 2018 – All rights reserved

persons who have a perceived conflict of interest will be required to have such conflicts managed in a way that is appropriate to their participation in the design and conduct of this trial. The study leadership in conjunction with WEIRC has established policies and procedures for all study group members to disclose all conflicts of interest and will establish a mechanism for the management of all reported dualities of interest.

10.4 APPENDICES

Appendix : Clinical Trial Standard Operating Procedure (SOP)

FIN Clincal Trial SOP Jun14.docx

© WEIRC 2018 – All rights reserved

10.5 ABBREVIATIONS

The list below includes abbreviations utilized in this template. However, this list should be customized for each protocol (i.e., abbreviations not used should be removed and new abbreviations used should be added to this list).

AE	Adverse Event
ANCOVA	Analysis of Covariance
CFR	Code of Federal Regulations
CLIA	Clinical Laboratory Improvement Amendments
CMP	Clinical Monitoring Plan
COC	Certificate of Confidentiality
CONSORT	Consolidated Standards of Reporting Trials
CRF	Case Report Form
DCC	Data Coordinating Center
DHHS	Department of Health and Human Services
DSMB	Data Safety Monitoring Board
DRE	Disease-Related Event
EC	Ethics Committee
eCRF	Electronic Case Report Forms
FDA	Food and Drug Administration
FDAAA	Food and Drug Administration Amendments Act of 2007
FFR	Federal Financial Report
GCP	Good Clinical Practice
GLP	Good Laboratory Practices
GMP	Good Manufacturing Practices
GWAS	Genome-Wide Association Studies
HIPAA	Health Insurance Portability and Accountability Act
IB	Investigator’s Brochure
ICH	International Conference on Harmonisation
ICMJE	International Committee of Medical Journal Editors
IDE	Investigational Device Exemption
IND	Investigational New Drug Application
IRB	Institutional Review Board
ISM	Independent Safety Monitor
ISO	International Organization for Standardization
ITT	Intention-To-Treat
LSMEANS	Least-squares Means

© WEIRC 2018 – All rights reserved

MedDRA	Medical Dictionary for Regulatory Activities
MOP	Manual of Procedures
MSDS	Material Safety Data Sheet
NCT	National Clinical Trial
NIH	National Institutes of Health
NIH IC	NIH Institute or Center
OHRP	Office for Human Research Protections
PI	Principal Investigator
QA	Quality Assurance
QC	Quality Control
SAE	Serious Adverse Event
SAP	Statistical Analysis Plan
SMC	Safety Monitoring Committee
SOA	Schedule of Activities
SOC	System Organ Class
SOP	Standard Operating Procedure
UP	Unanticipated Problem
US	United States

Arm: Group according to the lens worn (Reference lens or Test lens) where the subject will be assigned

Case Report Form (CRF): A printed, optical, or electronic document designed to record all of the protocol - required information to be reported to the sponsor on each subject

Compliance: Adherence to all the study related requirements and the applicable regulatory requirements.

Documentation: All records, in any form (including but not limited to, written, electronic, magnetic, and optical records) that describe or record the methods, conduct, and/or results of a study, the factors affecting a study, and the actions taken.

Essential documents: Documents that individually and collectively permit evaluation of the conduct of the study and the quality of the data produced.

Ethics Committee / Independent Ethics Committee: An independent body from Wenzhou Medical University (a review board or a committee, institutional, regional, national or supranational), constituted of medical/scientific professionals and nonmedical/nonscientific members, whose responsibility is to ensure the protection of the rights, safety, and well-being of human subjects involved in a study and to provide public assurance of that protection, by, among other things reviewing and approving/providing favorable opinion on the trial protocol the suitability of the investigator(s), facilities, and the methods and material to be used in obtaining and documenting informed consent of the study subjects.

Principal investigator: Person overseeing the scientific and technical aspects of the study, and has responsibility for the management of the research and investigator staff in an investigation site.

Study Manager (ESSILOR R&D): Sponsor representative. Person responsible for randomization and lens logistics.

© WEIRC 2018 – All rights reserved

Study Manager (WEIRC): A person responsible for the conduct of the study at a study center. If a study is conducted by a team of individuals at a study center, the Study Manager is the responsible leader of the team. The study manager is supervised by the Principal Investigator.

Study Coordinator: Any individual member designated and supervised by the Principal Investigator to perform critical study related procedures and/or to make important study related decisions.

Informed consent: A process by which a subject voluntarily confirms his or her willingness to participate in a particular study, after having been informed of all aspects of the study that are relevant to the subject's decision to participate. Informed consent is documented by means of written, signed and dated informed consent form

Monitoring: The act of overseeing the progress of a study, and of ensuring that it is conducted, recorded, and reported in accordance with the protocol, standard operating procedures (SOPs), and the applicable regulatory requirements.

Randomization: The process of assigning subject to an arm using an element of chance to determine the assignments in order to reduce bias.

Ratio: Indicator of subject distribution in arm or Reference/Test-groups.

Sponsor: An individual company, institution, or organization that takes responsibility for the initiation, management, and/or financing of a study

Study Master File: File which contains all the essential documents related to a study, before the study commences, during study conduct and after the completion of study.

© WEIRC 2018 – All rights reserved

REFERENCES

1. Abelson, MB., Rosner, SA., MacDonald, N. (2003). How to Be Comfortable With Painkillers. *Review of Ophthalmology.*
2. Adler, D. and Millodot, M. (2006). The possible effect of undercorrection on myopic progression in children. *Clinical and Experimental Optometry*, 89(5), pp.315-321.
3. Anstice, N. and Phillips, J. (2011). Effect of Dual-Focus Soft Contact Lens Wear on Axial Myopia Progression in Children. *Ophthalmology*, 118(6), pp.1152-1161.
4. Berntsen, D., Barr, C., Mutti, D. and Zadnik, K. (2013). Peripheral Defocus and Myopia Progression in Myopic Children Randomly Assigned to Wear Single Vision and Progressive Addition Lenses. *Investigative Opthalmology & Visual Science*, 54(8), p.5761.
5. Berntsen, D., Mutti, D. and Zadnik, K. (2010). The Effect of Bifocal Add on Accommodative Lag in Myopic Children with High Accommodative Lag. *Investigative Opthalmology & Visual Science*, 51(12), p.6104.
6. Cheng, D. (2010). Randomized Trial of Effect of Bifocal and Prismatic Bifocal Spectacles on Myopic Progression. *Archives of Ophthalmology*, 128(1), p.12.
7. Cheng, D., Woo, G. and Schmid, K. (2010). Bifocal lens control of myopic progression in children. *Clinical and Experimental Optometry*, 94(1), pp.24-32.
8. Chung, K., Mohidin, N. and O'Leary, D. (2002). Undercorrection of myopia enhances rather than inhibits myopia progression. *Vision Research*, 42(22), pp.2555-2559.
9. Ehsaei, A., Mallen, E., Chisholm, C. and Pacey, I. (2011). Cross-sectional Sample of Peripheral Refraction in Four Meridians in Myopes and Emmetropes. *Investigative Opthalmology & Visual Science*, 52(10), p.7574.
10. Fan, D., Lam, D., Lam, R., Lau, J., Chong, K., Cheung, E., Lai, R. and Chew, S. (2004). Prevalence, Incidence, and Progression of Myopia of School Children in Hong Kong. *Investigative Opthalmology & Visual Science*, 45(4), p.1071.
11. Guthrie, S. (2011). Controlling lens induced myopia in chickens with peripheral lens designs. *Optometry and Vision Science*, 88 E-Abstract 110421.
12. Holden, B., Fricke, T., Wilson, D., Jong, M., Naidoo, K., Sankaridurg, P., Wong, T., Naduvilath, T. and Resnikoff, S. (2016). Global Prevalence of Myopia and High Myopia and Temporal Trends from 2000 through 2050. *Ophthalmology*, 123(5), pp.1036-1042.
13. Hoogerheide, J., Rempt, F. and Hoogenboom, W. (1971). Acquired Myopia in Young Pilots. *Ophthalmologica*, 163(4), pp.209-215.
14. Ip, J., Huynh, S., Robaei, D., Kifley, A., Rose, K., Morgan, I., Wang, J. and Mitchell, P. (2007). Ethnic differences in refraction and ocular biometry in a population-based sample of 11–15-year-old Australian children. *Eye*, 22(5), pp.649-656.
15. Iwase, A., Araie, M., Tomidokoro, A., Yamamoto, T., Shimizu, H. and Kitazawa, Y. (2006). Prevalence and Causes of Low Vision and Blindness in a Japanese Adult Population. *Ophthalmology*, 113(8), pp.1354-1362.e1.
16. Jung, S., Lee, J., Kakizaki, H. and Jee, D. (2012). Prevalence of Myopia and its Association with Body Stature and Educational Level in 19-Year-Old Male Conscripts in Seoul, South Korea. *Investigative Opthalmology & Visual Science*, 53(9), p.5579.
17. Liu, Y. and Wildsoet, C. (2011). The Effect of Two-Zone Concentric Bifocal Spectacle Lenses on Refractive Error Development and Eye Growth in Young Chicks. *Investigative Opthalmology & Visual Science*, 52(2), p.1078.
18. Liu, Y. and Wildsoet, C. (2012). The Effective Add Inherent in 2-Zone Negative Lenses Inhibits Eye Growth in Myopic Young Chicks. *Investigative Opthalmology & Visual Science*, 53(8), p.5085.
19. Logan, N., Shah, P., Rudnicka, A., Gilmartin, B. and Owen, C. (2011). Childhood ethnic differences in ametropia and ocular biometry: the Aston Eye Study. *Ophthalmic and Physiological Optics*, 31(5), pp.550-558.
20. Mankowska, A., Aziz, K., Cufflin, MP., Whitaker, D., Mallen, EAH. (2012). Effect of Blur Adaptation on Human Parafoveal Vision. *Investigative Opthalmology & Visual Science,* 53(3), p1145-1150. doi: 10.1167/iovs.11-8477.

© WEIRC 2018 – All rights reserved

21. Mutti, D., Hayes, J., Mitchell, G., Jones, L., Moeschberger, M., Cotter, S., Kleinstein, R., Manny, R., Twelker, J. and Zadnik, K. (2007). Refractive Error, Axial Length, and Relative Peripheral Refractive Error before and after the Onset of Myopia. *Investigative Opthalmology & Visual Science*, 48(6), p.2510.
22. Mutti, D., Sholtz, L., Friedman, N. and Zadnik, K. (2000). Peripheral refractive and ocular shape in children. *Investigative Ophthalmology Vision Science*, 41, pp.1022-1030.
23. O'Donoghue, L., McClelland, J., Logan, N., Rudnicka, A., Owen, C. and Saunders, K. (2010). Refractive error and visual impairment in school children in Northern Ireland. *British Journal of Ophthalmology*, 94(9), pp.1155-1159.
24. Quek, T., Chua, C., Chong, C., Chong, J., Hey, H., Lee, J., Lim, Y. and Saw, S. (2004). Prevalence of refractive errors in teenage high school students in Singapore. *Ophthalmic and Physiological Optics*, 24(1), pp.47-55.
25. Rudnicka, A., Kapetanakis, V., Wathern, A., Logan, N., Gilmartin, B., Whincup, P., Cook, D. and Owen, C. (2016). Global variations and time trends in the prevalence of childhood myopia, a systematic review and quantitative meta-analysis: implications for aetiology and early prevention. *British Journal of Ophthalmology*, 100(7), pp.882-890.
26. Ruiz-Pomeda, A., Perez-Sanchez, B., Valls, I., Prieto-Garrido, FL., Gutierrez-Ortega, R., Villa-Collar, C. (2018). MiSight Assessment Study Spain (MASS). A 2-year randomized clinical trial. *Graefe's Archive for Clinical and Experimental Ophthalmology*, 256(5), p1011-1021.
27. Sankaridurg, P., Donovan, L., Varnas, S., Ho, A., Chen, X., Martinez, A., … Holden, B. (2010). Spectacle lenses designed to reduce progression of myopia: 12-month results. *Optometry and vision science : official publication of the American Academy of Optometry*, *87*(9), 631–641. doi:10.1097/OPX.0b013e3181ea19c7
28. Saw, S. (2003). A synopsis of the prevalence rates and environmental risk factors for myopia. *Clinical and Experimental Optometry*, 86(5), pp.289-294.
29. Saw, S. (2006). Ethnicity-specific prevalences of refractive errors vary in Asian children in neighbouring Malaysia and Singapore. *British Journal of Ophthalmology*, 90(10), pp.1230-1235.
30. Smith, E. (2013). Optical treatment strategies to slow myopia progression: Effects of the visual extent of the optical treatment zone. *Experimental Eye Research*, 114, pp.77-88.
31. Smith, E., Hung, L. and Huang, J. (2009). Relative peripheral hyperopic defocus alters central refractive development in infant monkeys. *Vision Research*, 49(19), pp.2386-2392.
32. Tepelus, T., Vazquez, D., Seidemann, A., Uttenweiler, D. and Schaeffel, F. (2012). Effects of lenses with different power profiles on eye shape in chickens. *Vision Research*, 54, pp.12-19.
33. Wallman, J. and Winawer, J. (2004). Homeostasis of Eye Growth and the Question of Myopia. *Neuron*, 43(4), pp.447-468.
34. Wang, D., Chun, R., Liu, M., Lee, R., Sun, Y., Zhang, T., Lam, C., Liu, Q. and To, C. (2016). Optical Defocus Rapidly Changes Choroidal Thickness in Schoolchildren. *PLOS ONE*, 11(8), p.E1161535.
35. Wildsoet, CF., Chia, A., Cho, P., Guggenheim, JA., Polling, JR., Read, S., Sankaridurg, P., Saw, SM., Trier K., Walline, JJ., Wu PC. And Wolffsohn, JS. (2019). IMI-Interventions for Controlling Myopia Onset and Progression Report. *Investigative Opthalmology & Visual Science*, 60, p.106-131.
36. Wong, T., Ferreira, A., Hughes, R., Carter, G. and Mitchell, P. (2014). Epidemiology and Disease Burden of Pathologic Myopia and Myopic Choroidal Neovascularization: An Evidence-Based Systematic Review. *American Journal of Ophthalmology*, 157(1), pp.9-25.e12.
37. Wong, Y. and Saw, S. (2016). Epidemiology of Pathologic Myopia in Asia and Worldwide. *Asia-Pacific Journal of Ophthalmology*, 5(6), pp.394-402.
38. Yang, Z., Lan, W., Ge, J., Liu, W., Chen, X., Chen, L. and Yu, M. (2009). The effectiveness of progressive addition lenses on the progression of myopia in Chinese children. *Ophthalmic and Physiological Optics*, 29(1), pp.41-48.
39. Zheng, Y., Pan, C., Chay, J., Wong, T., Finkelstein, E. and Saw, S. (2013). The Economic Cost of Myopia in Adults Aged Over 40 Years in Singapore. *Investigative Opthalmology & Visual Science*, 54(12), p.7532.

© WEIRC 2018 – All rights reserved
