# Supplementary material for: Spectacle Lenses With Aspherical Lenslets for Myopia Control vs Single-Vision Spectacle Lenses: A Randomized Clinical Trial
Source: JAMA Ophthalmol. 2022 Mar 31;140(5):472–8. doi: 10.1001/jamaophthalmol.2022.0401 (PMC8972151; doi:10.1001/jamaophthalmol.2022.0401)
Supplement: Supplement 2. — Standard Operating Procedures [file jamaophthalmol-e220401-s002.pdf]

Clinical Trial SOP

ESS-TEC-207 FIN

Contents

1. Registration 3
   1.1. Apparatus: 3
   1.2. Procedure: 3
2. Visual Acuity 3
   2.1. Apparatus: 3
   2.2. Procedure: 3
3. Distance Interpupillary Distance (PD) using Pupillometer 4
   3.1. Procedure: 4
4. Distance Subjective Refraction with the Phoropter 5
   4.1. Procedure: 5
5. Cover Test 5
   5.1. Procedure: 5
6. Modified Thorington Test 5
   6.1. Procedure: 5
7. Stereopsis 6
   7.1. Apparatus: 6
   7.2. Procedure: 6
8. Accommodation Measurement 6
   8.1. Procedure: 6
9. Central Axial Length (Lenstar LS900) 7
   9.1. Procedure: 7
10. Peripheral Axial Length (Lenstar LS900) 12
    10.1. Procedure: 12
11. Choroidal Thickness (Topcon OCT) 12
    11.1. Procedure: 12
12. Cycloplegia 12
    12.1. Apparatus: 12
    12.2. Procedure: 12
13. Cycloplegic Autorefraction 13
    13.1. Apparatus: 13
    Topcon KR-800 13
    13.2. Procedure: 13

14. Cycloplegic Peripheral Autorefraction....13
14.1. Apparatus:....13
14.2. Procedure:....13
15. Frame Selection....14
16. Lens Ordering....14
16.1. Procedure:....14
17. Dispensing....15
17.1. Procedure:....15
18. Verification of lenses....15
18.1. Procedure:....15
19. Wearing Instructions....15
19.1. Procedure:....15

1. Registration

1.1.Apparatus:

- Registration List

1.2.Procedure:

1. Subject registration will be done by Dr Jin Wangqin only
2. A new Subject ID code will be given for each new recruit.
3. Should the subject not meet the inclusion criteria, he/she will not be included in the study and the Subject ID code will not be reused for another subject.
4. The registration will be kept confidential as it is the master list will identification of the subjects that registered for the study.
5. This list can only be assessed by the Study Principle Investigator and Study Manager.

2. Visual Acuity

2.1.Apparatus:

- Distance
  - 100% EDTRS contrast chart
  - 10% EDTRS low contrast chart
- Near
  - EDTRS near chart 3
  - 10% SLOAN low contrast near chart

2.2.Procedure:

6. Examination should be performed by a certified examiner.
7. Verify proper chart installation and illumination (Visual projection system): 5.5 metres testing distance and chart background luminance will be 80 cd/m^2
   a. 100% ETDRS chart 1 for right eye
   b. 100% ETDRS chart 2 for left eye
   c. 10% EDTRS low contrast chart for both eyes
8. For near, chart will be 40cm from the eye using the string attached on the chart.
9. As the subject reads the chart, the testing examiner must ensure at all times that the patient is not attempting to attain stenopaeic vision by closing or squeezing the eye in order to enhance acuity.
10. Verify that chart is covered until the patient is seated in position and the non-testing eye is occluded. Please re-cover chart after testing of each eye.
11. Ensure that patients are wearing the required optical correction / no optical correction.
12. Convey to patients that the chart contains letters only (no numbers are used).
13. Instruct patients to start at the top line of the chart, and then read from left to right. Reading pace should be controlled by the examiner not to exceed a letter

per second. When reading place is lost, please ask the patient to go back to the last letter that was identified.

14. **Upon the first error, instruct the patient to start the line all over again; allow 15-20 seconds per letter identification from now onwards.**
15. When patients claim they cannot read a letter, they should be encouraged to provide their best guess, based on the apparent shape of the letter. When a few options are mentioned, patient should be asked to choose or guess – even if the next letter has already been read.
16. Once a patient has identified a letter with a definite single response and has proceeded to the next letter, a correction of previously read letters is neither allowed, nor recorded.
17. Mark the number of letters missed at the respective place for each line on the ETDRS logMAR VISUAL ACUITY WORKSHEET.
18. Testing Endpoint: when 3 or more mistakes are made on a line, elicit responses to all letters on that line but do not continue to the next line".

Example:

If a patient misses two (2) of five (5) letters at the 0.1 logMAR line, and misses three (3) of five (5) letters at the 0.0 logMAR line (which is the smallest line at which any letters were read correctly), then the logMAR VA is calculated as follows:

Baseline VA = 0.0 (last line at which any letters were read)
n = 5 (total number of letters missed on the chart, 2 at the 0.1 line plus 3 at the 0.0 line)
Therefore, the logMAR VA = 0.0 + (5* 0.02) = 0.

The same procedure above will be repeated for 100% and 10% low contrast charts for, under photopic (around 200lux) and mesopic (around 5lux) conditions.

3. Distance Interpupillary Distance (PD) using Pupillometer

3.1. Procedure:

1. Set the measuring distance of the pupillometer at distance (∞).
2. The examiner sits directly in front of the subject at eye level.
3. Both the examiner and the subject hold the pupillometer.
4. Instruct the subject to open his/her both eyes and look at the light in the pupillometer.
5. Occlude the subject's left eye and then move the black vertical line straight at the reflection point of subject's right eye.
6. Occlude the subject's right eye and then move the black vertical line straight at the reflection point of subject's left eye.
7. Record the result of each eye separately.
8. Repeat steps 1 to 7 to measure two or three times and take an average value.

4. Distance Subjective Refraction with the Phoropter

4.1. Procedure:

1. Initial MPMVA
2. Initial Duochrome
3. The Jackson Cross Cylinder (JCC) Test
4. Second Monocular MPMVA
5. Binocular Balance
6. Binocular MPMVA

5. Cover Test

5.1. Procedure:

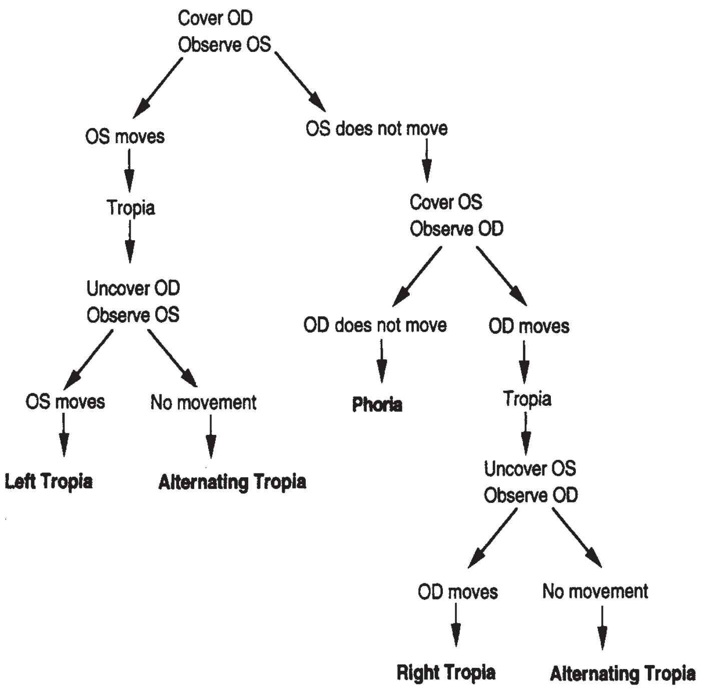

6. Modified Thorington Test (33cm)

6.1. Procedure:

1. Instruct the patient to look at the light in the center of the card.
2. Instruct the patient to tell you the location of the streak relative to the light: to the left, to the right, or through the light for the lateral phoria above, below, or through the light for the vertical phoria.

3. To determine the size of the phoria, ask the patient to tell you the target closest to which the streak passes.
4. Repeat 3 times and record.
5. Record the size and the direction of the phoria.

7. Stereopsis

7.1. Apparatus:

1. Red-green glasses
2. TNO Stereo test booklet.

7.2. Procedure:

1. The subject wears red-green glasses over his/her distance correction.
2. The subject holds the stereo target at 40 cm.
3. The overhead lamp is directed toward the target.

8. Accommodation Measurement

8.1. Procedure:

1. Set the parameter values: 0.01 D, - Cyl, VD=0, 33cm/3D.
2. Disinfect the chin rest and forehead rest.
3. The patient wears full correction with the trial frame.
4. Adjust the height of the patient's chair and the instrument to a comfortable position for both the patient and the examiner.
5. Unlock the instrument controls.
6. Instruct the subject to place his/her chin in the chin rest and his/her forehead against the headrest.
7. Raise or lower the chin rest until the subject's outer canthus is aligned with the pointer on the side of the WAM-5500.

Note:

- Near visual target: a black single Chinese character (equivalent to 0.2 logMAR)
- Near-distance: 33 cm
- Guide the subject to open both eyes to look at the target.
- Only measure the subject's left eye.
- Guide the subject to keep the target clear during the measurements.
- Measure 3 times and take an average value.
- Print the results.

The accommodation response (AR) was also measured in the right eye under bilateral viewing conditions with an open-field autorefractor (WAM-5500, Grand Seiko). The target continuously presented a single Chinese character from a short story with a height of 1.8 mm (0.31°) at the centre of a laptop screen 33 cm in front of the right eye. The autorefractor was set to a high-speed mode to record refractive data at 5 Hz for 60-second continuous measurements. The accommodative stimulus and AR at the corneal plane were calculated using the equations correcting for the effect of the spectacle lenses on autorefractor readings. In terms of accommodative microfluctuation (AMF) calculation, the autorefractor readings from the first ten seconds (50 numerical) and the anomalous autorefractor readings (such as less than -6D, greater than 0D, or the difference of more than 1D in the two consecutive values) were excluded, and the standard deviation (SD) was calculated as AMFs from the remaining data.

The accommodative amplitude (AA) of the right eye was measured by a push-up method using a Royal Air Force (RAF) ruler (Haag-Streit England, Essex). The subjects were instructed to keep the rightmost letter of the smallest line seen of the N-series target on the RAF ruler clear and report the first sustained blur. The distance from infinity to the near point was recorded, and AA was defined as the reciprocal of distance of the near point and was expressed in dioptres (D). Three measurements were recorded and averaged.

9. Central Axial Length (Lenstar LS900)

9.1. Procedure:

Anterior Chamber Depth, Crystalline and Vitreous chamber depth will be measured using Lenstar LS900 for each visit. The result shall be recorded in the Refraction Sheet.

1. Procedure: Turn on the computer, click 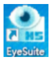 to start lenstar.

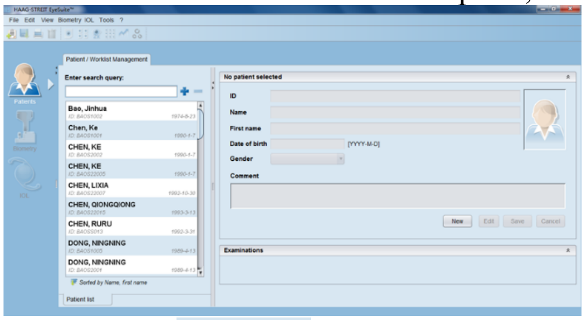

2. Click 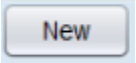 , enter the patient details.

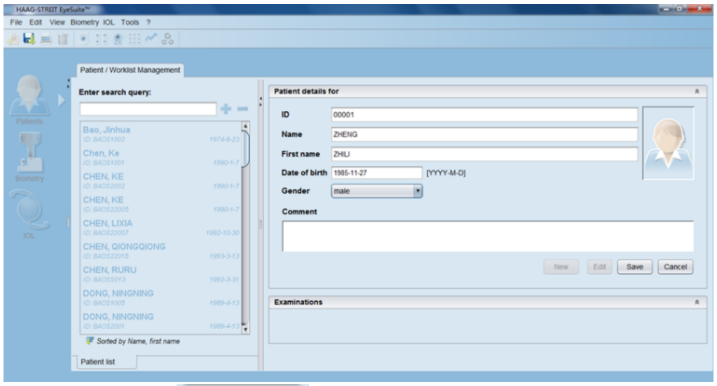

3. Click 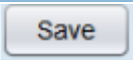

, the follow window is displayed.

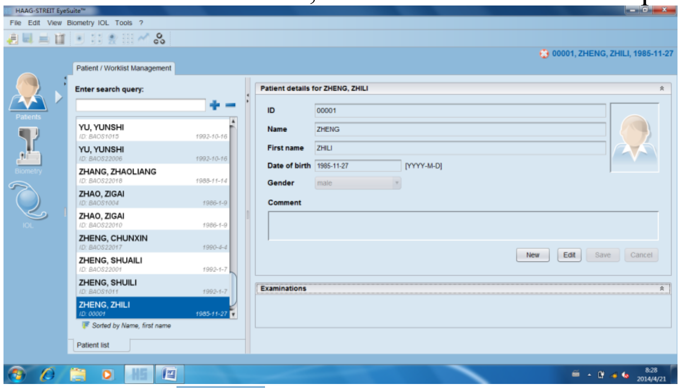

4. Click 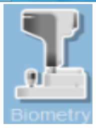

to next.

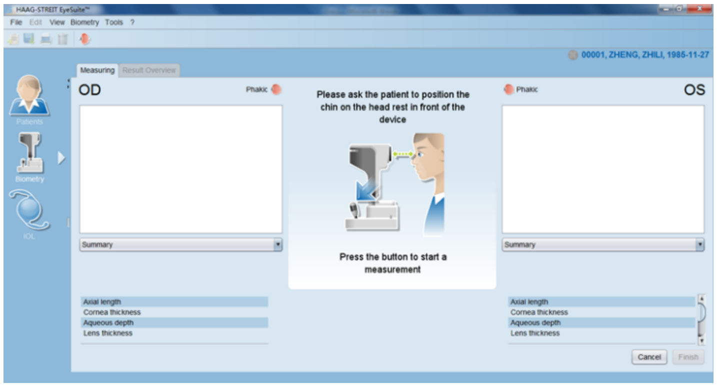

5. Adjust the chin rest height to align the eye with the markings on the head rest columns. Ask the patient to put their forehead against the forehead rest.

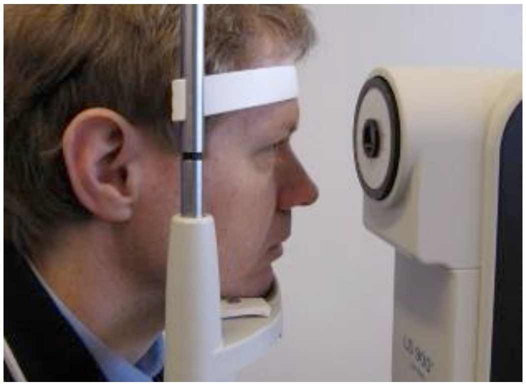

6. Click the joystick button to start the alignment step.

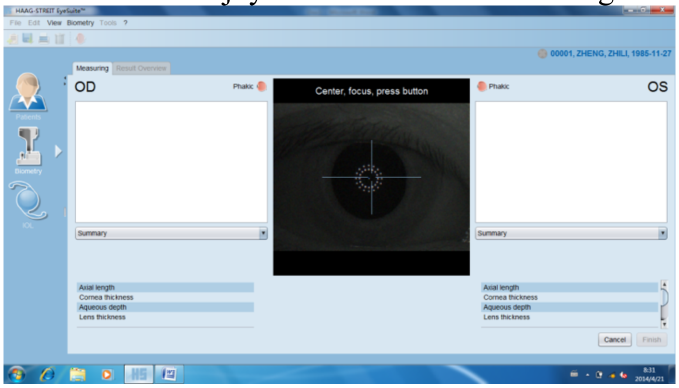

7. Move LENSTAR towards the patient until the reflexes of the keratometry measurement are roughly in focus and keep the joystick in the upright position at the same time. Adjust LENSTAR to center the blinking reflex of the measurement beam in the crosshair as displayed.

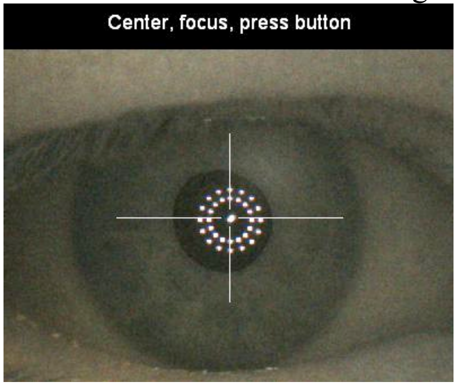

8. Click on the joystick button to switch to zoom mode for fine alignment, use the joystick to adjust the LENSTAR's position until a green circle is displayed.

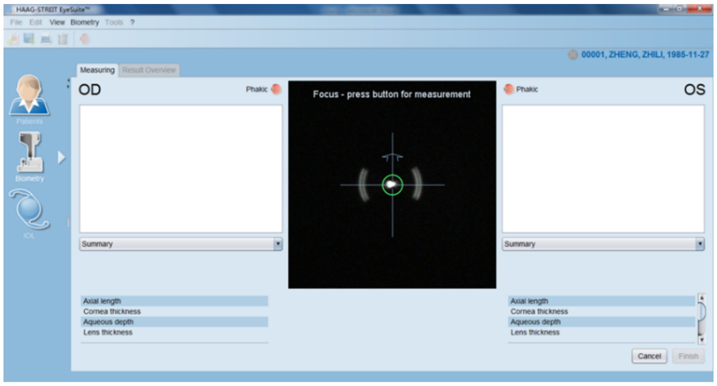

9. Slowly move the joystick in the direction of the arrow until the green circle is the smallest, center the blinking reflex of the measurement beam in the circle as displayed.

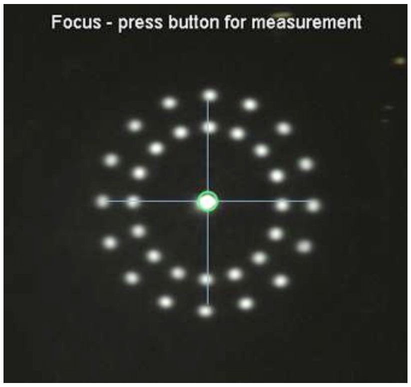

10. Click on the joystick button to start the measurement.

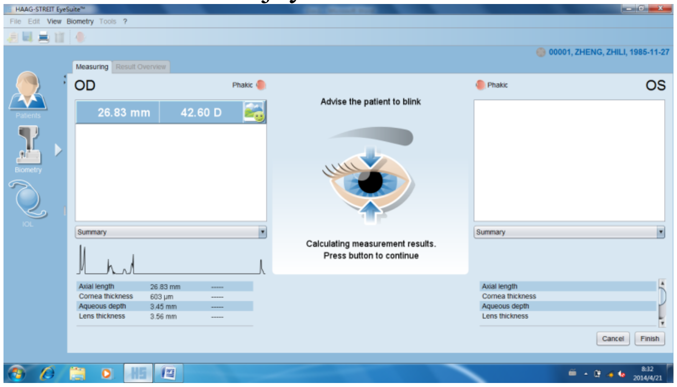

11. click on the joystick button again to start the next time measurement, take 5 measurements in succession, don't move LENSTAR in between successive measurements to speed-up the procedure.

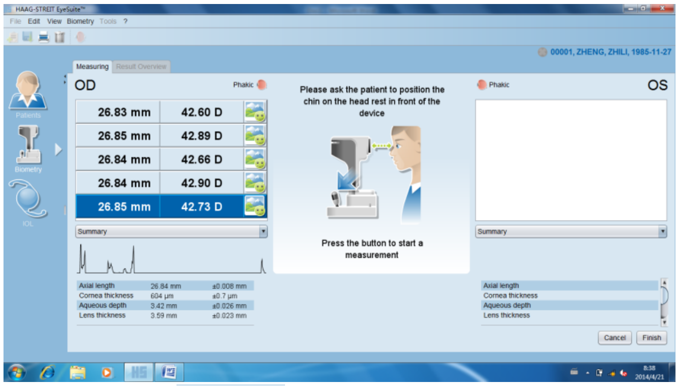

12. Click 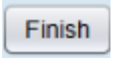 , will see:

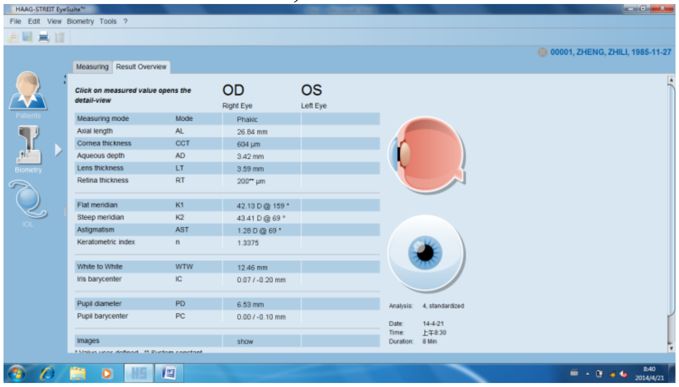

13. from this window, you can get the data of axial length, cornea thickness, aqueous depth, lens thickness, white to white, pupil diameter and so forth.

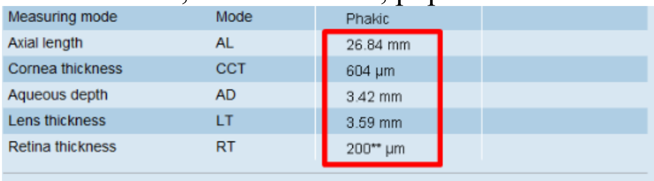

Measuring mode	Mode	Phakic	
Axial length	AL	26.84 mm	
Cornea thickness	CCT	604 µm	
Aqueous depth	AD	3.42 mm	
Lens thickness	LT	3.59 mm	
Retina thickness	RT	200** µm	

14. click any data in this frame, the follow window is displayed. You can get the data of every measurement.

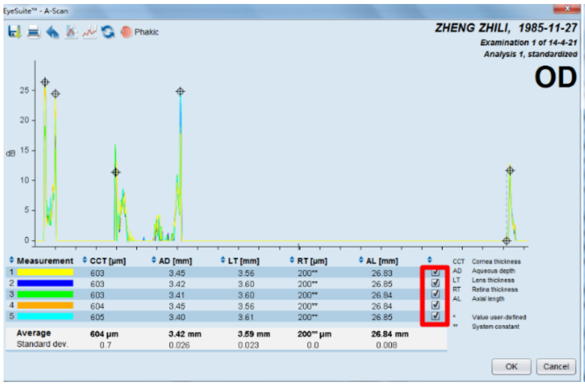

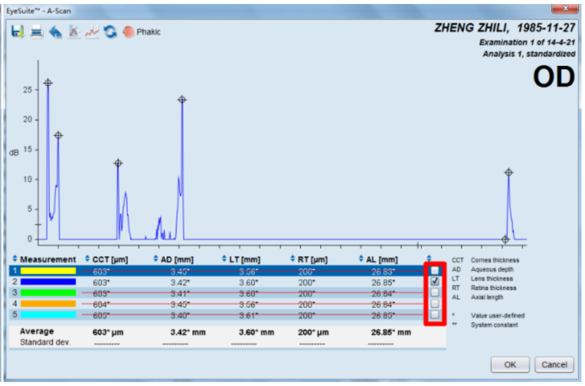

15. Use this picture, you can get the data of retina thickness manually.

16. Click 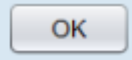 and 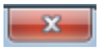 to exit LENSTAR, then turn off the computer.

10. Peripheral Axial Length (Lenstar LS900)

10.1. *Procedure:*

1. Follow set up procedure in Section 8 for Central Axial Length (Lenstar LS900).
2. Patient need to look at Maltese at ±15° and ±30° respectively.
3. 3 measurements will be taken at 15 degrees, 30 degrees nasally and temporally only for right eye.

11. Choroidal Thickness (Topcon OCT)

11.1. *Procedure:*

All participating children undergo macular scan using a swept-source Optical coherence tomography (Topcon OCT). The SF ChT is measured three times with a 9-mm line scan composed of 128 single A-scans. The instrument's follow-up mode is adopted, and built-in software is used to segment layers and construct topographic maps. The SF ChT is determined as the thickness between the outer retinal pigment epithelium and the inner chorioscleral interface. The 12-line radial scans centered on the fovea, each 6-mm in length and separated by 15°, is made three times. Each radial OCT image is constructed from an average of 16 scans. A central 6×6 mm circular region was partitioned automatically according to the Early Treatment Diabetic Retinopathy Study.

12. Cycloplegia

12.1. *Apparatus:*

1. 1% proparacaine
2. 1% tropicamide (if need)

12.2. *Procedure:*

1. Cycloplegia will be induced in each eye by instillation of 1 drop of 1% cyclopentolate,
2. Followed, after 5 minutes, by 1 drop of 1% cyclopentolate,
3. Cycloplegic refraction will take place not less than 30 minutes after the installation of the second drop.
4. Cycloplegic UVA (CUVA) will be performed after full cycloplegia has been achieved, and just prior to refraction.

13. Cycloplegic Autorefraction

13.1. Apparatus:

Topcon KR-800

13.2. Procedure:

Set-Up

- Set the parameter values: 0.01 D, - Cyl.
- Disinfect the chin rest and forehead rest.
- The patient removes his glasses.
- Adjust the height of the patient's chair and the instrument to a comfortable position for both the patient and the examiner.
- Unlock the instrument controls.
- Instruct the subject to place his/her chin in the chin rest and his/her forehead against the headrest.
- Raise or lower the chin rest until the subject's outer canthus is aligned with the pointer on the side of the autorefractor.

Step-by-Step Procedure

1. Move the autorefractor to the left or right by the joystick until the subject's right eye is on the screen.
2. Instruct the subject to look at the visual target in the autorefractor.
3. Refine of the image of the points on the middle of the subject's cornea.
4. Measure three times and take an average value.
5. Repeat steps 1 to 4 to measure the subject's left eye.

Recording

- Record the Rx for each eye separately in standard Rx form.

14. Cycloplegic Peripheral Autorefraction

14.1. Apparatus:

Grand Seiko WAM-550

14.2. Procedure:

1. 10 reading for right eye only will be taken for each meridian.

2. Peripheral target will be 15 and 30 degrees from the central target for all directions, nasal and temporal.
3. Head needs to be rotated to the viewing direction to prevent ocular shape change from extraocular muscles tension. Macfadden et al 2007.
4. Target will tungsten bulbs mounted on each direction.
5. Instructions will be given to look at the target and not focus on it.

Macfadden, L. A., Gray, L. S., Strang, N. C. and Seidel, D.(2007) The effect of eye rotation on measurements of peripheral retinal shape using the IOLMaster. Invest. Ophthalmol. Vis. Sci. 48, E–abstract 4002.

15. Frame Selection

The primary purpose of a spectacle frame is to hold the prescribed lenses in such a way as to provide optimal visual efficiency. Rimless frames are excluded, and the minimum frame height (B) of 30 mm.

In addition, the frames should therefore be physically comfortable, attractive in appearance, and meet the expectations of the subjects.

16. Lens Ordering

16.1. Procedure:

1. The moment the subject has been found eligible for the study, the completed 'Screening and Baseline CRF' must be scanned and sent to the designated person (Lim Ee Woon) for randomization. [limew@essilor.com.sg]
2. The 'Order Form' will be filled up by the Study Coordinator in WEIRC and sent to the designated person.
3. The designated person will start the ordering process after randomization and sent to TES or Creteil France for lens fabrication.
4. TES or Creteil France will fabricate the lens according to the order form and frame model and verify the lenses before sending them to WEIRC.

Name: Huang Ying Ying
Address: Eye Hospital of Wenzhou Medical University
270 Xueyuan West Road
Wenzhou, Zhejiang
China 325027

5. The 'Order Form' and randomization results will be kept confidential from everyone involved in this study till completion.

17. Dispensing

17.1. Procedure:

1. Select the final frame that the subject is to wear and adjust it to fit properly. The procedure is similar to single-vision spectacles.

18. Verification of lenses

18.1. Procedure:

1. Verifying the powers of the lenses.
2. Mark the optical centre of the lenses to check for pupillary distance.

19. Wearing Instructions

19.1. Procedure:

Children have to wear the study glasses during all waking hours except when engaged in swimming or bathing. When swimming, the child can wear goggles with or without correction. Parents and children will be reminded that spectacle wear is expected at school, at home, and during the holiday months. Minimum wearing time of 7 hours per day is required. All children will be receiving the following written instructions:

1. If objects to the side do not appear clear, turn the head sideways until clarity improves.
2. Return immediately for any needed frame adjustments.
3. Do not adjust frames or reinsert lenses at home. All frame adjustments must be done by one of the research staff.
4. Do not use superglue on any part of the frame or lens. If the frame breaks, return to the study center for a replacement.
5. In case of any symptom of an adaptation problem, please contact our optometrist (88068166).
