# Supplementary material for: Spectacle Lenses With Aspherical Lenslets for Myopia Control vs Single-Vision Spectacle Lenses: A Randomized Clinical Trial
Source: JAMA Ophthalmol. 2022 Mar 31;140(5):472–8. doi: 10.1001/jamaophthalmol.2022.0401 (PMC8972151; doi:10.1001/jamaophthalmol.2022.0401)
Supplement: Supplement 3. — Statistical Analysis Plan [file jamaophthalmol-e220401-s003.pdf]

Statistical Analysis Plan

TRIAL FULL TITLE	Myopia Control with Aspherical Lenslets
SAP VERSION	1.0
SAP VERSION DATE	27 Feb 2019
ETHICS APPROVAL DOCUMENT NUMBER	Y2018-054
TRIAL REGISTRATION NUMBER	ChiCTR1800017683
TRIAL STATISTICIAN	
TRIAL CHIEF INVESTIGATOR	
SAP AUTHOR	

1 SAP Signatures

I give my approval for the attached SAP entitled ChiCTR1800017683 SAP dated 17 April 2019.

Chief Investigator

Name:

Signature: ______________________________

Date: ______________________________

Statistician

Name:

Signature: ______________________________

Date: ______________________________

-

2 Table of Contents

1 SAP Signatures 1
• 1
2 Table of Contents 2
3 Abbreviations and Definitions 3
4 Introduction 3
4.1 Preface 4
4.2 Purpose of the analyses 4
5 Study Objectives and Endpoints 5
5.1 Study Objectives 5
5.2 Endpoints 5
6 Study Methods 7
6.1 General Study Design and Plan 7
6.2 Non-Inferiority Studies 8
6.3 Inclusion-Exclusion Criteria and General Study Population 8
6.3.1 Inclusion Criteria 8
6.3.2 Exclusion Criteria 9
6.4 Randomization and Blinding 10
○ Double - masking 10
6.5 Study Variables 10
7 Sample Size 13
8 General Considerations 14
8.1 Timing of Analyses 14
8.2 Analysis Populations 14
8.2.1 Efficacy population 14
8.2.2 Safety Population 14
8.3 Covariates and Subgroups 14
8.4 Missing Data 15
8.5 Interim Analyses and Data Monitoring 15
8.5.1 Purpose of Interim Analyses 15
8.5.2 Planned Schedule of Interim Analyses 15
8.5.3 Scope of Adaptations 15
8.5.4 Stopping Rules 15
8.5.5 Analysis Methods to Minimise Bias 16
8.5.6 Practical Measures to Minimise Bias 16

8.5.7 Documentation of Interim Analyses 16
8.6 Multiple Testing 16
9 Summary of Study Data 16
9.1 Subject Disposition 17
9.2 Protocol Deviations 17
9.3 Demographic and Baseline Variables 17
9.4 Concurrent treatment and Medication 18
9.5 Treatment Compliance 18
10 Efficacy Analyses 18
10.1 Primary Efficacy Analysis 19
10.2 Secondary Efficacy Analyses 20
10.3 Exploratory Efficacy Analyses 20
11 Safety Analyses 20
11.1 Extent of Exposure 20
11.2 Adverse Events 20
11.3 Deaths, Serious Adverse Events, and other Significant Adverse Events ...21
12 References 21

3 Abbreviations and Definitions

Provide a list of the abbreviations and acronyms used in the Statistical Analysis Plan (SAP) with definitions. All terms will appear in alphabetical order.

This section should be completed on an ongoing basis during the preparation of the document and checked carefully after preparing the rest of the SAP to ensure that all the abbreviations are captured.

Although the abbreviations are listed, it is standard practice to spell out abbreviated terms and indicate them in parentheses at their first appearance in the text.

AE	Adverse Event
CRF	Case Report Form
IMP	Investigational Medical Product
SAP	Statistical Analysis Plan

4 Introduction

This Statistical Analysis Plan (SAP) provides a detailed and comprehensive description for analyzing the myopia control efficacy study coded TEX-207 FIN. The purpose of the study is to assess the efficacy of SAL or HAL lenses has better efficacy in slowing down the progression of myopia compared to single vision

spectacle lenses. The study design is described in the Protocol and the trial registered with ChiCTR.org.cn (ChiCTR1800017683).

The analysis will be presented in a report, which will be used as the basis of the primary research publications according to the study publication plan. This SAP describes the statistical methods for the primary, secondary, and exploratory outcomes of the study as defined in the protocol, as well as additional subgroup analysis.

4.1 Preface

Currently, myopia is managed mainly by inducing hyperopic defocus by either using contact lenses like orthokeratology and multifocal soft contact lenses and spectacles lenses like progressive lenses, bifocals, and peripheral defocus lenses. Other methods like atropine are also used to control myopia. The methods mentioned above have various efficacy in controlling myopia, with low dose atropine being the most effective at 59%, followed by orthokeratology with about 43% of myopia control and multifocal soft contact lenses at 49% of myopia control. These methods are invasive and may not be suitable for all young children. Moreover, the rebound effect for atropine was not desirable upon cessation of drug use. For spectacle correction method, bifocals have the best outcome of about 45% myopia control. However, with more studies with animal to understand the effect of optical effect has on myopia progression, better myopia can be achieved with a new spectacle lens design (Wildsoet et al., 2019).

It has been shown that a persistent myopic defocus constantly applied on the whole retina has a positive effect on myopia progression, i.e., decrease in myopia progression (Anstice and Phillips, 2011). Based on the above background and outcome of several studies on optical treatment on myopia progression, we developed 2 FIN spectacle lenses described in Section 6.1.1 of the Protocol.

4.2 Purpose of the analyses

These analyses will assess the efficacy and safety of SAL and HAL compared to Single Vision Spectacle Lenses (SVL) and will be included in the clinical study report.

5 Study Objectives and Endpoints

5.1 Study Objectives

The study aims to evaluate the efficacy of spectacle lenses with aspherical lenslets to reduce the progression of myopia by either reducing the myopia progression rate per year and/or reducing the elongation of eyeball through myopic defocus compared with SVL group. 2 embodiments of the spectacle lenses with aspherical lenslets will be tested and compared with single vision spectacles lenses (SVLs) as a control. 150 healthy children aged 8 to 13 years old will be recruited in a double-masked randomized clinical trial over a wearing period of at least two years. Cycloplegic autorefraction and axial length will be the primary measure for myopia progression. Other measures like peripheral autorefraction will be done to evaluate the effect of myopic defocus on peripheral retina. Finally, visual acuity and contrast sensitivity will also be compared between spectacle lenses with aspherical lenslets and SVL to quantify the quality of vision using myopia control lenses like FIN.

5.2 Endpoints

OBJECTIVES	ENDPOINTS	JUSTIFICATION FOR ENDPOINTS
Primary		
The objective of the study is to evaluate the efficacy in controlling myopia progression assessed by measuring cycloplegic autorefraction and axial length of SAL and HAL compared to SVL. This aim will be achieved by conducting a randomized clinical trial which will compare myopic progression in children wearing Spectacle lenses with aspherical lenslets vs. children wearing SVL. The comparison will allow the quantification of the effect of Spectacle lenses with aspherical lenslets on myopia progression during the two-year follow-up.	0.50D difference in myopia progression in at least one eye after two years compared to control arm using spherical equivalent of cycloplegic autorefraction (SER) AND change in axial length (AL) converted to diopters using conversion of 0.10mm = 0.28D in at least one eye and one test arm compared to control arm. Or 33% myopia control for AL and SER in two years.	Walline et al., 2018 agreed that 30-50% less progression in the test arm compared with control or reduction in progression of more than 0.75D difference over 3 years is a viable effectiveness threshold.

Secondary		
To evaluate the visual performance of SAL and HAL compared to SVLs such as high and low contrast visual acuity under photopic and mesopic conditions. Moreover, this study aims to assess visual comfort using questionnaires with Spectacle lenses with aspherical lenslets compared to SVL.	• 80% or equivalent compliance and adaptation in at least one test arm compared to control arm.	Efficacy, safety, and adherence of test arm achieving primary endpoint was based on previous studies on spectacle solution for myopia control with about 50% of test arm having an effective slower rate of progression in myopia, and this test device is expected to perform better. 80% compliance and adaptation to treatment is considered better than the average of 68% in most clinical trials (Czobor & Skolnick, 2011).
**Exploratory**		
To explore if at least one test arm could have an effect on peripheral cycloplegic autorefraction and axial length in one eye and/or have an effect on choroidal thickness in one eye when compared to control arm.	• To have less negative peripheral spherical equivalent of cycloplegic autorefraction at nasal 30° in at least one eye in one test arm.<br>• And/or choroidal thickness change should be the same or less changes compared to control arm having more significant thinning in at least one eye in one test arm.	Radhakrishnan et al. 2013 found 0.24D change in peripheral refraction at nasal 30° over the course of years for test and control group with myopia progression. Having less than 0.24D change in peripheral refraction will show a positive effect of test arm on peripheral retina. Similarly,

		statistically significantly less choroidal thinning compared to control group is a positive effect of test arm.

6 Study Methods

6.1 General Study Design and Plan

This is a monocenter, randomized, double-masked controlled-group study to evaluate if spectacle lenses with aspherical lenslets can slow down the progression of myopia. 150 healthy children aged between 8 to 13 years old with myopia of -0.75D to -4.75D (spherical equivalent) and meeting the specific inclusion and exclusion criteria will be recruited. Consent will be taken from guardians and children who are eligible for the study, and they will be randomized using Randola to wear either of the SAL, HAL (test arm), single vision lenses (control arm). All children will be followed up 6 monthly for 2 years to monitor changes in cycloplegic autorefraction and axial length. At the end of the study, the above measures will be compared between test and control arm for objective and subjective performance.

Each subject will be followed for at least 2 years and 3 weeks.

The ophthalmic lenses will be worn by subjects and allocated according to a randomization ratio of 1:1:1 in each arm (150/3 in each arm).

The study will be conducted at the Eye Hospital of Wenzhou Medical University (Wenzhou Medical University-Essilor International Research Centre, WEIRC), Wenzhou city, Zhejiang province, China, following the regulations and hospital IRB policies that follows the Tenets of the Declarations of Helsinki.

Figure 1. Study diagram

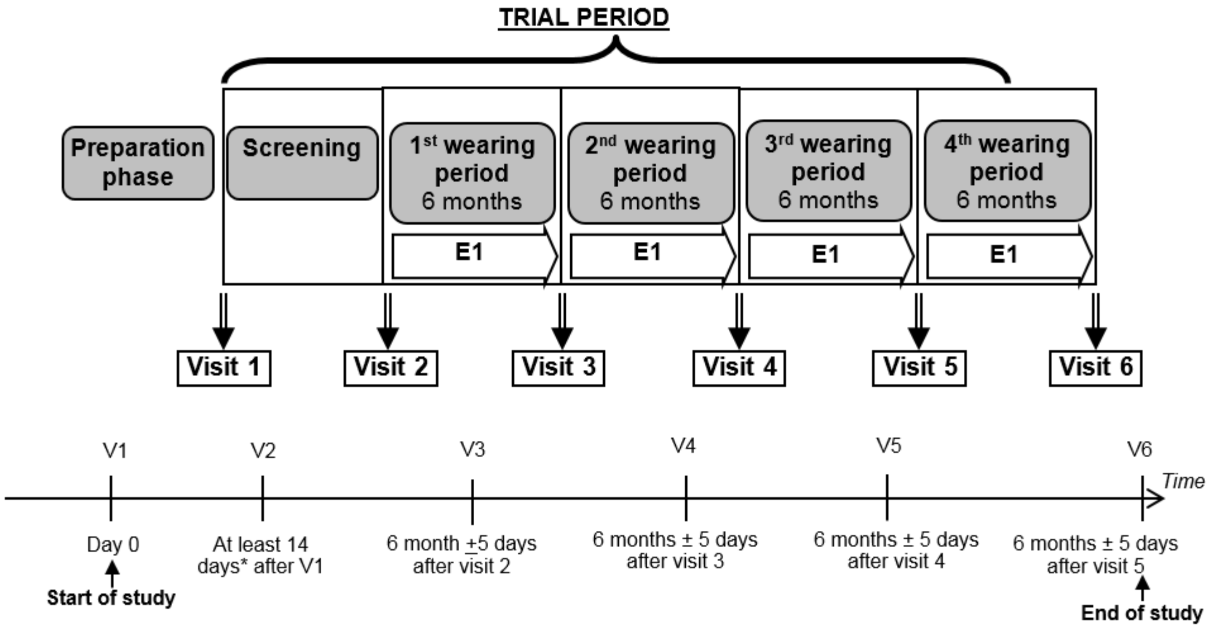

6.2 Non-Inferiority Studies

Superiority:

- Hypotheses: SAL or HAL > SVL, P-value 0.05
- Superiority bound(s):
  - 95% confidence interval for upper and lower bounds at 5% level of significance for:
    - Cycloplegic spherical equivalent
    - Axial length
- Population: Intention-to-Treat

6.3 Inclusion-Exclusion Criteria and General Study Population

6.3.1 Inclusion Criteria

Subjects will be included in the study if they satisfy the following criteria:

General inclusion criterion:

- Volunteer subject and guardian, fluent Chinese spoken, willing to follow the protocol, and able to read, comprehend and sign the informed consent form.

Study related inclusion criteria:

- Age: equal to or greater than 8 years and not older than 13 years.
- Spherical refractive error of -0.75 to -4.75 D in each eye (spherical equivalent), as measured by cycloplegic autorefraction.
- Astigmatism of not more than 1.50 D.
- Anisometropia of not more than 1.00 D.
- Best-corrected visual acuity of equal or better than 0.05 LogMAR (≥ 0.9 as Snellen)
- No strabismus by cover test at near and distance.
- Have the ability to comply with the protocol to get the reliable study measurements.
- Absence of ocular disease with a full ophthalmic examination, such as retinal disease, cataract, and ptosis. Good general health, without systemic or neurodevelopmental conditions. Without ocular or systemic medicine, which might affect myopia progression or visual acuity through known effects on retina, accommodation, or significant elevation of intraocular pressure.
- No history of PALs or bifocals use and no prior use of contact lenses or any treatment for myopia control.

6.3.2 Exclusion Criteria

Subjects presenting with any of the following exclusion criteria will not be included in the study:

General exclusion criteria:

- Vulnerability of the subject,
- Participation in another study which might have an influence on vision or interfere with study assessments,

Study related exclusion criteria:

- Age: less than 8 years old or greater than 13 years.
- Spherical refractive error: less than -0.75 D or greater than -4.75 D in each eye (spherical equivalent), measured by cycloplegic autorefraction.
- Astigmatism of more than 1.50 D.
- Anisometropia of more than 1.00 D.
- Best-corrected visual acuity of less than 0.05 LogMAR (≤ 0.8 as Snellen)
- With strabismus by cover test at near or distance.
- Without the ability to comply with the protocol to get the reliable study measurements
- Presence of any ocular disease that would influence refractive development, such as retinal disease, cataract, and ptosis. Presence of systemic or neurodevelopmental conditions that may influence refractive development. Use of ocular or systemic medicine, which might affect myopia progression or visual acuity through known effects on retina, accommodation, or significant elevation of intraocular pressure.
- Prior use of progressive adaptive lenses, bifocals use, use of contact lenses, or any treatment for myopia control.

6.4 Randomization and Blinding

Scheduled randomization will be generated by the Study Manager in charge of lens logistics using online application Randola and forwarded **only** to the person in charge of fabrication in Essilor (France or Singapore). The Investigators will never have access to the randomization list. Randomization is based on spherical equivalent of cycloplegic autorefraction in right eye, age, and gender.

Each time a subject becomes eligible for the study, he/she will be randomly assigned to an arm in a ratio of 1:1:1, then the Investigator will receive the study device from the person in charge of fabrication accordingly.

Once the database has been locked, and populations for analysis agreed, all codes (subject identification and randomization) will be re-checked.

○ Double - masking

The Investigator, subjects, and study personnel of the study center will be masked to the trial equipment worn. Examiners measuring primary outcomes have no contact with study equipment from the study or the participants.

The pockets containing the lenses will be the same for both Reference and Test lenses. The lens packaging design will be created in order to dispense the correct equipment to each subject according to the randomization list.

In the event of early discontinuation of the study or in case of emergency, the Investigator will ask the study manager in charge of lens logistics to break the masking, but only if it is considered mandatory for a further major reason. The fact that a subject reaches an endpoint is not a reason for breaking the masking.

The Investigator should record the reason for the date and time of breaking, his/her name, and function in the study together with his/her signature. The date and reason for the unmasking must also be documented in the source document and the Case Report Form.

6.5 Study Variables

Describe the frequency and timing of all the relevant variable observations or assessments. A table or flow chart may be appropriate, for example

Visit schedule	V1 (2hr)	V2 (45min)	V3 (1:25hr)	V4 (1:45hr)	V5 (1:25hr)	V6 (1:45hr)
Informed Consent [5min]	X					
Eye examination (non cycloplegic) [30min]	X		X	X	X	X
Checking of inclusion / exclusion criteria	X					
Central axial length (Lenstar) [5min]	X		X	X	X	X
Peripheral axial length (Lenstar) [10min]	X					X
Cycloplegic subjective refraction [60min]	X		X	X	X	X
Cycloplegic Central autorefraction (Topcon) [10min]	X		X	X	X	X
Cycloplegic Peripheral autorefraction (Grand Seiko) [30min]	X					X

Choroidal Thickness (OCT) [10min]	X			X		X
Study frame choice [10min]	X		X	X	X	
Study frame adjustment [10min]	X		X	X	X	
Monocular pupillary distances and fitting measurements [5min]	X		X	X	X	
1st Equipment (E1) delivery		X				
Photopic & Mesopic Visual acuity measurements (Near & Distance 10%, 100% contrast) [15min]		X	X	X	X	X
Stereoacuity @40cm [5min]		X		X		X
Accommodation @33cm[T] (Grand Seiko) [10min]		X		X		X
Monocular and Binocular Amplitude of accommodation [5min]	X	X	X	X	X	X
Visual comfort questionnaire [10min]	X		X	X	X	X

Screening & Baseline

- Study explanation (reading of information sheet of the Informed Consent with the subject)
- Agreement for subject participation in the study (signing of the Informed Consent Form)
- Eye examination:
    - Subject’s demography: gender, date of birth
    - Parents’ demography: myopia status, occupation, education level
    - Case history: general health, ocular health, and history, medication
    - Current glasses description: date of prescription, wearing time per day and week, prescription, visual acuities, fitting measurements, lenses description
    - Preliminary examinations: complaint screening
    - Binocular vision measurements: suppression screening, dissociated phoria, strabismus screening
    - New prescription: auto-refractometer measurement, refraction, visual acuities
        - Study frame choice
        - Study frame adjustment
        - Monocular pupillary distances and fitting measurements
- Checking of inclusion / exclusion criteria
- Investigator decision to include the subject into the study
- Baseline examination
    - Visual comfort questionnaire
    - Monocular amplitude of accommodation
    - Choroidal thickness
    - Axial length measure (central and peripheral)
    - Anterior chamber depth, vitreous chamber depth, corneal thickness & lens thickness
    - Cycloplegic autorefraction (central and peripheral)

After the screening visit, the study PI will check all the eligibility criteria and be in charge of the definitive inclusion of the subject into the study. The study manager in charge of lens logistics will do the randomization after definitive inclusion.

The eye examination results and other information must be present in source documents at the study center.

- Equipment (E1) delivery

At least 2 weeks ± 3 days after visit 1, 3, 4, 5

- Documentation of Adverse Events*
- Equipment (E1) delivery
  - Check frame adjustment
  - Check lens fitting
- Test with Equipment (E1)
  - Photopic & Mesopic visual acuity measurements 10%, 100%, distance and near
  - Stereoacuity
  - Accommodation at 33cm
  - Monocular amplitude of accommodation

Between visit 2 and visit 3, a recommendation for the subject is to wear E1 for more than 6 hours per day, every day of the week.

- Equipment (E1) Evaluation

At least 6 months ± 5 days *after visit 2, 3, 4, 5*

- Documentation of adverse events*
- Test with Equipment (E1):
  - Visual comfort questionnaire
  - Photopic & Mesopic Visual acuity measurements 10%, 100%, distance and near
- Subjective Refraction
- Axial length measure
- Anterior chamber depth, vitreous chamber depth, corneal thickness & lens thickness
- Cycloplegic autorefraction
- Choroidal Thickness

For all visits, a recommendation for the subject is to wear E1 more than 6 hours per day, every day of the week. May need to arrange a visit 2 weeks later should there be a need to change the test lenses to a new prescription.

- Visit 6: Equipment evaluation / Final follow-up

At least 6 months ± 5 days *after visit 5*

- Documentation of adverse events*
- Test with Equipment (E1)
  - Visual comfort questionnaire

    - Photopic & Mesopic Visual acuity measurements 10%, 100%, distance and near
    - Stereoacuity
    - Accommodation at 33cm
    - Monocular amplitude of accommodation
- Subjective Refraction
- Choroidal thickness
- Axial length measure (central and peripheral)
- Anterior chamber depth, vitreous chamber depth, corneal thickness & lens thickness
- Cycloplegic autorefraction (central and peripheral)

** The occurrence of Adverse Events (AEs) since the last visit will be determined by the subject's spontaneous reporting, the Investigator's non-leading questioning, and his/her evaluation. All AEs will be reported in the Case Report Form (See section 8.3 Adverse Events in Protocol).*

**Variables**	**Endpoints**
Primary	
Central axial length (Lenstar)	0.27mm difference from control or 33% less than control, $P < 0.05$, 2-tailed
Cycloplegic Central autorefraction (Topcon)	0.50D difference from control or 33% less than control, $P < 0.05$, 2-tailed
Secondary	
Visual comfort questionnaire	80% compliance
Photopic & Mesopic Visual acuity measurements (Near & Distance 10%, 100% contrast)	No difference from control, $P > 0.05$, 2-tailed
Average wearing time	No difference from control, $P > 0.05$, 2-tailed
Exploratory	
Choroidal Thickness (OCT)	No change or better than control
Cycloplegic Peripheral autorefraction (Grand Seiko)	less negative than control
Peripheral axial length (Lenstar)	less negative than control

7 Sample Size

This section should reproduce the relevant section from the protocol. If any amendments to the sample size have been made during the study, these should be documented and explained here. If any techniques are used to adjust the primary analysis for sample size adjustment, they should be described in the relevant section (9.2 in Protocol).

With an expected 33% reduction in mean myopia progression in Spectacle lenses with aspherical lenslets compared with SVLs, a study by Yang et al. found a mean myopia progression of 1.50 D over 2 years in Chinese children wearing SVLs. A 33% treatment effect over 2 years is, therefore about 0.50D.

An overall standard deviation (s.d.) of 0.6D for the magnitude of myopia progression in two years was derived from the study in myopia progression of PALs in Chinese children by Yang et al. [32]. We estimated a higher error in our study and increased the standard deviation to 0.75 with the following determinant.

1. At least a 90% statistical power.
2. Type 1 error probability (α) of 0.05, based on a two-tailed t-test.
3. 1:1:1 sample ratio.
4. Adjustment for multiplicity of 2 endpoints.
5. Allowing a maximum drop-out rate of 10%

$$k = n_2/n_1 = 1$$

$$n_1 = (\sigma_1^2 + \sigma_2^2/K) *(z_{1-\alpha/2} + z_{1-\beta})^2/\Delta^2$$

$$n_1 = (0.75^2 + 0.75^2/1) *(1.96 + 1.28)^2/0.5^2$$

$$n_1 = 47$$

$$n_2 = K*n_1 = 47$$

$$n_1 = 47*10\% \sim 50$$

3 treatment groups = 50 * 3 = 150

8 General Considerations

8.1 Timing of Analyses

The interim analysis at 1 year and a final analysis at 2 years will be performed when 150 subjects have completed visit 6 or when 33 has dropped out prior to visit 6 and when data transferred to the file FIN Data.xlsx, having been documented as meeting the cleaning and after the finalization and approval of this SAP document.

8.2 Analysis Populations

8.2.1 Efficacy population

The intention-to-treat approach will be used in the analysis for all subjects who received any study device. The principle of intention-to-treat will be the main strategy of analysis adopted for primary and secondary endpoints. These analyses will be conducted on all patients assigned to the treatment groups as randomized regardless of the study treatment received.

8.2.2 Safety Population

For the analysis of safety outcomes using exploratory endpoint, all subjects who received any study treatment (including control) but excluding subjects who drop out prior to receiving any treatment are included as in the intention-to-treat approach.

8.3 Covariates and Subgroups

Provide a general comment identifying the covariates (continuous or categorical, including subgroups) that are expected to have an important influence on specific endpoints (e.g., demographic or baseline measurements, concomitant therapy). Document any model selection procedures (e.g., forward stepwise selection).

Subgroup analysis will only be done for variables that were not randomized equally. The main variables for randomization were cycloplegic autorefraction, age, and gender, which are important variables for primary endpoint. The other variable that will be included in subgroup analysis will be axial length should there be any between group difference during baseline.

Other demographic variables like age of myopia onset, parental myopia, height, and weight will be analyzed for their effect on myopia progression.

8.4 Missing Data

No missing data imputation will be done.

8.5 Interim Analyses and Data Monitoring

Interim analysis will be done after 1year follow-up. Blind analysis will be carried out by a third-party statistician that is independent with no interest and involvement in the clinical trial. Results from the blind analysis will be shared with the Data and Safety Monitoring Committee (DSMC). DSMC will advise if the clinical trial is safe to be continued.

Blind analysis is done by removing all identifiable variables like subject identification and birth dates after including the masked concepts variable by the one personnel with the masked information. The data will be scrambled before it is passed to the third-party statistician for analysis.

8.5.1 Purpose of Interim Analyses

The purpose of Interim Analysis is to have early decisions for safety, endpoint, and for future planning of resources.

The safety of treatment will be assessed based on adverse events reporting, changes from baseline through cycloplegic autorefraction, and axial length.

Efficacy of treatment will be based on differences in cycloplegic spherical equivalent and axial length with control group.

8.5.2 Planned Schedule of Interim Analyses

There will only be 1 interim analysis at 1 year follow-up.

8.5.3 Scope of Adaptations

Change in study design like switching inferior treatment arm to a more superior one or control will be employed should there be a safety issue in the interim analysis.

8.5.4 Stopping Rules

Trial will be stopped if cycloplegic autorefraction or axial length of treatment progressed 10% worse than control.

8.5.5 Analysis Methods to Minimise Bias

Blind analysis will be done by a third-party statistician with no interest and involvement in the study.

8.5.6 Practical Measures to Minimise Bias

Below are the measures in conducting interim analysis:

- Only third-party statistician will perform any interim analysis
- Data and Safety Monitoring Committee will see any data or analyses in the interim and advise the study PI. The PI will make decisions for the next phase of clinical trial.
- Treatment safety will be publicly available following an interim analysis.
- Treatment safety, efficacy, or futility will be provided to the PI and investigators.
- Only 1 study manager in charge of lens logistics is unmasked at any point in the trial.
- Third-party statistician will perform any final analyses, and all investigators will remain masked.
- If any, safety monitoring decision making will remain isolated from efficacy information.

8.5.7 Documentation of Interim Analyses

The analysis plan and program, and interim analysis report will be stored as part of the documentation.

8.6 Multiple Testing

With 2 treatment groups, Bonferroni adjustment will be used to adjust the p-value and statistical outcome when comparing with control group.

9 Summary of Study Data

All continuous variables will be summarised using the following descriptive statistics: n (non-missing sample size), mean, standard deviation, standard error, maximum and minimum. The frequency and percentages (based on the non-missing sample size) of observed levels will be reported for all categorical measures. In general, all data will be listed, sorted by treatment and subject, and when appropriate by visit number within-subject. All summary tables will be structured with a column for each treatment in the order (Control, Treatment) and will be annotated with the total population size relevant to that table/treatment, including any missing observations.

9.1 Subject Disposition

The following diagram will be filled up at the end of the study.

Subject Disposition Flow Diagram

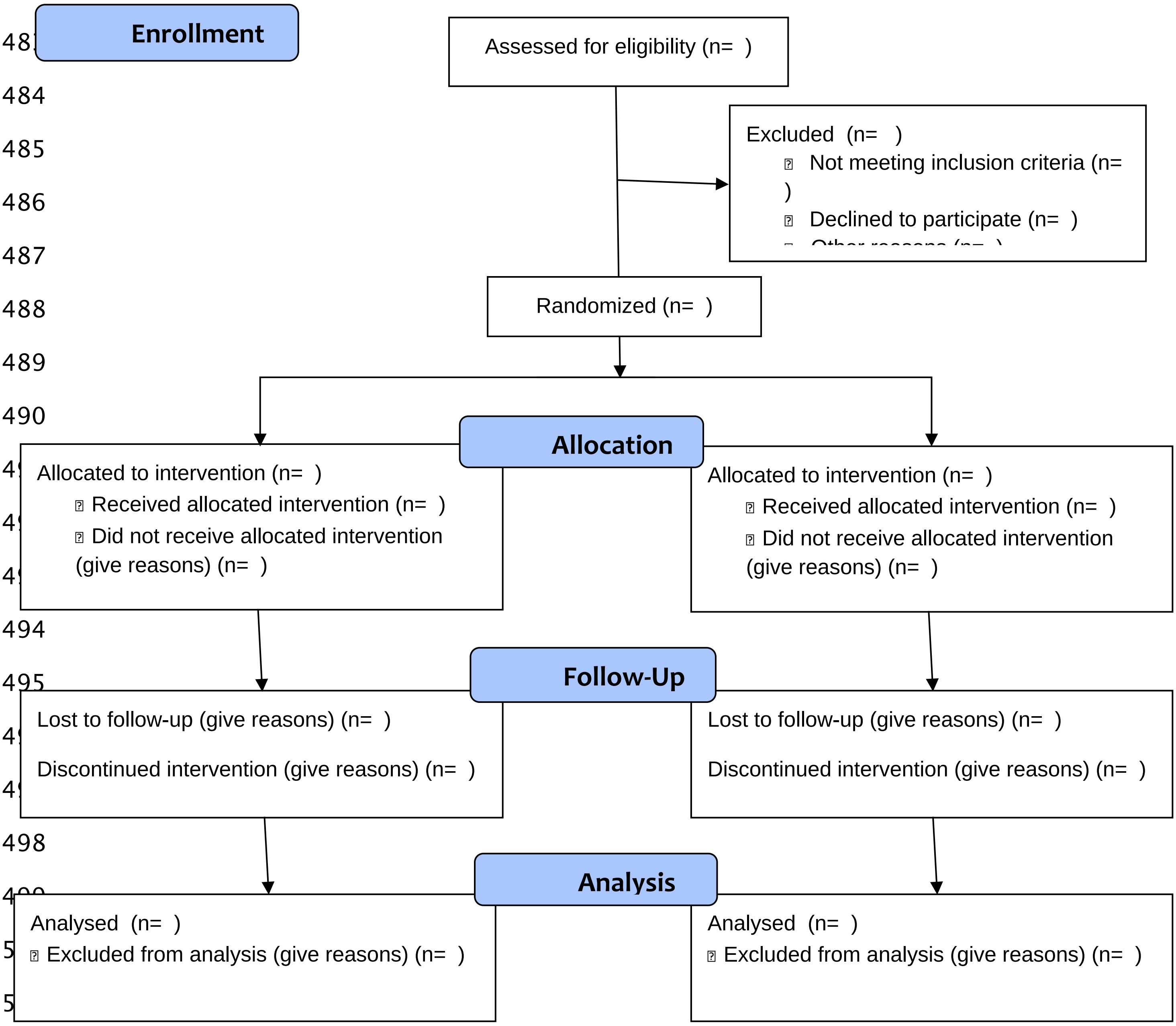

9.2 Protocol Deviations

Protocol deviation will be reported to the DSMC to decide if the subject should be excluded from the analysis.

9.3 Demographic and Baseline Variables

Demographic variables:

Age
Height
Weight
Age of myopia onset
Education level
Parental myopia
Hobbies

Baseline variables:

Cycloplegic autorefraction
Subjective refraction
Axial length
Amplitude of Accommodation
Accommodation at 33cm
Visual Acuity
Stereoacuity
Peripheral refraction
Peripheral Axial Length
Choroidal Thickness

The summary statistics will be produced in accordance with section 10.

9.4 Concurrent treatment and Medication

This will be reported in the summary statistics.

9.5 Treatment Compliance

Compliance with intervention is monitored using questionnaires collected during Visit 3, 4, 5, and 6. In addition, phone interviews will be done 3 days, 2 weeks, and 3 months post dispensing after every visit. The main measure of compliance is the number of days the investigative device was worn and the number of hours worn each day. **Total compliance** is defined as 7 hours a day for 7 days a week, equivalent to 49 hours of wearing a week. Based on total hours of wearing in a week, **Actual compliance** will be actual wearing duration in a week, and **Individual compliance** is defined below:

Individual compliance = (Actual compliance / Total compliance) * 100%

Study compliance will be the percentage of study participants that manage to comply at least 80% of Individual compliance, defined below:

Study compliance = (Individual compliance => 80% / Total study population) * 100%

10 Efficacy Analyses

All efficacy variables will be listed by subject. Data will be summarized by treatment group. N, Mean, Standard Deviation, Standard Error, Minimum, and Maximum will summarise continuous efficacy variables, whereas number and percent will summarise categorical efficacy variables.

All analyses of the continuous efficacy variables (e.g., cycloplegic autorefraction and axial length) will be performed as analysis of variance with treatment group. Treatment groups will be tested at the 2-sided 5% significance level.

All assumptions for regression models will be assessed by viewing plots of the residual values

All analyses of categorical efficacy measures will be performed using logistic regression with the treatment group and adjustments for variables statistically different in each group.

10.1 Primary Efficacy Analysis

Spherical equivalent will be calculated from sphere and cylinder measured in Diopters (D) on an interval scale and computed as sphere + cylinder /2. Axial length will be measured in millimeters (mm) on an interval scale.

Progression of spherical equivalent and axial length will be defined as the change in spherical equivalent from the baseline within each crossover stage and will be computed for each participant-eye. Progression of spherical equivalent and axial length will be analyzed within a stage in a grouped format and between stages in a paired format.

For the progression variables, normality of the underlying mean distribution will be assumed based on central limit theorem as spherical equivalent, and axial length are measured on an interval scale and the study sample being >30. Graphical representation of the raw progression data between groups and stages will be done using Box and Whisker plots. Descriptive statistics such as mean, median, standard deviation, standard error, minimum, maximum will also describe the raw progression data between groups and stages.

A linear mixed model that accounts for both fixed and random factors will analyze progression in a grouped format. Study group will be modelled as a fixed factor. Subject intercepts will be used to account for the correlation of 2-eye data. Confounders such as age, gender, parental myopia, and baseline refractive error could be used as covariates in the model. Model-based estimated means for each study group that is adjusted for confounders with its 95% confidence limits will be reported.

Spherical equivalent and axial length between-group will be analyzed ANOVA with 1 treatment comparing to 1 control group. The order of assignment will be accounted for in the model and tested for significance. Confounders such as age, gender, parental myopia, and baseline refractive error could be used as covariates in the model.

Model-based estimated means for each study group adjusted for confounders with its 95% confidence limits will be reported.

These analyses will be performed on the (1) Intent to treat analysis datasets and (2) Per-protocol analysis dataset.

Individual data points that are missing will be excluded from analysis involving only those specific variables. A participant's complete visit data will not be excluded if some of the observations are missing. Inclusion of outliers in the analysis will be based on the magnitude of change in test statistics with and without the outliers. Outliers will preferably be retained unless there is a significant change in test results. Any outliers removed from analysis will be reported along with reasons for exclusion. In conjunction with the PI, the study optometrist may label a study visit as "non-evaluable" on any protocol deviations as deemed appropriate. This would then be used to exclude the study visit from the analysis.

Two-tailed distributions will be used for inferential statistics. Level of significance will be set at 5%. Any post hoc multiple comparisons will be adjusted using Bonferroni correction to ensure the family wise error rate is set at 5%.

10.2 Secondary Efficacy Analyses

Proportion of population without myopia progression will be compared with control group. Proportion with 80% compliance with device will also be compared with control group using Fisher exact test base on outcome of questionnaires.

Wearing time is measured using 6-monthly questionnaire. Average wearing hours was calculated by taking the weekly average between 6 month and 24 month questionnaires divide by 7 days in 1 week.

Group comparison of near, distance visual acuity and wearing time will be done using ANOVA, which includes study group. means for each study group with its 95% confidence limits will be reported.

Graphical representation of visual acuity data between groups will be done using line graph or Box and Whisker plots. Descriptive statistics such as mean, median, standard deviation, minimum, maximum will also be used to describe the raw accommodation data between groups.

10.3 Exploratory Efficacy Analyses

Exploratory outcomes like peripheral autorefraction and axial length and choroidal thickness will be compared with control group using ANOVA after adjusting for confounders.

11 Safety Analyses

It will be based on adverse events and serious adverse events reporting.

11.1 Extent of Exposure

Changes in cycloplegic autorefraction and axial length will be plotted against wearing period to understand the extent of exposure.

11.2 Adverse Events

When calculating the incidence of adverse events, or any sub-classification thereof by treatment, time period, severity, etc., each subject will only be counted once, and any repetitions of adverse events will be ignored; the denominator will be the total population size.

11.3 Deaths, Serious Adverse Events, and other Significant Adverse Events

When calculating the incidence of serious adverse events, deaths, or any sub-classification thereof by treatment, time period, severity, etc., each subject will only be counted once, and any repetitions of adverse events will be ignored; the denominator will be the total population size.

12 References

White IR and Thompson SG (2005). Adjusting for partially missing baseline measurements in randomized trials. *Statistics in Medicine,* **24,** 993-1007
