# Supplementary material for: Spectacle Lenses With Aspherical Lenslets for Myopia Control vs Single-Vision Spectacle Lenses: A Randomized Clinical Trial
Source: JAMA Ophthalmol. 2022 Mar 31;140(5):472–8. doi: 10.1001/jamaophthalmol.2022.0401 (PMC8972151; doi:10.1001/jamaophthalmol.2022.0401)
Supplement: Supplement 4. — Data Sharing Statement [file jamaophthalmol-e220401-s004.pdf]

Data Sharing Statement

Bao. Spectacle Lenses With Aspherical Lenslets for Myopia Control vs Single-Vision Spectacle Lenses. *JAMA Ophthalmol*. Published March 31, 2022.
doi:10.1001/jamaophthalmol.2022.0401

Data
Data available: No

Additional Information
Explanation for why data not available: Anonymised data will be made available with a data dictionary defining each field upon request.
